# A multimodal manual therapy, exercise, and psychological intervention for painful diabetic neuropathy: the NeuOst feasibility randomised controlled trial

**DOI:** 10.1097/PR9.0000000000001498

**Published:** 2026-09-16

**Authors:** David Hohenschurz-Schmidt, Whitney Scott, Annina B. Schmid, Suzie Cro, Philip Bright, Jerry Draper-Rodi, David W. Evans, Matthew C. Evans, Harriet Kemp, Nuno Koch Esteves, Elizabeth Pigott, Sasha Smith, Siobhan Stynes, Sam Tan, Jan Vollert, Jan D. Wandrey, Esther Williamson, Esther M. Pogatzki-Zahn, Andrew S.C. Rice, Steven Vogel

**Affiliations:** a Imperial Clinical Trials Unit, School of Public Health, Imperial College London, London, United Kingdom; b INPUT Pain Management Unit, Guy's and St Thomas' NHS Foundation Trust, London, United Kingdom; c King's College London, Health Psychology Section, Institute of Psychiatry, Psychology, and Neuroscience, London, United Kingdom; d Nuffield Department of Clinical Neurosciences, Oxford University, John Radcliffe Hospital, Oxford, United Kingdom; e UCO School of Osteopathy, Health Sciences University, London, United Kingdom; f National Council for Osteopathic Research, c/o Health Sciences University, London, United Kingdom; g School of Sport, Exercise and Rehabilitation Sciences, University of Birmingham, Edgbaston, Birmingham, United Kingdom; h Division of Anaesthetics Pain Medicine & Intensive Care, Imperial College London, London, United Kingdom; i THERMOSENSELAB, Skin Sensing, Research Group, School of Health Sciences, The University of Southampton, Southampton, United Kingdom; j Patient Partner, London, United Kingdom; k Section of Vascular Surgery, Department of Surgery and Cancer, Imperial College London, London, United Kingdom; l Imperial Vascular Unit, Imperial College Healthcare NHS Trust, Charing Cross Hospital, London, United Kingdom; m School of Medicine, Keele University, United Kingdom; n Midlands Partnership University NHS Foundation Trust, Staffordshire, United Kingdom; o Department of Clinical and Biomedical Sciences, Faculty of Health and Life Sciences, University of Exeter, Exeter, United Kingdom; p Charité—Universitätsmedizin Berlin, Corporate Member of Freie Universität Berlin and Humboldt-Universität zu Berlin, Department of Anaesthesiology and Intensive Care, Campus Charité Mitte and Campus Virchow-Klinikum, Berlin, Germany; q Berlin Institute of Health at Charité—Universitätsmedizin Berlin, BIH Biomedical Innovation Academy, BIH Charité Digital Clinician Scientist Program, Berlin, Germany; r Nuffield Department of Orthopaedics, Rheumatology and Musculoskeletal Sciences, University of Oxford, Oxford, United Kingdom; s Public Health and Sports Science, University of Exeter, Exeter, United Kingdom; t Department of Anaesthesiology, Intensive Care and Pain Medicine, University Hospital Muenster, Muenster, Germany; u Division of Anaesthetics Pain Medicine & Intensive Care, Department of Surgery and Cancer, Imperial College London, London, United Kingdom

**Keywords:** Diabetic neuropathies, Chronic pain, Randomised controlled trials, Musculoskeletal manipulations, Rehabilitation, Mind-body therapies

## Abstract

**Introduction:**

People with painful diabetic neuropathy lack effective and tolerable treatments. Physical and psychological interventions may help but require further study and combination to maximise effects.

**Objectives:**

This single-site, 3-arm, parallel, randomised feasibility trial tested whether a multimodal intervention combining manual therapy, exercise, and psychologically informed conversations (“NeuOst”) was feasible, and a full-scale trial possible.

**Methods:**

Adults with painful diabetic neuropathy were randomised 1:1:1 to either (1) 5 individual sessions of NeuOst, (2) 5 sessions of a high-similarity control intervention with the same osteopaths, or (3) usual care. Allocation was computer-generated, and blinding was intended for assessors, research and administrative staff, and participants receiving NeuOst or the high-similarity control intervention. Feasibility and acceptability were assessed using predefined criteria. Exploratory clinical outcomes included pain, disability, and quality of life, collected up to 16 weeks post-randomisation.

**Results:**

Thirty-six participants were randomised; targets were met for eligibility (64%), consent (88%), retention (89%), blinding, fidelity, data completeness, and acceptability. Mean NeuOst session attendance was 82%, and satisfaction was high (mean 4.8/5). No serious adverse reactions occurred. Minor adverse responses were reported by 25% of NeuOst and 42% of control intervention participants. Recruitment rates indicated that modified procedures would be needed for a larger trial. Clinical outcomes were uncertain compared with control and promising compared with usual care.

**Conclusion:**

The NeuOst intervention was safe, acceptable, and feasible. The trial design proved largely workable, with areas for refinement identified. Findings support progression to an effectiveness trial.

## Abstract 2

Supplemental Digital Content is Available in the Text.

A multimodal intervention for people with painful diabetic neuropathy proved feasible and acceptable in a feasibility trial, with encouraging clinical signals compared with usual care.

## 1. Introduction

The global burden from painful diabetic polyneuropathy (pDPN) is increasing due to rising diabetes rates and ageing populations. Between 30% and 50% of individuals living with diabetes are estimated to experience diabetic peripheral neuropathy (DPN),^24,72^ where nerve damage can lead to paraesthesia, numbness, and reduced thermal sensation, nociception, and proprioception.^1^ In diabetes, neurologial and microvascular compromise lead to ulcerations that can go undetected, leading to amputations and contributing to mortality.^36,48^ Twenty-five percent or more of people with peripheral neuropathy also have neuropathic pain.^44,72^ This is a highly distressing symptom, described by one of our patient partners as “walking on burning-hot sand and unable to get off it”^40^ (a representative description).^8^ Neuropathy and pain not only affect physical functioning but also sleep, social relationships, and mental health.^20,50,68^

Current neuropathic pain care relies heavily on medications,^17,57,59^ despite their limited efficacy and considerable side effects.^79^ Reflecting its impact, finding effective treatment options for painful DPN is a top priority for patients and health professionals.^29,65^ People with lived experience emphasise the additional need to consider neuropathy in the context of living with diabetes—itself a burdensome health challenge.^40^

Physical and psychological interventions are potential alternatives for people experiencing neuropathic pain from DPN, allowing for person-centred care and consideration of biopsychosocial challenges. These interventions provide small-to-moderate symptom relief and quality of life gains in chronic pain conditions,^13,85,93^ including neuropathic pain.^4^ Growing evidence shows benefits of exercise in DPN and other peripheral neuropathies, including for nerve fibre regeneration, symptomatic relief, quality of life, functional improvement, and blood glucose control^2,45,53,73,75,81^ and suggests short-term effectiveness of manual therapy.^34^ Combinations of aerobic, strength, and balance training appear most promising,^58,81^ although existing studies are often small and heterogeneous.^46,61,71^ Because physical, psychological, and social challenges interact in pDPN, combinations of physical and psychological components may offer the most meaningful and sustainable outcomes. However, few multimodal interventions have been developed for individuals with pDPN.

We developed the multimodal intervention “NeuOst,” combining manual therapy, exercise, and psychologically informed strategies, delivered by specifically trained UK osteopathic manual therapists. Intervention development, content, and methods of the below feasibility trial are detailed elsewhere.^40^ Before evaluating treatment effects in a full-scale trial, key uncertainties and motivators for this study included the feasibility of delivering the NeuOst treatment as well as the feasibility of a randomised controlled trial (RCT). The primary objectives of this feasibility trial were thus to ascertain the feasibility of (1) delivering the NeuOst intervention and (2) conducting a 3-armed, parallel, partially blinded RCT of NeuOst as an adjunct to patients' usual care compared with a high-similarity control intervention^41^ and compared with usual care only. To test feasibility, we prespecified feasibility outcomes as defined in the following section. We also gathered clinical outcome data—including harms—to inform future research steps but did not power the study to detect clinical changes.^40^

## 2. Methods

This trial is reported in accordance with the 2025 CONSORT Statement^43^ and CONSORT extensions for nonpharmacological therapies^9^ and pilot and feasibility trials.^25^ Interventions are described using TIDieR^37^ and the CoPPS checklist for reporting of control interventions in efficacy and mechanistic trials of physical, psychological, and self-management therapies^41^—with completed reporting checklists appended (Supplemental Digital Content, SDC, https://links.lww.com/PR9/A444).

### 2.1. Preregistration

The trial was registered on ClinicalTrials.gov on 15 May 2024 (NCT06423391), after enrolment of the first 4 participants (first participant enrolled on 30 April). Registration was completed following administrative delays during study start-up procedures. The study protocol and statistical analysis plan had been finalised before participant enrolment and remained unchanged until registration, and ethics approval had been obtained. No outcome data were accessed or analysed before trial registration. To enhance transparency, the full protocol and analysis plan were registered on the Open Science Framework on May 29, 2024 (https://osf.io/jyftb).

### 2.2. Sponsor and ethics

The sponsor institution was the Health Sciences University (HSU)—UCO School of Osteopathy, and its institutional research ethics committee approved the study on 05 April 2024 (no identification numbers used by this committee).

### 2.3. Funders

The trial was funded by *The Osteopathic Foundation* and *The Alan and Sheila Diamond Charitable Trust*, with funders having had no role in the conduct, analysis, or write-up of the study.

### 2.4. Patient and public involvement

People with lived experience were consulted extensively during the development of the NeuOst intervention and were involved in the piloting of the control intervention and the training of providers—as described elsewhere.^40^ During the trial, a permanent patient partner (E.P.) was part of the combined Trial Steering and Data Monitoring and Ethics Committee (TSC/DMEC). Results were discussed and interpreted with a board of 7 people with lived DPN experience, diverse regarding ethnicity, age, sex, language, and cultural background. Discussions informed this write-up, and E.P. co-authored this manuscript. Dissemination includes public events in the local community and direct communication with study participants.

### 2.5. Trial design

**Figure 1.**
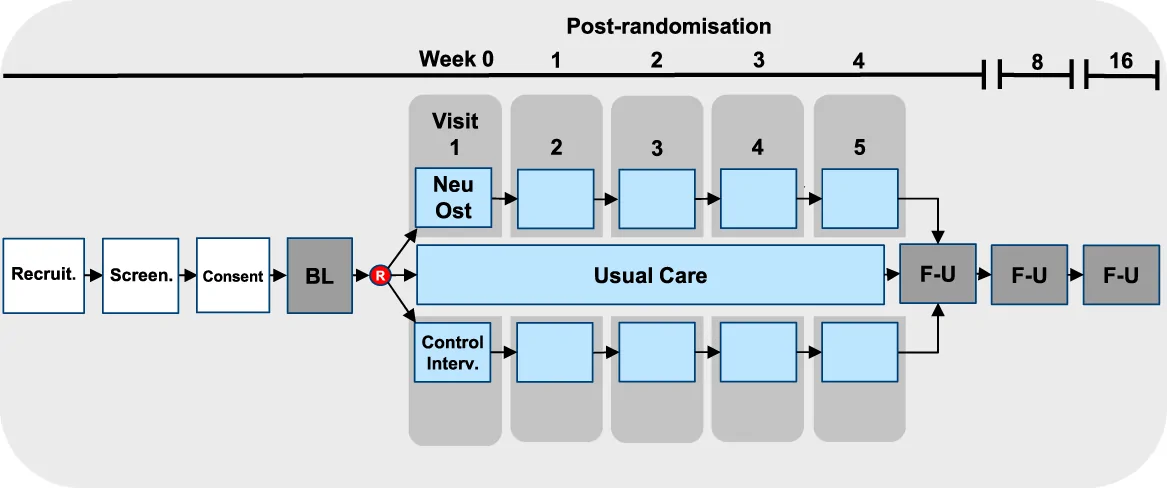
Design of the NeuOst feasibility trial. Interventions are described in detail in the manuscript text and the published protocol, with “control intervention” referring to a specifically designed intervention replicating the tested (“NeuOst”) intervention but omitting components the effects of which were to be studied in the trial. All 3 trial groups continued their usual care outside the trial. BL, baseline assessment; F-U, follow-up assessment; Recruit., recruitment; Screen., screening.

This was a prospective, randomised, parallel-arm, three-group, controlled, partially blinded, clinical feasibility trial, with a 16-week follow-up period and 1:1:1 allocation ratio (Fig. 1). The study was not powered to detect intervention effects, with clinical outcomes collected for exploratory reasons only.

### 2.6. Trial setting

The trial was conducted in London, United Kingdom, at a single outpatient site: the teaching clinic of the sponsor institution (postcode SE1). Treatments were provided in individual rooms, equipped with a treatment plinth and seating.

### 2.7. Recruitment and consent

Between April and December 2024, patient recruitment was conducted through a multichannel strategy including local print and online media (5 adverts), institutional newsletters (7, including 1 to 10,000 members), and at least 12 social media posts. Flyers were distributed across more than 30 sites, mainly in South-East London, including clinics, libraries, pharmacies, churches, and community centres, with additional direct outreach to UK National Health Service (NHS) diabetes and General Practitioner (GP) teams, local charities, and community organisations. Note that recruitment strategies did not include formal NHS recruitment such as identification of potential participants from health records or during routine appointments. Instead, we only used flyers in public spaces of relevant NHS services (GP and intermediate care diabetes services). Towards the end of the year, connections were also made with local community champions active in diabetes advocacy.

### 2.8. Eligibility criteria

#### 2.8.1. Participants

Adults with pDPN were eligible, irrespective of most comorbidities and medication use. Participants had to have a diagnosis of diabetes, and score ≥4 points on the Michigan Neuropathy Screening Instrument (MNSI, questionnaire part)—indicating likely peripheral diabetic neuropathy based on patient-reported symptoms.^33^ Second, a score of ≥3 was required for the Douleur Neuropathique 4 (DN4, interview part), evaluating typical patient-reported pain descriptors and associated symptoms typical for neuropathic pain, and applied to the legs or feet.^80^ To simplify recruitment processes, we screened patients using phone interviews and did not use physical examinations to determine eligibility.

Patients were ineligible if manual therapy and conservative pain management were contraindicated, if they had past or scheduled amputations, or if changes to exercise or pain management regimens had been made in the past month or were planned for the next 3 months. To facilitate blinding, we also excluded patients who reported knowing any other trial participants. For details and a full list of criteria, see the public trial protocol (https://osf.io/jyftb).

#### 2.8.2. Providers and provider training

Trial providers were fully qualified, registered osteopaths with at least 1 year's clinical experience but no prior expertise with neuropathy or diabetes populations. Providers had to have received training to deliver the NeuOst and the control intervention as part of an expert-delivered training course and pretrial methodological workshops. The intervention training course encompassed at least 7 hours of self-study through recorded lectures and reading materials and 8 hours of in-person practical training. Competency was assessed through quizzes and observation after the course, and providers received regular supervision during the trial (training and supervision described in Ref. 40). In addition, trial-specific methodological provider training included education about RCT methods, the purpose of control interventions and blinding, control intervention co-design, and practical training with patient partners in NeuOst and control intervention delivery.

#### 2.8.3. Settings

No specific eligibility criteria existed for settings.

### 2.9. Intervention and comparators

All study arms are described in detail in the preregistered protocol (https://osf.io/jyftb)^40^ and in supplementary treatment manuals (SDC, https://links.lww.com/PR9/A444).

#### 2.9.1. NeuOst intervention (tested intervention)

The intervention, NeuOst, was developed following the MRC framework for complex interventions,^76^ using a logic model informed by literature, expert input, patient involvement, and iterative testing. Content was refined through multidisciplinary collaboration and feedback from people with lived experience.^40^

NeuOst combines manual therapy techniques with psychologically informed pain management derived from Acceptance and Commitment Therapy,^56^ and exercise rehabilitation, plus neurological testing as well as diabetes and foot care education and screening. Fully qualified osteopaths delivered NeuOst in up to 5 one-to-one sessions, with the first session lasting 90 minutes and followed by four 60-minute sessions. All sessions were guided by a treatment manual that specified core and optional components, dosage, and personalisation options.

Acceptance and Commitment Therapy (ACT)-derived elements were structured around weekly worksheets with scripted ACT-derived experiential/reflective exercises and metaphors (see SDC, https://links.lww.com/PR9/A444), including those aimed at fostering present-moment awareness, openness, values exploration, and values-based goal setting—completed in conversation with providers. Of note, while this element of the intervention was guided by principles of ACT, it was not intended to be a complete ACT intervention. Five supervision sessions with the trial clinical psychologist, W.S., and the providers were conducted. These focused on reflection, supporting clinicians' conversations with patients in an ACT-consistent manner and managing challenges arising during the delivery of ACT-derived strategies.

Manual therapy included prolonged articulation of lower-extremity joints, active-resisted muscle activation, soft tissue mobilisation, and neurodynamic techniques. Although delivered by osteopaths, the core techniques of the manual therapy protocol were delivered in a manner not specific to any particular manual therapy profession.^22^

Exercise elements were practised with participants in clinic and performed during a progressive home-exercise programme. An inflatable balance cushion was provided to each participant. Participants were instructed to perform the stretch, strength, and stability elements of the programme at least once per day and engage in a personalised amount and intensity of cardiovascular activity 3 times per week, agreed through values-based goal setting with providers. Participants received a printed exercise programme, including for daily activity tracking.

Printed leaflets were also provided and used to support conversations on the importance of foot care and exercise in diabetes.

#### 2.9.2. Control intervention

The control intervention was delivered by the same set of trial providers. It aimed at blinding participants and all staff (except providers) to NeuOst vs control intervention allocation, and at creating comparable placebo effects to enable efficacy testing of the NeuOst treatment. It was designed according to best-practice guidance,^41^ and developed and piloted with providers and patient partners. The control was designed to match the NeuOst intervention in the following features: number, duration and frequency of sessions, assessment procedures, protocol standardisation and flexibility, opportunities for intervention tailoring and participant participation, application modes (conversational, manual, and self-performed physical), delivery formats, thematic content of conversations and informational material, body areas addressed, physical procedures performed or undergone and sensations experienced (with below exceptions), equipment employed, fidelity monitoring methods, treatment environment, personal interactions with staff and providers, provider characteristics and behaviour, follow-up frequency and mode, and co-interventions (education, foot and neurological screening) (see SDC, https://links.lww.com/PR9/A444 for detail).

The following components differed and were thus supposed to be tested by this trial design: NeuOst activities derived from ACT were replaced by generic discussions of a patient's pain experience and their current self-management strategies, structured around specifically developed worksheets to resemble the ones used to deliver ACT activities. Manual therapy techniques were applied with reduced range and minimal force, and neurodynamic techniques with reduced range. Exercise promotion was limited to education and exploration of exercise barriers, the provision of an inflatable balance cushion—which was also provided in the NeuOst arm—and advice about specific exercises as per therapist preference but was not structured through a formal exercise plan.

This control intervention design sought to avoid core ACT mechanisms related to psychological flexibility (behaviour that is “aware, open, and engaged”), limit the activation of joint receptors through end-of-range movement, relaxation of muscles, or mobilisation of nerves, and avoid promoting behaviour change toward engagement in a structured programme of progressive aerobic and anaerobic physical activity.

We sought to reduce group contamination by not recruiting participants who knew of other study participants (assessed through patient self-report) and by avoiding overlapping appointment times. All providers had co-developed the control intervention and were trained—including in the scientific rationale for control interventions and blinding (SDC, https://links.lww.com/PR9/A444). Further details and a description of intervention similarity are provided in Reference 40.

#### 2.9.3. Usual care

Control and NeuOst interventions were delivered as an adjunct to patients' usual care, which participants continued outside the trial. Participants randomised to the dedicated usual care arm did not receive care as part of the trial and only participated in outcome collection calls.

All participants were asked not to change their medical or nonpharmacological pain or neuropathy care routine although doing so did not lead to withdrawal from the study.

### 2.10. Fidelity promotion and monitoring

Fidelity to NeuOst and control intervention protocols was measured by a provider-completed checklist for each attended session (SDC, https://links.lww.com/PR9/A444). Resource constraints prevented the independent verification of intervention delivery. At regular supervision sessions with the chief investigator (D.H.S.) and trial psychologist (W.S.), challenges with NeuOst and control intervention delivery were discussed, and delivery harmonised where possible. Participant adherence to interventions was not enhanced beyond routine encouragement from clinicians and check-in calls if appointments were missed. To assess the content of “usual care,” healthcare utilisation in the usual care arm was evaluated through patient report (as it was in all other arms).

### 2.11. Outcomes and data collection timepoints

#### 2.11.1. Feasibility outcomes

Feasibility was judged according to several feasibility outcomes, following prespecified criteria (see protocol^40^ and section 3). Feasibility outcomes were rates of the following:

(1) Eligibility
(2) Recruitment
(3) Consent
(4) Blinding
(5) Intervention fidelity in treatment delivery
(6) Data completeness
(7) Participant retention
(8) Adverse events
(9) Treatment acceptability (through satisfaction ratings, session attendance rates, and qualitative feedback)

Most feasibility outcome data, such as attendance of clinical sessions or outcome assessments, were collected continuously through study administrative logs.

Blinding was assessed after the final treatment visit, and intervention acceptability and satisfaction were assessed after the final visit and the final follow-up (week 16).

#### 2.11.2. Clinical and exploratory outcomes

Clinical outcome data were collected through phone or video calls with blinded outcome assessors at the following timepoint: at baseline, after the final treatment session (or at week 5 for usual care arm participants, 3 weeks thereafter [week 8], and after another 8 weeks [week 16; all through calls] [Fig. 1 and protocol]).^40^ Participants received £15 per completed study assessment call. The longest follow-up was approximately 16 weeks after randomisation.

For the 2 groups attending clinic appointments (NeuOst and control intervention), clinical outcomes were also collected before each clinical visit using patient self-report on paper case report forms.

Owing to the feasibility nature of the trial, clinical outcomes were secondary, collected to evaluate their suitability as primary outcome measures in a full-scale trial. This objective also explains the partial overlap, eg, between some disability and quality of life measures. Collected clinical outcomes were as follows:

(1) Patient-reported pain severity and interference (measured using the Brief Pain Inventory, short form)^83^
(2) Disability (Diabetic Peripheral Neuropathic Pain Impact measure [DPNPI])^10,11^
(3) Neuropathy-specific quality of life (Neuropathy- and foot ulcer-specific quality of life instrument [NeuroQoL])^89^
(4) Fear of falling (Falls Efficacy Scale International, Falls Efficacy Scale (FES-I), 7-item short form [Tinetti et al., 1990])^86^
(5) Number of days with “bothersome” and “intolerable” pain in past week (approach adapted from^31,64^ and [Markman et al., 2020])^54^
(6) Physical activity levels (trial-specific exercise compliance form)
(7) Global impression of change (Patient's Global Impression of Change scale [Dworkin et al., 2005])^23^
(8) Concomitant medication and intervention use (using a form adapted from NIH NCCIH Prior and Concomitant Medications and Interventions Form)

Furthermore, healthcare utilisation and receipt of concomitant care were monitored during assessment calls in all groups—including health education, physical and psychological care, and performance of exercise.

To explore participants' physical activity levels and as a potential indicator of adherence to the exercise programme, we collected data on activity levels at each follow-up timepoint, including before each clinic visit of the NeuOst and control intervention groups. Data were collected as participant-reported time spent doing either any strength, stretch, or stability exercises, or any cardiovascular activity.

Expectations and treatment credibility were assessed to supplement blinding data. Data were collected at baseline (ie, before randomisation and based on information available to participants at that time about the NeuOst intervention, eg, from the Participant Information Sheet) and at visit 2 in the NeuOst and control intervention arms (ie, after 1 exposure to the allocated intervention). To do so, we used the Credibility and Expectancy Questionnaire^7,21^ and the Stanford Expectations of Treatment Scale (SETS).^96^ The Credibility and Expectancy Questionnaire was analysed by converting percentage scores of items 4 and 6 into 1 to 9 Likert format ((X/100) × 8 + 1) and by averaging the credibility items (1–3) and expectancy items (4–6) to obtain subscores, with higher scores indicating higher credibility and expectancy, respectively. Assessed using the SETS, positive and negative treatment expectations were calculated as means of SETS items 1, 3, and 5 and 2, 4, and 6, respectively, with values ranging from 1 (strongly disagree) to 7 (strongly agree), with 4 meaning “neither agree nor disagree.”^96^ SETS item 10 was framed to evaluate if participants had experienced “osteopathy” before.

### 2.12. Harms

Adverse events and reactions were defined in the protocol, including their severity and potential relation to study procedures. Participants were asked systematically about any adverse health changes at each clinical appointment and each outcome assessment, in addition to being able to report harms to the study team by phone and email as they occurred.

### 2.13. Sample size and power

Twelve participants per arm was considered to be the minimum number to make meaningful inferences about key feasibility parameters and to assess variance of clinical effects and inform progression to a full-scale trial.^14^

The study was not powered to detect statistically significant differences in effects between intervention arms, and no conclusions about clinical effects should be drawn.

### 2.14. Randomisation

Blocked randomisation was used throughout enrolment and at the end of the trial to ensure balance in the number of participants across the 3 trial arms (1:1:1 allocation ratio). Random permuted block sizes of 3 or 6 participants were used in an unpredictable order to limit predictability of allocations. Participants were randomised by a researcher after enrolment and upon completion of baseline assessments. The randomisation sequence was generated by the web-based system *Sealed Envelope* (Sealed Envelope Ltd, London, United Kingdom), providing single allocation codes at each randomisation event and preventing researcher influence over group allocation. To maintain blinding, coded labels were used to inform treatment providers of assignments, the meaning of which was known only to providers. The key for decoding was kept by a colleague not directly involved in the research. The code was broken once an initial blinded analysis had been performed on the complete dataset, and results had been verified by the chief investigator and shared with the independent TSC/DMEC.

### 2.15. Blinding

Participants, outcome assessors, and data analysts were blinded to the nature of assigned interventions, except for assignment to the usual care arm (not blinded). Intervention providers were not blinded.

Blinding was attempted by using a high-similarity control intervention, following current guidance.^41^ Designed to replicate the NeuOst test intervention except for key components of interest, which were removed or altered, the control intervention aimed to produce comparable expectations, mirror context factors, and blind participants and research and administrative staff to group allocations. Providers were specifically trained about the importance of maintaining blinding—as were all other staff—and in the delivery of the control intervention (SDC, https://links.lww.com/PR9/A444).

### 2.16. Statistical methods and analysis

A detailed statistical analysis plan (SAP) was developed before enrolment, focusing on descriptive statistics for demographic variables and primary (feasibility) outcomes. Categorical data were analysed as frequencies, proportions, and percentages, while continuous data were presented as means and standard deviations (SD).

For feasibility outcomes, progression rules were prespecified (see section 3), including criteria to proceed, amend the study design, or consider aborting progression to a full-scale trial with the present methodology (SAP available at https://osf.io/jyftb).

Data analyses were performed in R (version 4.5.2) using RStudio (version 2026.01.0+392) for clinical measures and in STATA (version 13) or Excel for feasibility data.

The only feasibility outcome for which a (prespecified) between-group test was performed was blinding: Bang blinding index (BI) was calculated for the NeuOst and control intervention groups, respectively. Scaled to an interval of −1 to 1, a BI of 1 indicates complete lack of blinding in the control intervention group (correct allocation guessing), 0 is deemed consistent with perfect blinding or chance guessing, and −1 indicates opposite guessing (ie, control intervention participants believing to have received the NeuOst study treatment). The ratio of allocation guesses between groups was calculated as a standardised mean difference (Hedges g), re-coding BI directionality in 1 study arm as per Colagiuri et al.,^15^ and compared using a random effects model. In the absence of accepted best practice to compare blinding assessments between study arms,^41,95^ Bang blinding indices were also compared between groups using a χ^2^ test.^15^

As the study was not powered to test efficacy, no formal statistical hypothesis testing was conducted on clinical or safety outcomes. Instead, mean changes from baseline to follow-up for each outcome and group were calculated (SDC, https://links.lww.com/PR9/A444). Secondary descriptive analyses explored clinical changes in key candidates for future primary outcome measures. The pain severity and interference subscales of the Brief Pain Inventory (BPI) were chosen for these exploratory analyses because of the BPI's widespread use and validation in pain research,^28^ its sensitivity to change,^47^ and coherence with NeuOst intervention theory.^40^ Calculated using analysis of covariance adjusted for baseline values, we present BPI between-group differences with a range of confidence intervals (75% to 95%) in forest plots, with zero-difference and minimally important clinical differences (MCID) marked. In this study, a difference of 1.2 points was deemed clinically important for efficacy (NeuOst vs control intervention)^70,78^ and 2.0 for effectiveness comparisons (NeuOst vs usual care).^55^

All analyses were conducted using all available data as randomised. Deviating from our protocol, missing data were not imputed, recognising the feasibility-focused and descriptive nature of the clinical outcome data analysis in this study. Additional per-protocol sensitivity analyses included only data from participants who received the intervention they had been assigned to and who attended at least 3 of the 5 intervention sessions (except when in the usual care arm).

No interim analyses or stopping rules were planned or implemented.

### 2.17. Changes to trial protocol and protocol deviations

A protocol amendment specified that renal failure would only lead to ineligibility if requiring hospital-based dialysis; We had found that contemporary peritoneal dialysis is commonly performed at home and was unlikely to interfere with study participation.

Deviating from the original plan, resource limitations prevented us from video recording a subset of treatment sessions or monitoring fidelity with an independent assessor. Instead, we relied solely on provider report, who, at each session, marked all delivered treatment and control intervention elements in their treatment manuals. For 2 participants randomised to the NeuOst intervention, trial providers inadvertently used the control intervention manual to document intervention fidelity and care delivery. This appears to have resulted from a distribution error by clinic administrative staff. Although this affected the accuracy of fidelity documentation for these cases, our review indicates that the participants nevertheless received the intended NeuOst intervention. Unplanned sensitivity analyses were conducted to assess the impact of this error.

Data on healthcare utilisation and concomitant medication use were only collected at baseline to determine the feasibility of collecting such data. Follow-up data were not collected to reduce research burden. Physical foot exams were performed by providers at baseline for clinical orientation and patient communication only, and not to confirm eligibility as originally planned because, by then, participants had been enrolled and randomised.

### 2.18. Open science and data access

A full protocol, SAP, and the final, anonymised dataset are freely available on the Open Science Framework (OSF, https://osf.io/jyftb).

## 3. Results

### 3.1. Participant flow and CONSORT diagram

**Figure 2.**
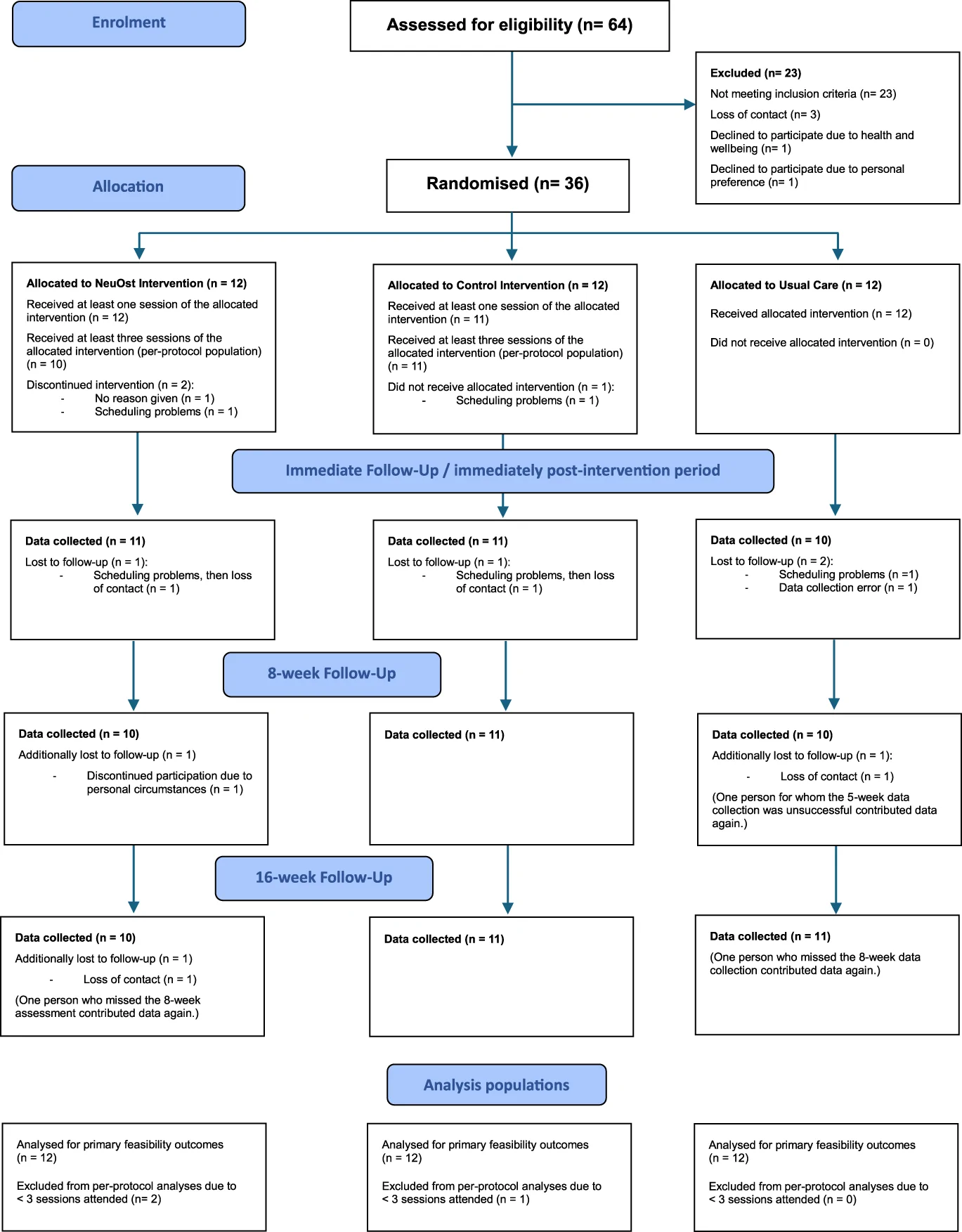
Participant flow diagram as per CONSORT guidance.

We enrolled the target of 36 participants. Twelve participants were randomised to each study group. Of those, 1 participant in the control intervention arm never attended a session, and 2 patients in the NeuOst arm discontinued treatment after 2 of the 5 sessions. Further detail on retention and adherence is provided in the feasibility analysis below and in Figure 2.

### 3.2. Sample characteristics at baseline

The recruited sample had diverse ethnic, linguistic, educational, and economic backgrounds. The average age was 64.2 years (SD 13.3), most participants were overweight or obese, and half lived with additional long-term conditions to diabetes and DPN (Table 1).

**Table 1:** Sample baseline characteristics.

Characteristic	Total sample	NeuOst	Control intervention	Usual care
N	36	12	12	12
Sex* (n, %)				
F	20 (56%)	9 (75%)	7 (58%)	4 (33%)
M	16 (44%)	3 (25%)	5 (42%)	8 (67%)
Age (mean, SD)	64.2 (13.3)	59.8 (16.3)	65.8 (12.5)	66.9 (10.6)
Ethnicity (n, %)				
Asian/Asian British	6 (17%)	3 (25%)	2 (17%)	1 (8%)
Black, Black British	14 (39%)	4 (33%)	6 (50%)	4 (33%)
Mixed or multiple ethnicities	1 (3%)	0 (0%)	0 (0%)	1 (8%)
White English or Welsh	15 (42%)	5 (42%)	4 (33%)	6 (50%)
Main language (n, %)				
English	31 (86%)	10 (83%)	10 (83%)	11 (92%)
Not English	5 (14%)	2 (17%)	2 (17%)	1 (8%)
Highest professional qualification (n, %)				
None	3 (8%)	1 (8%)	1 (8%)	1 (8%)
Apprenticeship	5 (14%)	2 (17%)	1 (8%)	2 (17%)
General Certificate of Secondary Education (GCSE) or comparable intermediate secondary school certificate	10 (28%)	5 (42%)	1 (8%)	4 (33%)
University degree	18 (50%)	4 (33%)	9 (75%)	5 (42%)
Work status†, (n, %)				
Student	0 (0%)	0 (0%)	0 (0%)	0 (0%)
Working as employee	8 (22%)	4 (33%)	2 (17%)	2 (17%)
Self-employed	7 (19%)	1 (8%)	4 (33%)	2 (17%)
Volunteering	1 (3%)	1 (8%)	0 (0%)	0 (0%)
Looking after home or family	9 (25%)	3 (25%)	4 (33%)	2 (17%)
Unemployed	1 (3%)	1 (8%)	0 (0%)	0 (0%)
Long-term sick or disabled	4 (12%)	1 (8%)	1 (8%)	2 (17%)
Retired	21 (63%)	6 (50%)	7 (58%)	8 (67%)
Disposable household income p.a. (n, %)				
<19,000	13 (36%)	2 (17%)	3 (25%)	8 (67%)
19,000–27,000	5 (14%)	2 (17%)	3 (25%)	0 (0%)
27,000–36,000	6 (17%)	3 (25%)	2 (17%)	1 (8%)
36,000–52,000	6 (17%)	1 (8%)	3 (25%)	2 (17%)
>52,000	2 (6%)	1 (8%)	0 (0%)	1 (8%)
Prefer not to say	4 (11%)	3 (25%)	1 (8%)	0 (0%)
Medical history (n, %)				
Living with another long-term condition (not diabetes or DPN)	18 (50%)	8 (42%)	5 (42%)	8 (42%)
Chemotherapy (history or present)‡	2 (6%)	0 (0%)	2 (18%)	0 (0%)
Alcohol problems (history or present)‡	2 (6%)	0 (0%)	1 (9%)	1 (9%)
BMI‡				
BMI, mean (SD)	29.5 (5.2)	31.6 (6.2)	27.9 (4.8)	29.1 (4.2)
Category: normal (n, %)	4 (11%)	0 (0%)	2 (17%)	2 (17%)
Category: overweight (n, %)	16 (44%)	7 (58%)	5 (42%)	4 (33%)
Category: obese (n, %)	16 (44%)	5 (42%)	5 (42%)	6 (50%)
MNSI (questionnaire part)				
Total score (mean, SD)	5.9 (2.1)	6.4 (2.4)	5.5 (2.2)	5.8 (1.9)
DN-4 (interview part)				
Total score (mean, SD)	4.8 (1.6)	4.9 (1.7)	4.9 (1.6)	4.4 (1.7)

Note that due to rounding, percentages may not add up exactly to 100%.

* All identifying with corresponding gender.

† Multiple responses possible.

‡ These data were not collected for the first 3 enrolled participants. Corresponding sample size is n = 33 (total), n = 11 per group.

BMI, body mass index; DN-4, Douleur Neuropathique 4; DPN, diabetic peripheral neuropathy; MNSI, Michigan Neuropathy Screening Instrument.

### 3.3. Provider characteristics

Six providers treated at least 1 patient as part of the trial. Providers were UK-registered osteopaths; 3 women and 3 men. At the start of the trial, they had spent 1 to 7 years in clinical practice (median 1.5 years' experience).

### 3.4. Primary analysis: feasibility and acceptability

Most prespecified criteria for progression were met, except for recruitment, where a modified strategy would be needed in a full-scale trial (Table 2). One analysis of blinding also did not meet all predefined success criteria, although this indicated unbalanced blinding between groups as opposed to a lack of blinding in any of the 2 relevant study arms.

**Table 2:** Results and progression criteria for feasibility outcomes.

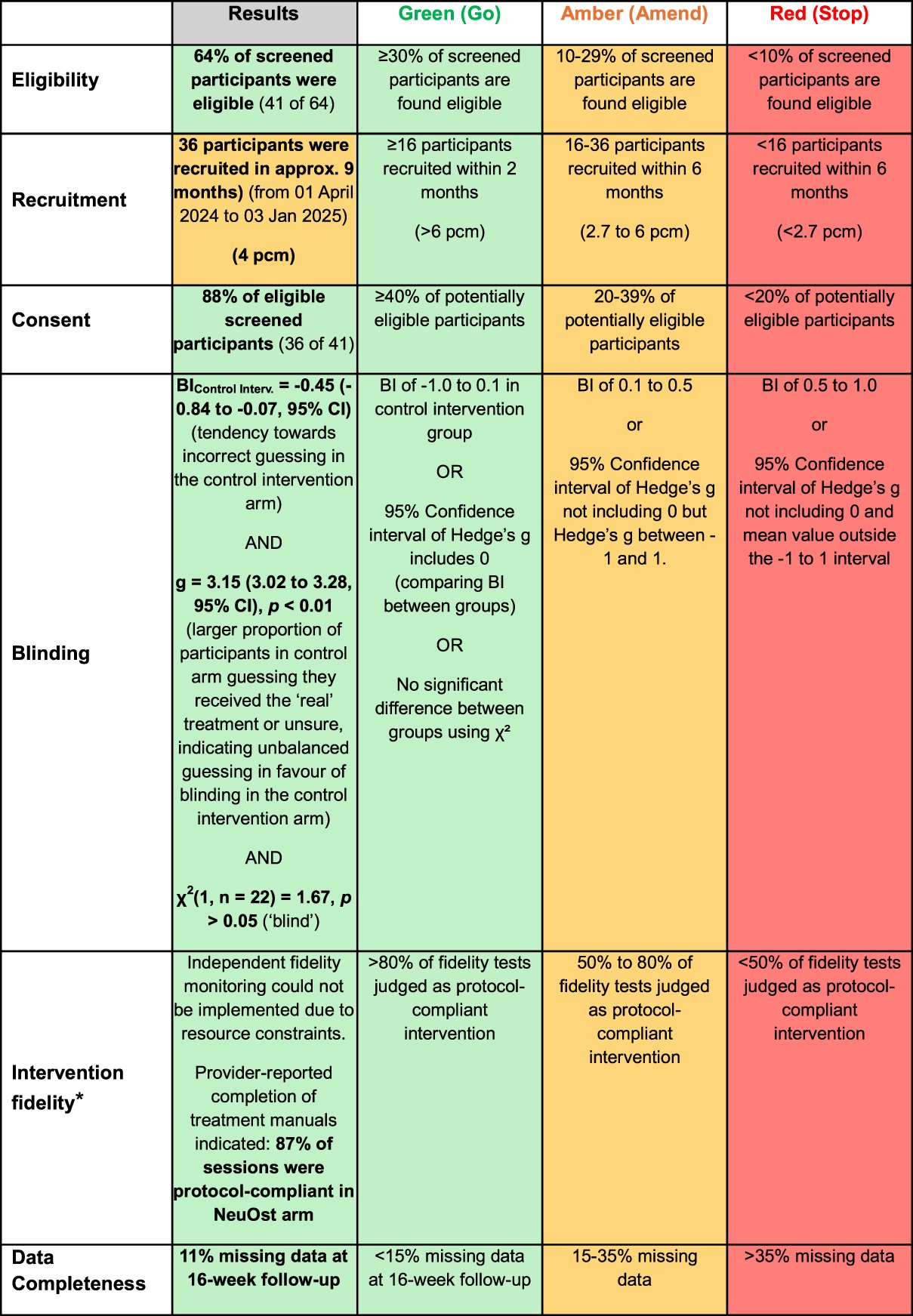
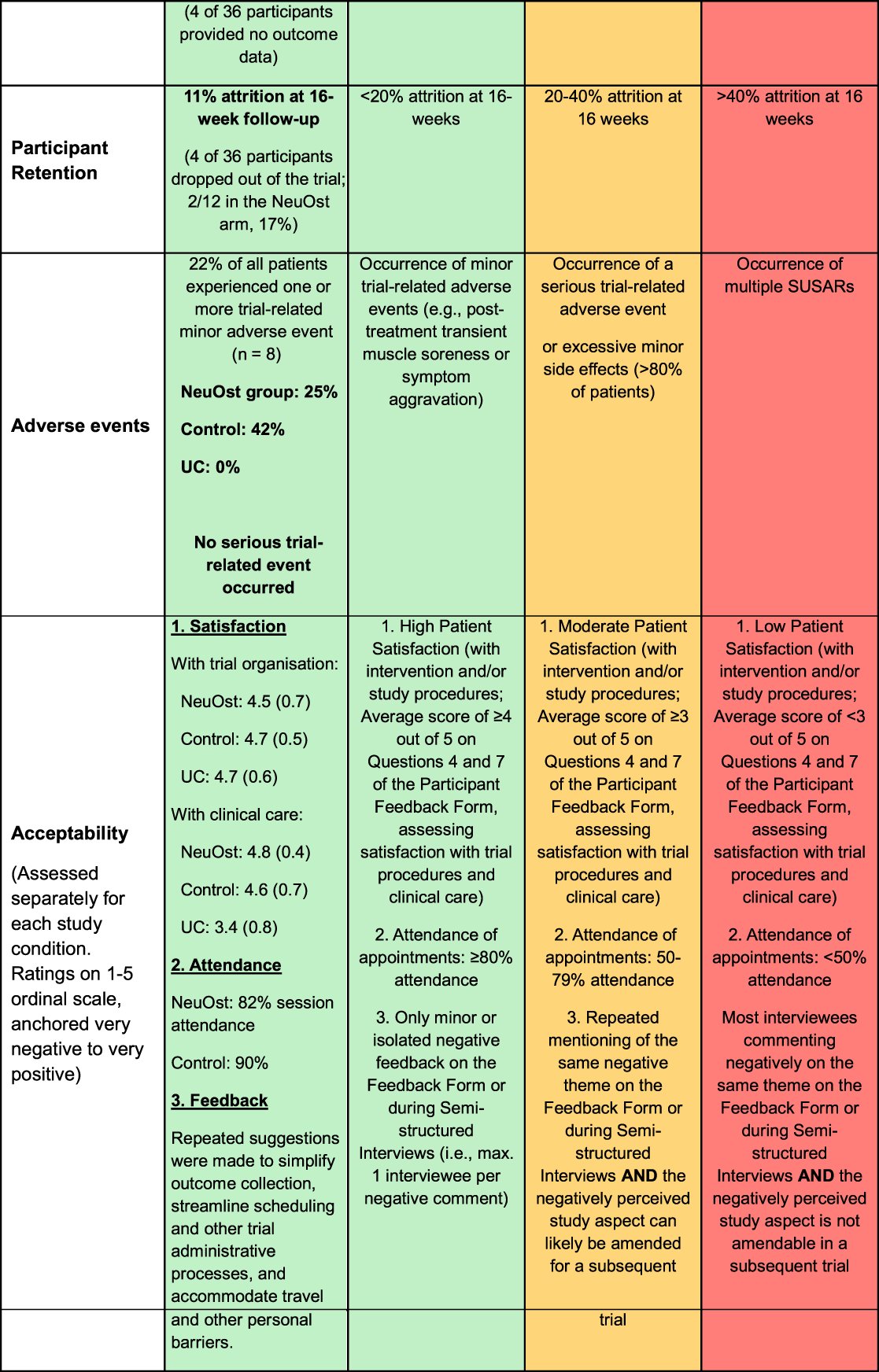

Note that the blinding index is scaled to an interval of −1 to 1; 1 being complete lack of blinding in the control intervention group (correct allocation guessing), 0 being consistent with perfect blinding or chance guessing, and −1 indicating opposite guessing (ie, control intervention participants believing to have received the test treatment).

*All results in this table are based on the intention-to-treat population (participants in groups as randomised); An error in the distribution of study materials meant that treatment documentation was erroneous for two patients in the NeuOst arm (7 sessions in total), reducing intervention fidelity scores somewhat in this study arm. A sensitivity analysis is presented in the manuscript for the relevant feasibility measure “fidelity.” This sensitivity analysis indicated no major influence of this error on feasibility findings.

BI, Bang blinding index; UC, usual care. SUSAR, Suspected Unexpected Serious Adverse Reaction.

#### 3.4.1. Recruitment, eligibility, and consent rates

The target number of 36 participants was recruited in approx. 9 months, from April 1, 2024 to January 3, 2025, meaning an average of 4 participants were recruited per calendar month.

Over this period, contact was made with 82 individuals, of whom 62 (76%) were formally screened, with 41 (66%) of those screened deemed eligible. Of the 41 participants screened as eligible, 88% (36) consented to trial participation (Table 2; Fig. 2).

Ineligibility was largely due to the absence of neuropathic pain (20%) or DPN (17%) as per DN4 and MNSI questionnaire assessments, and no or unclear diabetes (15%), followed by ineligible comorbidities, lack of foot pain, and unstable medication regimens.

Most patients contacted the study team after learning about the study through a local diabetes clinic network (n = 16) or through word of mouth (n = 10). Flyers accounted for 24 contacts across podiatry clinics, private clinics, the HSU-UCO trial site, other NHS sites, public spaces, and unspecified locations. Additional sources included emails from clinics/organisations (n = 10), newspapers (n = 3), social media (n = 2), the study newsletter (n = 1), and onward referrals from another study (n = 5).

#### 3.4.2. Treatment completion and study retention rates

Participants attended an average of 86% of treatment sessions (across NeuOst and control intervention groups) (103/120). Participants in the NeuOst group attended 82% of their sessions (49/60, or 4.1 of 5 sessions per participant, Table 2)—with 2 participants only attending 2 sessions each. In the control intervention, attendance was 90% (54/60, or 4.5 sessions per participant)—with 1 participant not attending any sessions.

Four participants dropped out of the trial (11% attrition), ie, also stopped attending data collection calls: 3 due to unknown reasons and lost contact (1 in each study arm), and 1 due to personal circumstances interfering with trial participation (in the NeuOst arm) (Fig. 2). The attrition rate in the NeuOst arm was thus 17% (Table 2).

#### 3.4.3. Data completeness

Participants who attended assessment calls always responded to all required questions. Missing data were thus calculated simply as the proportion of missed assessment calls, rather than data points. Missed data collections ranged from 8% to 17% per group and follow-up timepoint, with no notable differences between groups or time points (SDC Table S1, https://links.lww.com/PR9/A444). The total rate of missing data across all groups and the 3 follow-up time points was 12%.

#### 3.4.4. Acceptability and satisfaction

At the final point of follow-up, participants were asked several feedback questions about their experience in the study. Overall, satisfaction with trial organisation and clinical care was high across groups. Almost all participants indicated they would participate in a larger study and would recommend to others to participate in a similar study (Table 3).

**Table 3:** Acceptability and satisfaction feedback by study participants.

Items	NeuOst (n = 10)	Control (n = 11)	Usual care (n = 11)	Total (n = 32)
Clarity of instructions before trial start	4.5 (0.7)	4.2 (1.6)	4.5 (0.7)	4.4 (1.1)
Ease of completing trial procedures during trial (eg, attending visits and assessments)	4.4 (1.0)	4.3 (1.0)	4.3 (0.5)	4.3 (0.8)
Satisfaction with trial organisation	4.5 (0.7)	4.7 (0.5)	4.7 (0.6)	4.7 (0.6)
Ease of completing home exercises	4.3 (0.5)	4.5 (0.7)	N/A	4.0 (0.8)
Satisfaction with clinical care	4.8 (0.4)	4.6 (0.7)	3.4 (0.8)	4.3 (0.9)
Would participate in larger study	n = 10 (100%)	n = 10 (91%)	n = 11 (100%)	n = 31 (97%)
Would recommend to others to participate in a similar study	n = 10 (100%)	n = 11 (100%)	n = 10 (91%)	n = 31 (97%)

Collected at the final follow-up timepoint (16 weeks). Ratings on a 1 to 5 ordinal scale, coded so that higher scores indicate higher agreement or satisfaction. Anchors were “very negative,” “negative,” “neutral,” “positive,” and “very positive” or terms to that effect (“easy,” “satisfied,” etc). Data presented as mean and SD, unless otherwise indicated.

Responses to open-ended feedback questions indicated that participants generally found the trial acceptable and the interventions beneficial (SDC Table S2, https://links.lww.com/PR9/A444). Participants also valued personal contact and relationships with providers, manual therapy, and scheduling flexibility. Feedback highlighted difficulties with outcome questionnaires, occasional uncertainties about procedures, frustration with not receiving trial-related care in the usual care group, and practical participation barriers. Suggestions were made for improved communication, simplified outcome assessments, and longer or more frequent intervention sessions.

#### 3.4.5. Interventionist fidelity in treatment delivery

During 87% of intervention sessions in the NeuOst arm, providers delivered over 80% of mandatory treatment protocol items, as measured by a provider-completed treatment checklist. Errors in distributing treatment documentation materials meant that treatments were documented with the wrong case report form for 2 participants in the NeuOst arm (affecting a total of 7 sessions). When excluding these sessions from the treatment fidelity analysis, the proportion of high-fidelity NeuOst sessions was 95%.

The treatment handbook also included optional protocol items, of which an average range of 4% to 9% were delivered per session. Here, the most delivered optional component in the NeuOst arm was making modifications to the exercise programme to meet patient needs. Detailed fidelity data are provided in the SDC (Table S3, https://links.lww.com/PR9/A444).

#### 3.4.6. Treatment credibility, expectations, and blinding assessments

Results of treatment expectation and credibility assessments are reported in Table 4. Although not tested statistically, no marked differences between the groups were noted.

**Table 4:** Treatment expectations and intervention credibility results.

Outcome measure	Baseline				Visit 2			
CEQ	Total (n = 34)	NeuOst (n = 12)	Control (n = 11)	UC (n = 11)	Total (n = 23)	NeuOst (n = 12)	Control (n = 11)	UC
Credibility	7.6 (1.1)	7.8 (1.0)	7.4 (1.3)	7.7 (1.1)	6.2 (1.3)	6.3 (1.6)	6 (0.9)	N/A
Expectancy	6.4 (1.7)	5.7 (2.1)	6.6 (1.5)	7.0 (1.1)	5.4 (1.3)	5.1 (1.6)	5.8 (0.9)	N/A
SETS	Total (n = 36)	NeuOst (n = 12)	Control (n = 12)	UC (n = 12)	Total (n = 23)	NeuOst (n = 12)	Control (n = 11)	UC
Positive expectations	4.5 (1.3)	3.8 (1.1)	4.5 (1.6)	5.1 (1.0)	4.3 (1.5)	3.9 (1.7)	4.8 (1.1)	N/A
Negative expectations	1.4 (0.7)	1.4 (0.7)	1.4 (0.9)	1.5 (0.9)	2.0 (1.8)	2.1 (2.0)	2.0 (1.5)	N/A
Naïve (to “osteopathy”)	n = 28 (78%)	n = 9 (75%)	n = 12 (100%)	n = 7 (56%)	N/A	N/A	N/A	N/A

Assessed using Borkovec Credibility and Expectancy Questionnaire (CEQ: presented as a 1–9 scale) and the Stanford Expectations of Treatment Scale (SETS: 1–7 scale) at baseline and before treatment visit 2. Data presented as mean and standard deviation, unless otherwise indicated.

UC, usual care.

##### 3.4.6.1. Blinding group guesses at immediate follow-up

Immediately after the intervention period, participants were asked to guess which of the 3 study groups they were in. Their guesses are shown in Table 5. Although an equal number of participants in the NeuOst and in the control intervention arm believed to have received the (NeuOst) study treatment, more people in the NeuOst arm believed to have had the control intervention, and more control intervention participants were unsure.

**Table 5:** Results of blinding guesses.

Intention to Treat (ITT) population	NeuOst (allocation) (n = 11)	Control intervention (allocation) (n = 11)	UC (allocation) (n = 10)
NeuOst (guess)	6	6	0
Control intervention (guess)	3	1	0
Don't know (guess)	2	4	0
UC (guess)	0	0	10

Presented per group and assessed immediately after the end of the treatment period (or at week 5 from randomisation in the usual care arm).

UC, usual care.

###### 3.4.6.1.1. Bang blinding index

Group guesses were compared with BI. Bang blinding index in the NeuOst arm was 0.27 (−0.24 to 0.78, 95% confidence interval, CI; n = 11), indicating a tendency towards correct guessing in this arm, but with random guessing (i.e., BI = 0) well within the 95% CI. Bang blinding index in the control intervention group was −0.45 (−0.84 to −0.07, 95% CI; n = 11), meaning patients tended towards guessing their allocation incorrectly in this group, believing they received the “real” intervention. The confidence interval does not include chance guessing.

###### 3.4.6.1.2. Hedges g of Bang blinding indices

Hedges g was used to quantify the differences in blinding guesses between NeuOst and control intervention groups. It was g = 3.15 (3.02–3.28, 95% CI), *P* < 0.01, indicating that a larger proportion of patients in the control intervention arm thought they received active treatment or did not know which intervention they received than in the NeuOst arm (Table 5).

###### 3.4.6.1.3. Chi-squared test comparing group guesses

The χ^2^ test, used to compare raw guessing data rather than blinding indices, indicated no significant difference in allocation guessing between the NeuOst and control intervention groups (χ^2^[2, n = 22] = 1.67, *P* = 0.37).

###### 3.4.6.1.4. Reasons for group guesses

Participants were asked to justify their group guesses. The reasons for guessing that a “real” treatment had been received—irrespective of the actual intervention received—related to (1) the perceived authenticity of the experience, (2) clinical effects or the care being perceived as overall helpful, and (3) participants recognising intervention components as familiar (SDC, Table S4, https://links.lww.com/PR9/A444).

Some reasons given for group guesses were difficult to reconcile with the direction of the guess. For example, 1 NeuOst-arm participant guessed they were in the control intervention group because the sessions met their expected level of massage and discussion, while another felt the practitioner's activities were familiar and unsurprising. The use of familiar components in NeuOst may have reduced novelty and thus contributed to successful blinding. Another participant, also guessing control, perceived the sessions as largely conversational and monitoring-focused rather than a “real” treatment with “actual discussions or prescription of medications,” highlighting how violated/unrealistic expectations may produce incorrect guesses.

Control group participants who believed they received the NeuOst treatment cited the intensity and thoroughness of the sessions, hands-on techniques, psychological support, exercises, and improvements in pain and mobility as reasons for their belief. Psychological support, clinician attention, and diagnostic conversations also contributed to this perception.

#### 3.4.7. Adverse events

Three patients (25%) in the NeuOst intervention arm experienced at least 1 *trial-related* adverse response, with 5 (42%) in the control intervention and none (0%) in the usual care arm. All trial-related events were mild, except 1 post-treatment leg pain episode in the control intervention arm rated as 'severe'. No serious trial-related reactions occurred (Table 6).

**Table 6:** Adverse events in NeuOst feasibility trial.

	NeuOst (n = 12)	Control intervention (n = 12)	UC (n = 12)
Adverse event			
Any event	12	22	4
Unique patients with at least 1 AE	6 (50%)	11 (92%)	3 (25%)
Serious adverse events	1	1	0
Relationship to study interventions			
Probably/definitely related	5	6	—
Unique patients with trial-related events	3 (25%)	5 (42%)	0 (0%)
Serious trial-related adverse reaction	—	—	—
Adverse event categorisation (MedDRA system organ class and MedDRA preferred term)	Total (related*)	Total (related*)	Total (related*)
Musculoskeletal and connective tissue disorders			
Pain in lower extremity (increased or new)	5 (3*)	4 (1*)	—
Gastrointestinal disorders			
Abdominal pain	—	1	—
General disorders and administration site conditions			
Pain (other anatomical location)	1 (1*)	3 (2*)	—
Nervous system disorders			
Paraesthesia (in lower extremity; increased or new)	1 (1*)	3 (3*)	1
Memory impairment	—	1	—
Restless leg syndrome (new diagnosis)	1	—	—
Injury, poisoning, and procedural complications			
Fall, no fracture	1	0	1
Fall, with fracture	—	1	—
Infections and infestations			
Upper respiratory tract infection	1	3	1
Infection (other)	1	1	1
Immune system disorders			
Lymphadenopathy (lymph node swelling)	—	1	—
Metabolism and nutrition disorders			
Hypoglycaemia (hypoglycaemic episode)	1	3	—
Social circumstances			
Drug dispensing issue (inability to obtain prescribed medications)	—	1	—
Severity			
Minor	8 (5*)	14 (5*)	1
Moderate	3	5	2
Severe	1	3 (1*)	1
Life-threatening	—	—	—
Fatal	—	—	—

Presented as cumulative individual events per study arm over the entire duration of the trial.

* Adverse events rated as “probably” or “definitely related” to the study interventions.

AE, adverse event; UC, usual care.

### 3.5. Secondary outcomes (clinical measures)

Detailed descriptive data for the below clinical outcome measures are presented in the SDC, https://links.lww.com/PR9/A444.

#### 3.5.1. Pain, neuropathy, quality of life, and disability measures

We successfully collected data for the BPI, the number of days with bothersome and intolerable DPN symptoms in the past week, NeuroQoL, the DPNPI, and the FES-I. Descriptive inspection of scores across follow-up timepoints suggested that values were often lower than baseline across all these measures in both the NeuOst and the control intervention group—a pattern not observed in the UC group. This included suggested benefits in the NeuroQoL and DPNPI domains relating to symptom severity and frequency, mobility/physical function, sleep, emotional distress, daily activities, and social function (SDC, Table S5, https://links.lww.com/PR9/A444).

**Figure 3.**
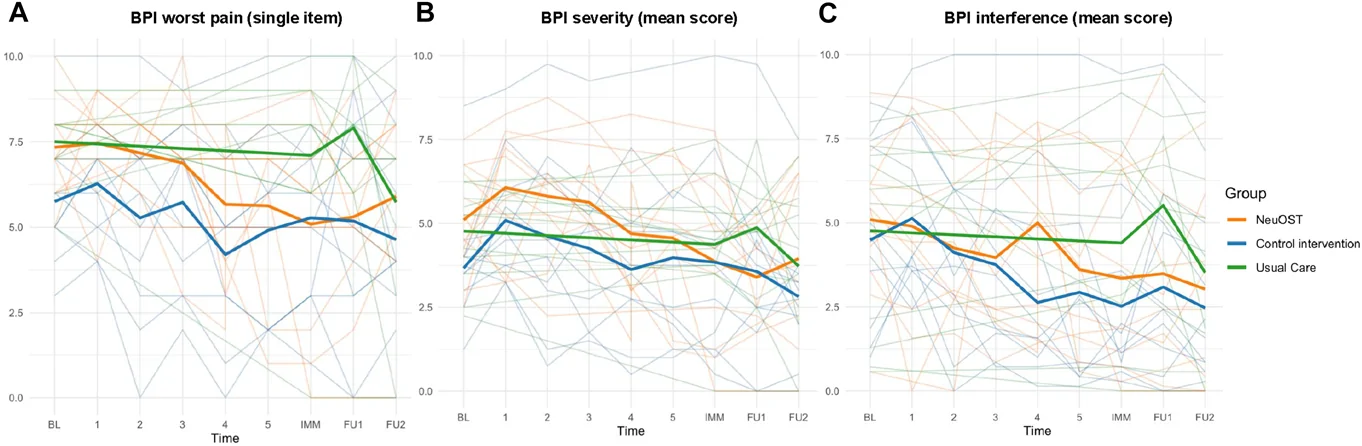
Spaghetti plots of Brief Pain Inventory data. Panel (A): worst pain in past week; Panel (B): Pain severity domain. This BPI domain combines ratings for worst, least, average, and current pain in the past week; Panel (C): Pain interference domain. This BPI domain combines ratings for pain interference with general activity, mood, walking ability, normal work, social relations, sleep, and enjoyment of life in the past week. Data for all items were collected at baseline (BL), immediately after the end of the treatment period (or 5 weeks from randomisation, IMM), at 8 weeks post-randomisation (FU1) and at 16 weeks (FU2). In addition, data were collected before clinical visits (1–5) for participants in the NeuOst and control intervention groups. BPI, Brief Pain Inventory.

Additional descriptive analyses were performed for the Brief Pain Inventory's severity and interference domains and for the item “worst pain in the past week.” The spaghetti plots in Figure 3 provide a similar picture of nondifferentiation between NeuOst and the control intervention, but separation from the symptom development in the usual care arm, especially at the first 2 follow-up timepoints (immediately after the end of the treatment period and 3 weeks later).

**Figure 4.**
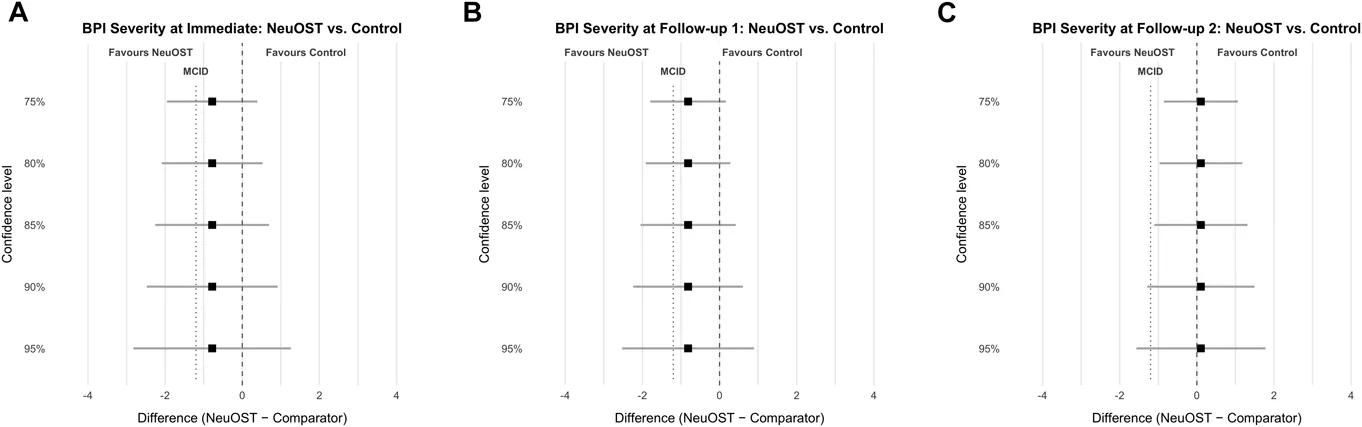
Brief Pain Inventory, pain severity subscale. Forest plots illustrating changes from baseline in NeuOst versus control intervention arms. Results of a baseline-adjusted analysis of variance in between-group differences are presented with 75% to 95% confidence intervals. The Minimal Clinically Important Difference (MCID) of 1.2 points (dotted line) and the line of no difference are marked (dashed line). Panels illustrate the immediate timepoint of follow-up (panel A), 8-week follow-up (panel B), and 16-week follow-up (panel C).

**Figure 5.**
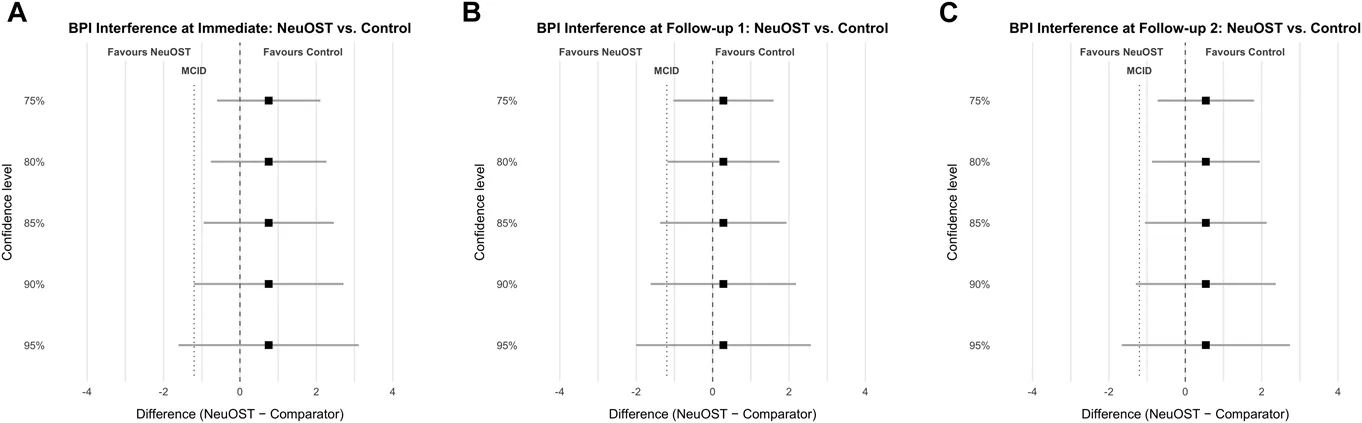
Brief Pain Inventory, pain interference subscale. Forest plots illustrating changes from baseline in NeuOst versus control intervention arms. Results of a baseline-adjusted analysis of variance in between-group differences are presented with 75% to 95% confidence intervals. The Minimal Clinically Important Difference (MCID) of 1.2 points (dotted line) and the line of no difference are marked (dashed line). Panels illustrate the immediate timepoint of follow-up (panel A), 8-week follow-up (panel B), and 16-week follow-up (panel C).

When comparing changes in BPI measures descriptively between NeuOst and the control intervention using ANCOVA-based forest plots, the 80% confidence intervals of group differences included the zero-line of no difference in all cases (Figs. 4 and 5). However, for pain severity, the MCID of 1.2 points in favour of NeuOst was also encompassed by the confidence intervals at immediate and medium-term follow-up timepoints but not long-term (16 weeks) (Fig. 4). Mean differences for pain interference favoured the control intervention at all timepoints, only encompassing the MCID in favour of NeuOst at 8 weeks and always crossing the zero-difference line (Fig. 5).

**Figure 6.**
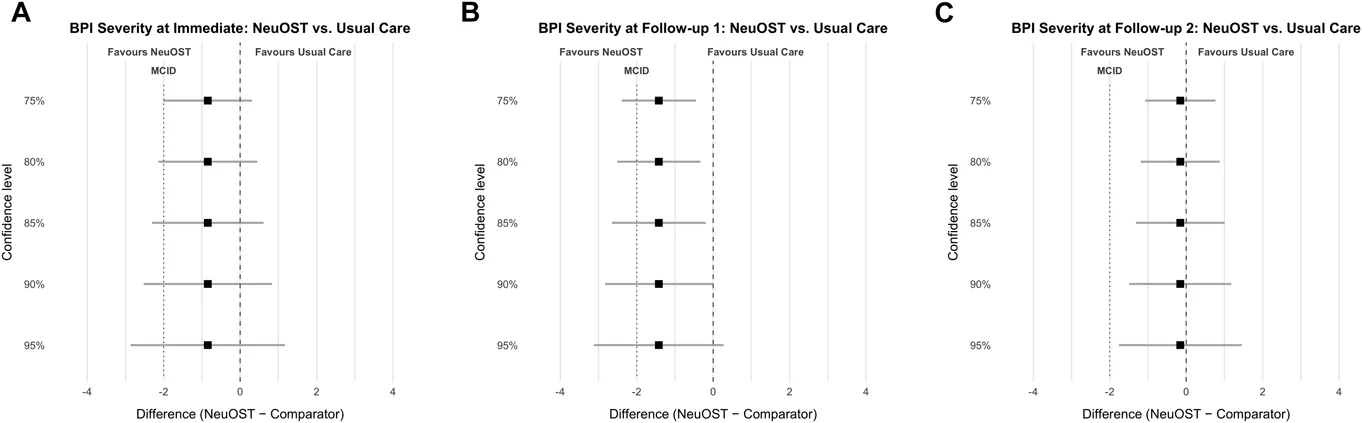
Brief Pain Inventory, pain severity subscale. Forest plots illustrating changes from baseline in NeuOst versus usual care arms. Results of a baseline-adjusted analysis of variance in between-group differences are presented with 75% to 95% confidence intervals. The Minimal Clinically Important Difference (MCID) of 2 points (dotted line) and the line of no difference are marked (dashed line). Panels illustrate the immediate timepoint of follow-up (panel A), 8-week follow-up (panel B), and 16-week follow-up (panel C).

**Figure 7.**
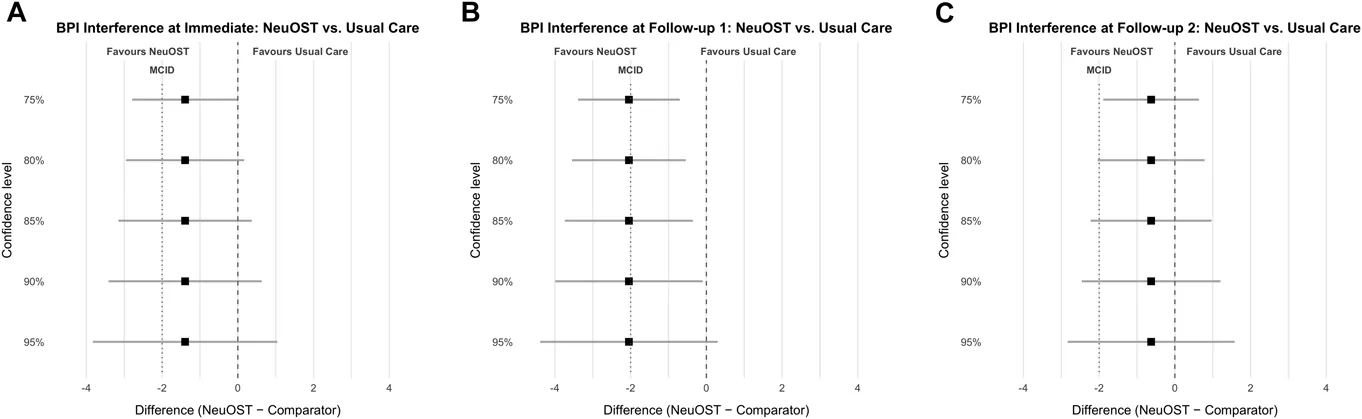
Brief Pain Inventory, pain interference subscale. Forest plots illustrating changes from baseline in NeuOst versus usual care arms. Results of a baseline-adjusted analysis of variance in between-group differences are presented with 75% to 95% confidence intervals. The Minimal Clinically Important Difference (MCID) of 2 points (dotted line) and the line of no difference are marked (dashed line). Panels illustrate the immediate timepoint of follow-up (panel A), 8-week follow-up (panel B), and 16-week follow-up (panel C).

Compared with usual care, these 80% confidence intervals included the 2-point MCID at all instances in favour of NeuOst, except for pain severity at the 16-week follow-up. At the 8-week follow-up, when between-group differences were most marked, the zero-difference line was outside the 80% CIs, and the mean difference was close to the MCID (Figs. 6 and 7).

#### 3.5.2. Patient global impression of change

**Figure 8.**
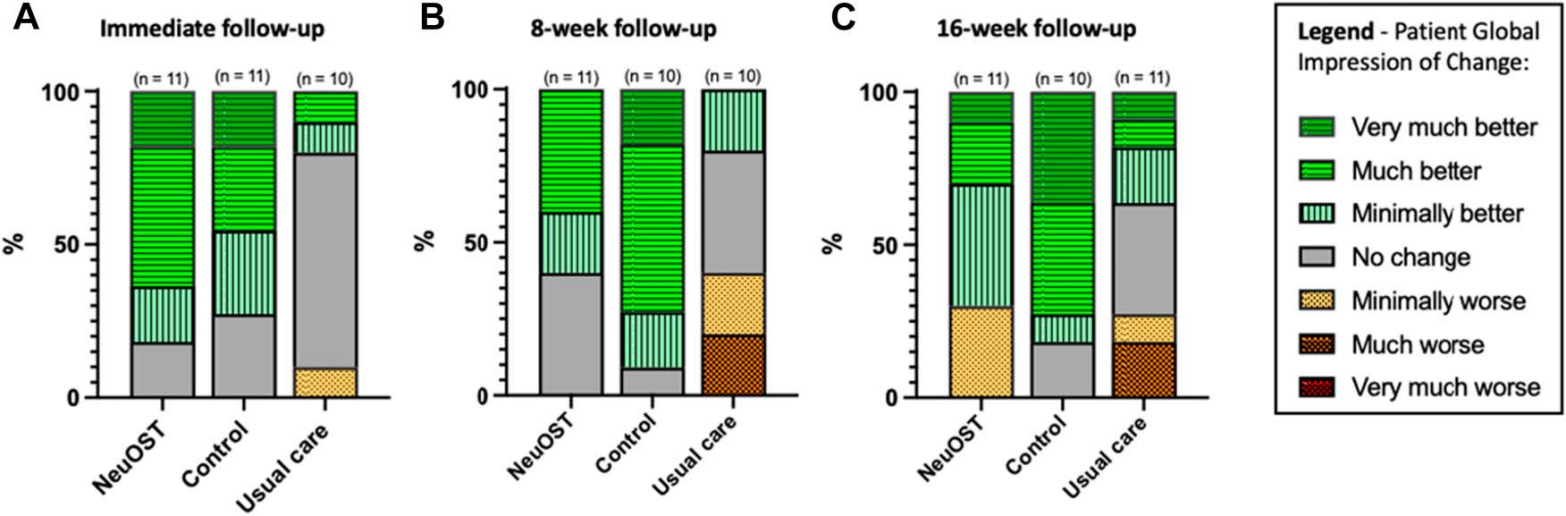
Participant global impression of change. Results presented as percentage of participants per group and timepoint (Panel A: At immediate follow-up, B: 8 weeks after randomisation, C: At 16 weeks.

Across all 3 follow-up timepoints, 60% to 90% of participants in the NeuOst and the control intervention groups reported their overall health status to be minimally, much, or very much improved compared with the beginning of this study. In the usual care arm, only 20% to 36% of participants did so, and most (57%–80%) reported no change or some worsening (Fig. 8). Numerical data are provided in the SDC (Table S6, https://links.lww.com/PR9/A444).

#### 3.5.3. Healthcare utilisation and medication use

Healthcare utilisation data were only collected at baseline. Patients reported having used an average of 1.6 healthcare services in the preceding 4 weeks (SD 1.3) (SDC Table S7, https://links.lww.com/PR9/A444). Across all 36 patients, this included 12 patients consulting with GPs, 14 seeing a diabetes nurse, and 13 attending specialist services. Only 5 (14%) consulted no healthcare services at all in the past month.

Medication use was collected through patient self-report. Given the time requirements and challenges involved in verbally collecting such data through phone or video calls—especially from patients with long medication lists—the research team resorted to asking for medication lists to be sent through email. This resulted in considerable additional administrative burden, suggesting a different approach to data collection would be needed in a full-scale trial. Data are not presented here.

#### 3.5.4. Physical activity levels and exercise adherence

**Figure 9.**
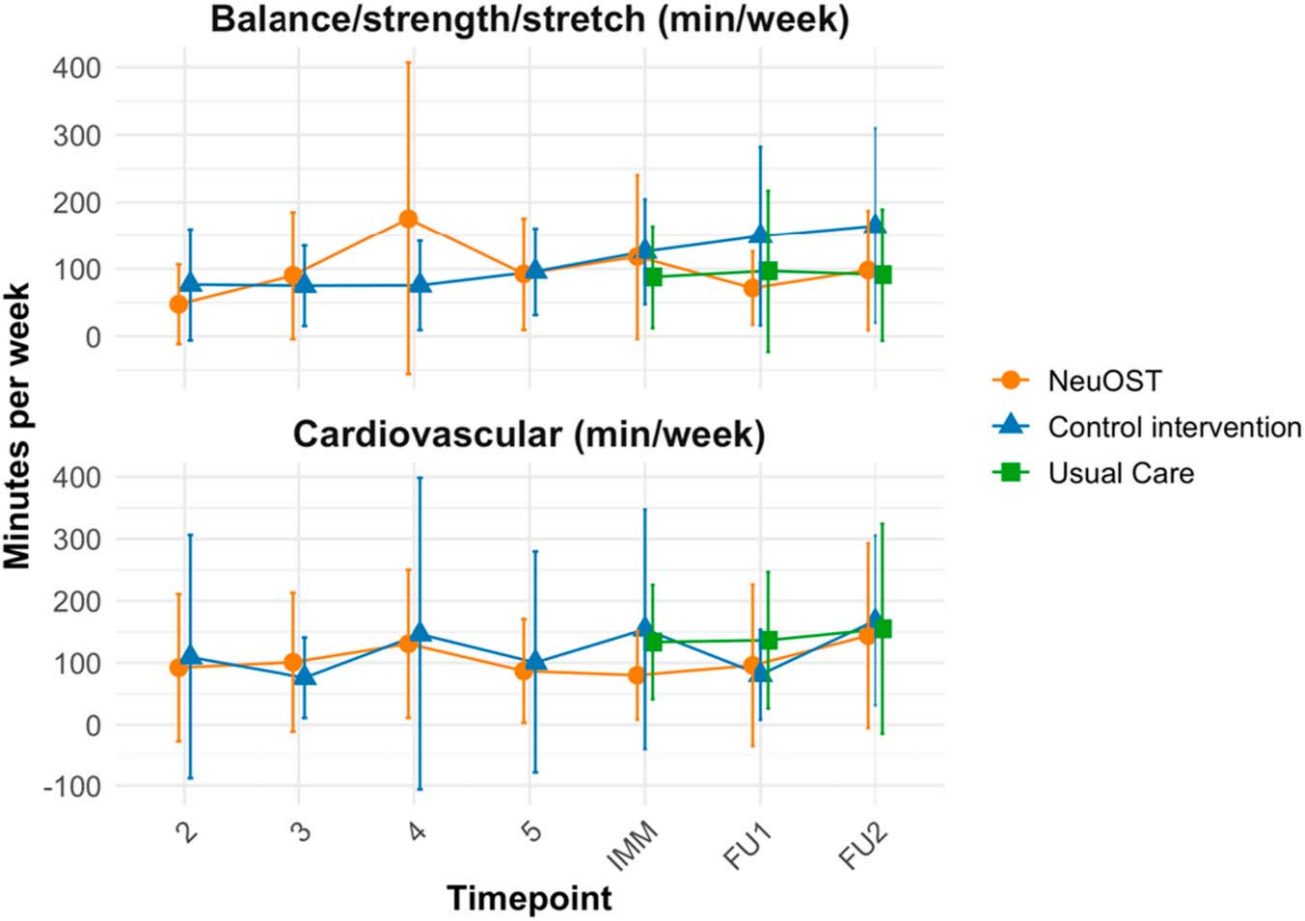
Self-reported physical activity levels. Presented as means and standard deviations for each group and timepoint of data collection. Data were collected before clinical visits 2 to 5 for participants in the NeuOst and control intervention groups, and immediately after the end of the treatment period (or 5 weeks from randomisation, immediate follow-up, IMM), at 8 weeks post-randomisation (FU1) and at 16 weeks (FU2) for all study arms.

Figure 9 describes participant-reported time spent doing either any strength, stretch, or stability exercises, or any cardiovascular activity. Spaghetti plots and data tables are provided in the SDC (Tables S8 and S9; Figures S1 and S2, https://links.lww.com/PR9/A444). Although not tested formally, the data indicate no major changes in activity levels either within or between groups. Data variance around means was considerable.

Data collection proved difficult for several reasons: First, we used a self-designed questionnaire asking for exercise activities “since the last session” rather than in the past week. Although data collectors soon switched to enquiring about activities in the past week and we retrospectively corrected the data, this may have led to errors. Secondly, due to the wording of the questionnaire, no data were collected before the first session, thus failing to establish a baseline.

### 3.6. Subgroup and sensitivity analyses

Sensitivity analyses of the BPI data exploration were performed with a per-protocol sample, only including participants who attended at least 3 NeuOst or control intervention sessions. Although not presented here in detail, results did not differ notably. No further subgrouping was deemed relevant or feasible, also due to small sample sizes.

## 4. Discussion

This study demonstrated the feasible delivery and high acceptability of a novel intervention for people with painful diabetic neuropathy, combining manual therapy techniques, staged exercises, and psychologically informed conversations, alongside assessments, education, and complication screening by Allied Health professionals.^40^ The diverse pDPN sample was highly satisfied and adherent. The three-armed RCT was largely feasible, comparing the NeuOst intervention to a blinded high-similarity control intervention and nonblinded usual care. Key feasibility markers were met, although recruitment procedures need modification for a larger trial. Mild adverse responses were common, but no serious adverse reactions occurred.

### 4.1. Feasibility measures

Methodologically, the study design appeared promising, meeting criteria for eligibility, consent, retention, blinding, fidelity, data completeness, and study acceptability. Recruitment was challenging using mainly public advertising, although advertising in NHS spaces helped. Future trials should recruit directly through diabetes services and existing patient lists. Participant feedback and the long duration of outcome collection calls indicated a need to simplify data collection—using less burdensome measures, especially for disability and quality of life, and obtaining healthcare utilisation data from electronic records. Better methods are also needed for exercise monitoring, and more resources for independent fidelity monitoring.

The control intervention was designed to mimic NeuOst and blind participants and research staff to allocation, building on methodological guidance.^41^ Close collaboration with trial providers and patient partners was key to its success, fostering provider buy-in and confidence through co-design and training.^40^ Problems arose from the practical complexities of communicating using allocation codes and confusingly similar sets of treatment manuals. More piloting, closer supervision, and limited involvement of an unblinded researcher may have mitigated these challenges. Clinical results point to another concern with high-similarity control interventions: Although not powered to detect clinical effects, outcome differences between the NeuOst and its control intervention were smaller than those compared with usual care—if present at all. Of note, expectation measures were comparable between all 3 groups and blinding was likely effective. This comparison of NeuOst to its high-similarity control intervention prompts reconsideration of the intervention theory: Matched components such as validation, education, touch, personalised care, and attention may play central roles for clinical effects.^39^ In a full trial, we will intensify key NeuOst intervention components. If designing an efficacy trial, we will ensure that theoretically active treatment components are distinctly absent from the control intervention—including through better fidelity monitoring or a stepped-wedge cluster design. Overall, the methodological success of a credible blinded control intervention brings practical and scientific challenges, but feasibility trials such as this one are crucial to identify and adapt to them.^41^ In a full trial, we will consider which comparator is most suitable to inform decision-making and best aligned with healthcare and research priorities.^12,38,41,42^

### 4.2. Clinical outcomes

Clinically, the study underscores the potential value of person-centred, multimodal nonpharmacological care for people with pDPN.^88^ Clinical changes were encouraging—particularly shortly after the intervention period and contrasted with usual care. However, variability was large, no difference was apparent compared with the high-similarity control intervention, and the small sample prevented firm conclusions.

Compared with usual care, NeuOst showed preliminary and exploratory signals of improvement across all collected outcomes, including symptom severity, interference with function and emotional wellbeing (BPI, NeuroQoL, and DPNPI), fear of falling (FES-I), and patients' global impression of change. Days with “bothersome” symptoms also potentially decreased in both intervention groups. This breadth of possible effects corresponds to NeuOst's intervention theory, which postulated multiple target mechanisms across psychological, behavioural, and physical domains.^40^ Theorised therapeutic outcomes were correspondingly broad and included quality of life, fear of falling, and healthcare utilisation. Pain and symptom severity were not key targets but may also have improved.

Selecting a suitable primary outcome measure for future trials remains challenging, given the feasibility trial results and the intervention's complexity. If focusing on QoL and disability, both the NeuroQoL^89^ and DPNPI^10^ appeared responsive to NeuOst in this preliminary trial. However, both were cumbersome to administer, and the NeuroQoL does not lend itself to calculating a total score. Other neuropathy-specific QoL measures may be preferable.^77^ The DPNPI does not reflect the multitude of sensory disturbances in DPN, being specific to neuropathic pain. The BPI is similarly limited. Although not tested here, generic, brief QoL measures, such as the Short Form 12 Health Survey (SF-12) or the EQ-5D-5L Health Questionnaire, may mitigate these concerns and can be used in neuropathic pain.^87^ We also trialled ultra-brief symptom “bothersomeness” and “tolerability” questions,^31,54^ which—while promising and aligned with the NeuOst intervention theory—need population-specific validation. Finally, the Patient’s Global Impression of Change scale appeared well suited. It was easy to administer, conceptually broad, seemingly differentiated between NeuOst and usual care, and has been shown to correlate with multiple relevant measures in a neuropathic pain population.^67^ A qualitative realist evaluation which accompanied our study, along with further stakeholder engagement, will inform the choice of a main trial's primary outcome. Assessing self-efficacy and physical parameters may shed further light on intervention mechanisms in future studies.

### 4.3. Intervention context

People experiencing neuropathic pain have insufficient pharmacological options^79^ and desire alternative approaches.^18,19,40^ The high satisfaction with clinical care in the NeuOst and control intervention arms may be influenced by the contrast between this intense, personalised care programme and the otherwise limited care provision in the United Kingdom.^19,27,32^ Potentially, patients' enthusiasm also translated into high adherence and compliance rates.

Practical lessons from this feasibility trial should be integrated with new evidence from the rapidly evolving field of nonpharmacological neuropathy care, notably exercise.^81^ Although studies remain small and methodologically heterogeneous, reviews suggest improved neuropathy symptoms and physical function, particularly from sensorimotor and endurance training. However, most trials provide exercise at higher intensities than this NeuOst study.^35,81,90,94^ Evidence for psychological interventions remains sparse and is dominated by mindfulness-based therapies, showing promise.^6^ Psychologically informed physical or multimodal interventions are absent from the peripheral neuropathy literature,^51^ except for NeuOst^40^ and rare programmes combining mindfulness or education with exercise.^66,84,92^ Here, lessons can be learnt from combined approaches in back pain and musculoskeletal research.^16,85^ They also exist in practice as interdisciplinary programmes for mixed chronic pain populations—albeit not usually incorporating manual therapy.^5,97^ Group formats and peer support are common tools in such programmes and in diabetes care,^30,63^ with some indication of benefit in DPN and neuropathic pain.^3,49,69,74,91^ Finally, qualitative research underscores the practical intervention limits created by structural barriers, patients' personal needs, and lack of peer support.^74^ To maximise population benefit, further intervention development of the NeuOst model may have to consider, first, the specific needs of patient subgroups (eg, socioeconomic, demographic, cultural, disability, and comorbidity differences); second, further adaptations could intensify some intervention components, such as the exercise programme, or add new ones, such as peer support, group sessions, or remote and digitally enabled delivery. Adaptations must also enable integration into public healthcare systems. While acknowledging limited fidelity monitoring in this trial, we have shown that osteopaths can successfully deliver the intervention. We believe this can generalise to other Allied Health Professions with graduate-level training in differential diagnosis, person-centred communication, and nonpharmacological care, assuming completion of the NeuOst training course. However, other pathway- and resource-related factors may limit the generalisability to public healthcare systems.^27^ Further intervention development may thus have to focus on scalable and low-cost intervention components and delivery models. Overall, combining physical and psychological intervention components and allowing for person-centred delivery appeared highly promising.

### 4.4. Limitations

Although our study sample included patients with considerable symptom severity, functional limitations, and comorbidity burden, we did not include people living with amputations or active ulcerations nor with severe mental illness. Attending in-person care or participating in physical therapy may not be possible for everyone experiencing such complications and comorbidities.^26,60,62,82^ The intervention was also not specifically adapted to different socioeconomic, cultural, linguistic, age, sex, or gender backgrounds, although intervention development included a diverse group of people and there was flexibility in the programme to accommodate some patient preferences and varying ability levels. Importantly, intervention development and this feasibility trial occurred outside a public healthcare system. Further stakeholder-driven work is needed here to widen participation and promote implementation of a NeuOst-like programme.

Methodologically, resource constraints led to limited diagnostic procedures and fidelity monitoring, after it emerged that more staff time would be needed for recruitment and data collection than originally expected, making the budget insufficient to fully implement other planned methods. Diagnosing neuropathy using only patient report without neurological confirmation may have increased the heterogeneity of the sample. In future trials with an explanatory attitude,^42^ neurological examinations may be desirable before enrolment (see section 4.1 of the study protocol for a discussion of the trial along the pragmatic-explanatory spectrum^52^; accessible at https://osf.io/jyftb). Alternatively, referral into the study could occur after clinical diagnosis in diabetes or neurology services, thereby also embedding the NeuOst programme into care pathways. The lack of our originally planned independent approach to fidelity monitoring through video recordings increases uncertainty, but we judged that this was a manageable risk because specific provider training was provided, supervision by an expert was available, and therapist-reported delivery was high. Nonetheless, we recognise that independent fidelity monitoring would have been helpful to better understand intervention delivery and protocol compliance, helping to interpret in particular the comparison of NeuOst to the high-similarity control intervention.

## 5. Conclusion

This feasibility trial demonstrates that a complex, multimodal intervention for people with painful diabetic polyneuropathy can be delivered safely and acceptably by trained Allied Health professionals. Despite recruitment challenges and some operational complexities inherent in running a blinded, high-similarity controlled design, key feasibility parameters were met, and participants showed substantial satisfaction and engagement. Future work should refine recruitment pathways, streamline outcome measurement, enhance fidelity monitoring, and ensure that active components are clearly differentiated from control conditions. Early clinical signals compared with usual care justify progression to a larger trial, although optimisation of the intervention may be useful, including for implementation in public healthcare systems. Given the limited efficacy of available pharmacological treatments and the clear patient demand for alternative approaches, a rigorously tested, person-centred multimodal programme such as NeuOst holds meaningful potential to address an important gap in the care of people living with painful diabetic neuropathy.

## Disclosures

D.H.S. received consulting fees from Altern Health Ltd. and income from private osteopathic practice. He reports employment with Health Sciences University and Imperial College London, and/or self-employed income from Osteopathie Schule Deutschland, Haute école de santé Fribourg, and Reflex Osteo France, where he received honoraria for teaching and supervision. He serves on the executive committee of the Society for Back Pain Research, the Scientific Committee for the 2026 International Association for the Study of Pain (IASP) World Congress, as an editor at BMC Medical Research Methodology and as a TSC/DEMC member on the AIDE-BC trial. He also received research funding from The Osteopathic Foundation, the Alan and Sheila Diamond Charitable Trust, the UK National Institute for Health and Care Research (NIHR305077), the Chelsea and Westminster Hospital Joint Research Council, and personal support through prizes or conference travel awards from SBPR, IASP, BritSpine, EFIC, SOPATE, the German Pain Research Association, and the German Diabetes Association. He received personal honoraria for research from the Analgesic, Anesthetic, and Addiction Clinical Trial Translations, Innovations, Opportunities, and Networks (ACTTION). He stands to receive personal honoraria from any future delivery of the NeuOst training course or related continuing professional development courses. S.S. is supported by research grants from Actegy Ltd (Bracknell, United Kingdom). A.B.S. receives honoraria for postgraduate teaching in the field of conservative management of neuropathic pain and consulting fees from MoreGoodDays. P.B. reports employment with Health Sciences University as a Senior Lecturer, previous salary funding as an Academic Portfolio Lead for the University of Greenwich, and he serves as the Chair of Trustees for the National Council for Osteopathic Research (NCOR) charity. He is a member of the Board of Trustees of the BCNO Group and a patient-carer representative on the committee of HeadStart, a community initiative for people living with head and neck cancer. He has received research funding from The Osteopathic Foundation, NIHR RfPB, and Medway Teaching and Learning Committee. J.D.R. received financial support from The Osteopathic Foundation. He reports employment with the National Council for Osteopathic Research and the Health Sciences University and involvement with Osteopathy Europe, where he received funding grants, nonfinancial support, and travel reimbursements. He also had nonfinancial support and travel reimbursements from the University of Technology Sydney's Faculty of Health—Australian Research Centre in Complementary and Integrative Medicine. In addition, he reports speaking and lecture fees from Kookie Learning and Metropolia University of Applied Sciences, consulting fees from the School of Health Sciences Fribourg, and employment in private osteopathic practice. D.W.E. is a co-investigator on a current research grant from the UK National Institute for Health Research. He has accepted honoraria as a visiting lecturer, for examining theses, and for an editorial role for an Elsevier journal. He receives fees for the delivery of healthcare as an osteopath. M.C.E. is funded by UKRI/Versus Arthritis, through the Advanced Pain Discovery Platform (APDP), specifically the PAINSTORM collaboration. H.K. received salary funding as an NIHR Clinical Lecturer and has received research project funding from the Academy of Medical Sciences and the British Pain Society, unrelated to the current work. N.K.E. is supported by a UKRI Natural Environmental Research Council grant (NE/Y503241/1) and an NIHR Development Award grant (NIHR506325). N.K.E. was previously employed by University College of Osteopathy and Health Sciences University as a lecturer and unit leader in Research and Enquiry (2023–2024). W.S. was previously funded through the National Institute for Health and Care Research (NIHR), Biomedical Research Centre at the South London and Maudsley NHS Foundation Trust and King's College London. The views expressed are those of the authors and not necessarily those of the NHS, the NIHR, or the Department of Health and Social Care. Whitney Scott has received funding from the Medical Research Council and Versus Arthritis Advanced Pain Discovery Platform (MR/W002388/1), unrelated to the current work. E.P. declares no interests. S.T. declares having received salary funding as a research assistant related to this project. J.V. received research funding from Viatris, paid to his institution, and consultancy fees from Grünenthal, Merz Therapeutics, and AstraZeneca, unrelated to this work. J.D.W. is a member of the German Pain Society (Deutsche Schmerzgesellschaft e.V.) and the German Society of Anaesthesiology and Intensive Care Medicine (DGAI). He received travel funding from the BIH DCSP Mobility Funds, the BIH Biomedical Innovation Academy, the Stiftung Charité, and the German Pain Society (Deutsche Schmerzgesellschaft e.V). He declares no conflicts of interest related to the topic of this study. J.W. is a participant in the BIH Charité Digital Clinician Scientist Program funded by the DFG, the Charité—Universitätsmedizin Berlin, and the Berlin Institute of Health at Charité (BIH). E.W. is funded by the Oxford and Thames Valley NIHR Applied Research Collaboration (ARC). E.P.Z., in the last 3 years, received financial support from Grüenenthal for research activities and advisory and lecture fees from Grüenenthal, MSD/Merck, and Medtronic. In addition, she receives scientific support from the German Research Foundation (DFG), the Federal Ministry of Education and Research (BMBF), the Federal Joint Committee (G-BA), and the Innovative Medicines Initiative 2 Joint Undertaking under grant agreement No 777500. This Joint Undertaking receives support from the European Union's Horizon 2020 research and innovation program and EFPIA. All money went/goes to the institutions E.P.Z. is working for. E.P.Z. is president-elect of the European Pain Federation (EFIC), past council member of the International Association for the Study of Pain (IASP), executive board member of the German Pain Society, executive board member of the Acute Pain SIG of the IASP, member of the research committee of the European Society of Anaesthesiology and Intensive Care (ESAIC), and member of the ESRA-prospect group (https://esraeurope.org/pain-management/). E.P.Z. is Deputy Editor-in-Chief for the European Journal of Anaesthesiology and the European Journal of Anaesthesiology and Intensive Care, and section editor of the European Journal of Pain. E.P.Z. regards all the above as unrelated to the current work. A.S.C.R. declares the following interests occurring in the last 36 months: Academic employee of Imperial College London. Salary funding from Chelsea and Westminster Hospital NHS Foundation Trust; Officer (President) and South Asia liaison—International Association for the Study of Pain. This includes travel, accommodation, subsistence, and hospitality from IASP and third parties; consultancy and advisory board work for Imperial College Consultants—in the last 36 months, this has included remunerated work for AstraZeneca, Pharmnovo, Vertanical, and Combigene. Additional agreements in force with SIMR Bio and Zyneyro; Patents: Rice A.S.C., Vandevoorde S., and Lambert D.M. Methods using N-(2-propenyl)hexadecanamide and related amides to relieve pain. WO 2005/079771, Okuse K. et al. Methods of treating pain by inhibition of VGF activity EP13702262.0/WO2013 110945; Member Joint Committee on Vaccine and Immunisation—varicella subcommittee until 2024; Analgesic Clinical Trial Translation: Innovations, Opportunities, and Networks (ACTTION) steering committee member until 2024; Non-Freezing Cold Injury Independent Senior Advisory Committee (NISAC): Member; Medicines and Healthcare Products Regulatory Agency (MHRA), Commission on Human Medicines—Neurology, Pain and Psychiatry Expert Advisory Group until 2024; Grants and studentships active in last 36 months—UKRI (Medical Research Council and BBSRC), Versus Arthritis, Alan and Sheila Diamond Trust, Royal British Legion, Ministry of Defence, Dr Jennie Gwynn Bequests, Royal Society of Medicine, Chelsea and Westminster Charity, and British Journal of Anaesthesia; Personal lecture and reviewer honoraria (those not donated to IASP or other charities) in last 36 months: Hospital for Special Surgery/Cornell University, 57th Congress of Japanese Society of Pain Clinicians and United Arab Emirates University. S.V. has received income from private osteopathic practice and the delivery of continuing professional development for physical therapists. He reports employment with Health Sciences University. He served on the executive committee of the Society for Back Pain Research and is currently president and Chair of trustees of the Society. He is a member and trustee of the United Kingdom Spine Societies Board and the Editor-in-Chief of the International Journal of Osteopathic Medicine. He has received research funding from the General Osteopathic Council, Versus Arthritis, The Osteopathic Foundation, Institute of Osteopathy, National Council for Osteopathic Research, Burdett Trust, European Spine Society, NIHR, and BacKCAre ESRC. He stands to receive additional employed payment from future delivery of the NeuOst training course as a continuing professional development course. The authors have no other relevant affiliations or financial involvement with any organisation or entity with a financial interest in or financial conflict with the subject matter or materials discussed in the manuscript apart from those disclosed.

## Supplementary Material

## Supplemental digital content

Supplemental digital content associated with this article can be found online at https://links.lww.com/PR9/A444.
