# Supplementary material for: A multimodal manual therapy, exercise, and psychological intervention for painful diabetic neuropathy: the NeuOst feasibility randomised controlled trial
Source: Pain Rep. 2026 Sep 16;11(5):e1498. doi: 10.1097/PR9.0000000000001498 (PMC13585324; doi:10.1097/PR9.0000000000001498)
Supplement: Supplementary file 1 [file painreports-11-e1498-s001.pdf]

Supplemental Digital Content

For:

Hohenschurz-Schmidt, D., Scott, W., Schmid, A.B., Cro, S., Bright, P., Draper-Rodi, J., Evans, D.W., Evans, M.C., Kemp, H., Koch Esteves, N., Pigott, E., Smith, S., Stynes, S., Tan, S., Vollert, J., Wandrey, J.D., Williamson, E., Pogatzki-Zahn, E.M., Rice, A.S.C. and Vogel, S., 2026. *A multimodal intervention of manual therapy, exercise, and psychological management for painful diabetic neuropathy: Results of the NeuOst feasibility randomised controlled trial*.

Content:

Part 1: Additional feasibility and clinical data page 2

Part 2: Completed reporting checklists page 14

Part 3: NeuOst intervention description and manual page 36

Part 4: Control intervention description and manual page 98

Part 1: Additional Feasibility and Clinical Data

NeuOst
A NEW APPROACH FOR PEOPLE WITH PAINFUL DIABETIC NEUROPATHY

Authors and collaborators

David Hohenschurz-Schmidt* and the NeuOst working group

*** Chief investigator:**
Imperial College London, London, UK

This document includes additional data or figures for the following measures.

Feasibility:

- Data completeness
- Qualitative participant feedback
- Fidelity of NeuOst intervention delivery
- Blinding: Reasons for allocation guesses

Clinical outcomes:

- Pain, neuropathy, quality of life, and disability measures
- Participant Global Impression of Change
- Healthcare utilisation prior to baseline
- Exercise and physical activity

Data completeness

Table S1: Instances of unsuccessful data collection. Presented as the number and percentage of incomplete data collection assessments per study arm and timepoint.

**Timepoint**	**NeuOst**<br><br>(n = 12)	**Control intervention**<br><br>(n = 12)	**Usual Care**<br><br>(n = 12)	**Total**<br><br>(n =36)
Immediate	1 (8%)	1 (8%)	2 (17%)	4 (11%)
Week 8	2 (17%)	1 (8%)	2 (17%)	5 (14%)
Week 16	2 (17%)	1 (8%)	1 (8%)	4 (11%)
All follow-ups	5 (14%)	3 (8%)	5 (14%)	**13 (12%)**

Qualitative participant feedback

Based on brief verbal feedback collected via open-ended questions at the final follow-up, participants across all groups found the trial acceptable and appreciated the personal contact and flexibility of appointments, although several challenges were noted. Many participants reported difficulty understanding or completing the outcome questionnaires, describing them as repetitive, subjective, or symptoms hard to quantify, highlighting a need for clearer and more user-friendly assessments. Some expressed uncertainties about trial procedures, payments, or group allocation, particularly in the Usual Care group, where a few participants felt frustrated at not receiving care as part of the study (likely also reflected in the low satisfaction rating for clinical care in this group, SDC Table S2). Practical barriers were also noted, such as transport, balancing study demands with health or caring responsibilities, as were occasional miscommunication about appointments or clinic logistics.

Intervention participants valued the clinical input and hands-on care but suggested longer sessions, more guidance on exercises and equipment, and greater emphasis on manual therapy elements. Across groups, participants highlighted the importance of continuity with the same provider and accessible written or digital exercise instructions. While many described the trial as beneficial - some even as “life-changing” - these repeated points indicate that refinements to communication, questionnaire design or selection, session delivery, and trial logistics could further improve both participant experience and clinical care. Detailed text comments were as follows:

Table S2: Individual responses to open-ended feedback questions.

**Question**	**Group**	**Comments** (Many participants made no comments. These are not listed below)
Challenging or confusing trial aspects	Control	- Difficult to isolate the symptoms for answering outcome questionnaires; Very useful talking to provider and very pleased with 1:1 contact; Pleased with flexibility of the clinical appointments<br>- Comments that she tried to keep an open mind about the study and group allocation; Appreciated the flexibility of appt booking<br>- doubted that she could find improvement, was very new intervention and was not clear about what was offered.<br>- has a wife who is unwell, so have to adjust timetable; transport wasn't too bad;<br>- trial itself - poorly "published"; if didn't stumble with the leaflet, had to self-refer herself; leaflet felt very small - should be larger as it contributes to health; the clinic themselves should populate it to their patients; maybe tailor it to the people with long term illnesses; collaborate more with the GPs organisation maybe need to do more for the people the clarity of the payments, as feels wasn't sure what was going to be paid or not being paid, such as though clinical visits were being paid or not for that visit. - Feels need to be more encouraged, as it takes a lot of time and effort for the participant;<br>- Had to have some aspects of the study explained more than once; e.g. moving in the wrong direction with an exercise, but did get it in the end. e.g.2 which position to start with the exercise
	NeuOst	- Travelling can be difficult at times; had to get taxi at one point was afraid to be late.<br>- one time mistake about the timings of the appointment; every other appointment was okay.<br>- None at all - felt it was straightforward<br>- Was difficult getting cover for work;<br>- telephone calls are challenging; exercises been given to respond one way or another; unable to do the exercises completely; find it difficult telephone, and prefers face-to-face.
	UC	- Did not understand why and how she was allocated to the UC group (why I wasn't able to attend your clinic). Felt reassured and good about that the information we collected from the phone assessments was of some use; but also feels that still nothing has changed in her care and is frustrated about that. Also appreciative of being able to speak to researcher for prolonged time and being asked questions about symptoms ("i'm glad that you ask me those question b/c I dont think about it until you ask me about it and then I can address it to you"; "no-one else would ask me those questions")<br>- ambiguity with the some of questions, something participant noticed. Feels difficult to quantify subjective questions.<br>- Finding some of the assessment questions repeating and confusing; and it's a lot of questions not all of which feel useful

Suggestions for improvement	Control	- Four weeks interval of assessment was good. Concerned that he might have been a bit lost of in UC group; but was pleased to be "involved and part of it"<br>- For some person in person assessments may be better<br>- would like follow-up - any way to continue; felt the treatment very helpful, especially the communication; would like more information how to return/come back<br>- Some of these questions can be difficult, and can lose concentration during the questionnaire;
	NeuOst	- Would like more hands-on in the delivery of the treatment.<br>- "felt the trial was very beneficial and life-changing." more communication with what the participant need to do; e.g. at the reception people were not aware, like which papers to sign at the clinic; didn't know whether she can take the exercise list - receptionist had to go back to clinician to get the answers, and not sure what to wear for the sessions - 1st time exercises-> wore jeans at the time which didn't help.<br>- Felt it was well organised - phoned up for the appointments; only problem - was the payment issue: reception area -> asked about the payment details, and was told quite firmly by a male receptionist that there were no payments being made for this. Said it was on the paper, and was told this was incorrect. Raised this point again, but was told that there were no payments regarding this. "payment for the sessions" -> confusing.<br>- Questionnaires – may prefer video/ticking boxes may be preferable feels detached from the study over the 4 months; hard to stay attached; at times felt rather unusual/angry having to do this even with chronic pain; feels frustrated that not really not a fix.
	UC	- difficult to quantify the subjective questioning – possible improvement in those areas – how the questionnaires are designed?
Suggestions for improving clinical care received	Control	- would be great to have longer treatment period / more treatments; Intervals and session duration good and perceived as enough; Clinical care interesting and good to learn eg about the feet that pt didn't know.<br>- Weekly intervals were good and liked having same practitioner every time. Felt a relationship building and was pleased with that (eg not having to re-explain things). Has struggled to keep up exx due to external circumstances (health complications) but will stick with NeuOst exx even if new ones are recommended Very useful talking to provider and very pleased with 1:1 contact; Pleased with flexibility of the clinical appointments<br>- Was unsure how much the wobble board should be inflated and had to take it back to clinician to check; Suggests that it should be inflated with the practitioner<br>- Would like to have the osteopath spend more time on the exercises<br>- sometimes feels lazy with the exercises;<br>- should've been told that the treatment will involve getting undressed - to clarify that in the morning;<br>- Found the different therapists very helpful/good; exercises: helpful to have a sheet/printed sheet to remind of what to do; a lot of times, therapists drew pictures but prefers a printout
	NeuOst	- Positive about the feeling after having had the "massage", and felt walking on uneven ground was less bad. Suggestion: massage could be more of it (more in depth / stronger massage of the legs at times)<br>- Would like more hands on treatment. Found the exercise using the wobble board hard to do.<br>- Having some email correspondence about what happened during the sessions and providing handouts digitally (as patient misplaced them at some point)<br>- Longer treatment sessions would have been appreciated; and to come to the clinic more; likes the "massage" element. Mentions that legs felt "so OK" and "like I was flying" for at least half an hour<br>- should be twice as long with the osteopath(!)<br>- Seems to be structured well. Found the psychological part wasn't too relevant/applicable for the patient. Feels the "distraction" part of the therapy hard/difficult to implement.<br>- felt very short;
	UC	N/A<br>- Routine foot care is not in depth and they don't really pick up on people like me and my foot problems. It would be nice to know about other care options. I don't really have any help with my feet for the neuropathy.

Fidelity of NeuOst intervention delivery

Table S3: Detailed fidelity data of NeuOst intervention delivery, presented as proportion of delivered obligatory and optional protocol items per treatment session. Based on provider self-report.

Session	% of obligatory protocol items delivered (Mean %, SD)	Proportion of high-fidelity sessions § (n, %)	% of optional protocol items delivered (Mean %, SD)	N of participants attending the session
1	84.1 (17.2)	9 (75%)	9.4 (6.3)	12
**1***	**90.9 (5.3)**	**9 (90%)**	**9.4 (5.3)**	**10**
2	89.7 (22.7)	10 (83%)	4.7 (3.3)	12
**2***	**99.4 (1.9)**	**10 (100%)**	**4.3 (2.9)**	**10**
3	86.7 (20.4)	8 (80%)	5.5 (9.3)	10
**3***	**91.9 (12.8)**	**8 (89%)**	**2.8 (3.6)**	**9**
4	89.7 (20.9)	8 (89%)	8.9 (9.3)	9
**4***	**96.4 (5.4)**	**8 (100%)**	**6.9 (7.5)**	**8**
5	87.4 (23.6)	6 (86%)	7.5 (8.5)	7
**5***	**96.1 (6.1)**	**6 (100%)**	**5.3 (6.7)**	**6**
All attended sessions	87.1%	82%		
**All attended sessions***	94.5%	95.3%		

* Correctly documented population

§ >80% obligatory item delivery

Blinding: Reasons for allocation guesses

Table S4: Reasons for allocation guesses. Presented per group as treated and per guess made. Verbal responses were categorised into all applicable response categories. Multiple categories possible per patient. Number of mentions per category presented in parentheses (multiple reasons possible). N indicating number of participants in respective group guessing the allocation presented in each row.

**Guess**	**Group alloc.**	NeuOst	Control
NeuOst		(n = 6)<br>Authenticity of experience (intensity / thoroughness) (3)<br>Recognition of familiar elements (1)<br>Clinical effects / perceived as helpful (3)	(n = 6)<br>Authenticity of experience (intensity / thoroughness) (3)<br>Recognition of familiar elements (4)<br>Clinical effects / perceived as helpful (3)
Control		(n = 3)<br>Generic conversation elements about pain or positivity (2)<br>Lack of medication prescription (1)<br>Recognition of familiar elements (1)<br>Expectations fulfilled (1)	(n = 1)<br>Inauthenticity of experience (lack of intensity) (1)<br>Clinical effects / perceived as helpful (1)
Don't know		(n = 2)<br>Unawareness of criteria for intervention vs control (1)<br>Not seeing relevance of treatment ("not related to root causes") (1)	(n = 4)<br>Unawareness of criteria for intervention vs control (1)<br>Authenticity but unable to differentiate (1)<br>'Unsure' (1)<br>No recall of intervention (1)

Numerical data for clinical outcome measures

Pain, neuropathy, quality of life, and disability measures – descriptive statistics

Table S5: Descriptive statistics of clinical outcome measures. Data are presented for the entire sample at baseline (Total), and per group for each timepoint. Values are Mean and Standard Deviation, unless otherwise indicated. For each group and follow-up, the first column (Absol.) indicates absolute values for the group, while the second column (Change) indicates within-group change scores from baseline. Data are presented for all participants with available data at each timepoint. Control = High-similarity control intervention; NeuOst = NeuOst intervention; UC = Usual Care.

**Outcome measure**	**Baseline**				**Immediate**						**8 weeks**						**16 weeks**					
	Total (n = 36)	Control (n=12)	NeuOst (n=12)	UC (n=12)	Control (n=11)		NeuOst (n=11)		UC (n=10)		Control (n=11)		NeuOst (n=10)		UC (n=10)		Control (n=11)		NeuOst (n=10)		UC (n=11)	
					Absol.	Change	Absol.	Change	Absol.	Change	Absol.	Change	Absol.	Change	Absol.	Change	Absol.	Change	Absol.	Change	Absol.	Change
**Brief Pain Inventory**																						
Worst pain	6.9 (1.7)	5.8 (1.9)	7.3 (1.4)	7.5 (1.2)	5.3 (2.6)	-0.6 (1.9)	5.1 (2.9)	-2.1 (3.3)	7.1 (2.8)	-0.3 (2.3)	5.2 (3.5)	-0.7 (3.4)	5.3 (2.6)	-1.9 (2.8)	7.9 (1.4)	0.1 (1.5)	4.6 (2.7)	-1.3 (2.4)	5.9 (2.7)	-1.3 (3.0)	5.7 (3.4)	-1.8 (2.5)
Average pain	5.0 (1.9)	4.3 (1.8)	5.5 (1.7)	5.3 (1.9)	4.1 (2.9)	-0.4 (2.2)	3.7 (2.4)	-1.6 (2.8)	4.7 (2.4)	-0.1 (2.0)	3.5 (2.8)	-1.0 (2.2)	3.8 (1.8)	-1.4 (2.3)	5.0 (1.8)	-0.3 (2.3)	3.3 (2.6)	-1.2 (2.3)	3.5 (2.5)	-2.1 (2.3)	4.5 (2.8)	-0.7 (1.9)
Pain severity	4.51 (1.70)	3.7 (1.8)	5.1 (1.6)	4.8 (1.5)	3.8 (2.9)	0.0 (1.8)	3.9 (2.5)	-1.1 (3.1)	4.4 (2.0)	-0.1 (1.3)	3.6 (2.8)	-0.3 (2.1)	3.4 (1.7)	-1.4 (2.0)	4.9 (1.6)	0.0 (1.5)	2.8 (1.9)	-1.1 (1.1)	4.0 (2.5)	-1.1 (2.8)	3.7 (2.3)	-0.9 (1.0)
Pain interference	4.8 (2.5)	4.5 (2.7)	5.1 (2.1)	4.8 (2.8)	2.5 (2.8)	-2.2 (2.8)	3.4 (2.9)	-1.6 (3.7)	4.4 (2.9)	0.2 (2.2)	3.1 (3.0)	-1.7 (2.8)	3.5 (3.1)	-1.4 (3.3)	5.5 (2.7)	0.6 (1.7)	2.5 (2.6)	-2.3 (2.9)	3.0 (2.7)	-1.8 (3.3)	3.5 (2.9)	-1.0 (2.0)
**Symptom bothers. and tolerability**																						
**Days bothersome DPN symptoms in past week**	5.9 (1.8)	5.9 (2.0)	6.2 (1.4)	5.7 (2.0)	4.4 (3.1)	- 1.5 (3.2)	4 (2.8)	- 2.4 (2.3)	5.4 (2.4)	-0.2 (3.0)	4.3 (3.2)	-1.6 (2.6)	4.6 (2.4)	-1.7 (1.6)	6.1 (2.2)	0.7 (1.4)	4.3 (2.9)	-1.6 (2.7)	4.6 (2.8)	-1.7 (-1.0	4.9 (2.4)	-0.6 (1.6)
**Days intolerable in past week**	1.7 (2.2)	1.9 (2.8)	1.7 (1.8)	1.5 (2.0)	1.1 (2.2)	-1.0 (3.4)	0.8 (1.5)	-0.6 (2.4)	0.8 (1.4)	-0.5 (1.9)	1.3 (2.6)	-0.8 (3.3)	1.4 (2.1)	0.0 (2.8)	2.5 (2.2)	0.7 (3.0)	0.9 (1.6)	-1.2 (2.6)	1.1 (2.3)	-0.4 (2.0)	1.5 (2.2)<br>0 (0-6)	-0.2 (1.5)
**NeuroQoL*** By domain																						
Painful sensations (frequency)	2.8 (0.8)	2.8 (0.9)	2.8 (0.8)	2.8(0.9)	2.3 (0.9)	-0.5 (0.75)	2.3 (0.9)	-0.5 (0.9)	2.8 (0.7)<br>3.0 (1.7	0.2 (0.6)	2.2 (0.7)	-0.7 (0.6)	2.3 (0.7)	-0.4 (0.4)	2.8 (0.9)	-0.0 (0.8)	2.25 (0.83)	-0.62 (0.81)	2.11 (0.85)	-0.56 (0.67)	2.42 (0.64)	-0.34 (0.67)
Reduced sensation (frequency)	2.5 (1.2)	2.4 (1.2)	2.8 (1.5)	2.2 (1.0)	2.0 (1.0)	-0.1 (0.8)	2.4 (1.3)	-0.6 (0.8)	2.7 (1.0)	0.4 (0.6)	1.7 (0.7)	-0.4 (0.6)	2.0 (0.7)	-1.0 (1.0)	2.7 (1.1)	0.3 (0.9)	1.76 (0.94)	-0.39 (0.87)	2.17 (1.42)	-0.77 (1.23)	2.67 (1.14)	0.36 (0.82)
Diffuse sensorimotor symptoms (frequency)	2.6 (1.2)	2.7 (1.3)	2.5 (1.2)	2.4 (1.2)	2.3 (1.0)	-0.6 (0.9)	2.2 (1.2)	-0.1 (0.8)	2.3 (0.9)	0.1 (0.9)	2.2 (0.9)	-0.7 (0.8)	2.3 (1.0)	-0.2 (0.7)	2.3 (1.0)	-0.2 (1.0)	1.88 (0.69)	-1.00 (1.15)	2.37 (1.22)	0.07 (0.81)	2.21 (0.83)	-0.09 (0.82)
Daily activity impact of foot symptoms	2.9 (1.3)	3.0 (1.3)	2.7 (1.3)	3.1 (1.4)	2.3 (1.4)	-0.9 (1.1)	2.0 (1.0)	-0.7 (1.2)	2.8 (1.3)	0.1 (1.3)	2.1 (1.1)	-1.1 (0.9)	2.3 (1.1)	-0.4 (1.2)	2.6 (1.2)	-0.5 (1.2)	2.24 (1.29)	-0.94 (1.04)	1.90 (1.08)	-0.63 (1.08)	2.24 (1.01)	-0.67 (1.09)
Social impact	2.3 (1.2)	2.1 (1.1)	2.6 (1.1)	2.3 (1.4)	1.9 (1.1)	-0.3 (0.9)	1.8 (1.0)	-0.8 (0.9)	2.3 (1.1)	0.4 (0.9)	1.93 (1.25)	-0.20 (0.63)	1.90 (1.32)	-0.55 (0.99)	2.33 (1.12)	0.00 (1.18)	1.68 (1.19)	-0.45 (0.78)	1.48 (0.92)	-0.85 (1.01)	1.86 (1.06)	-0.34 (1.04)
Emotional distress	3.3 (1.1)	3.2 (1.0)	3.6 (0.6)	3.0 (1.4)	3.0 (1.1)	-0.2 (1.1)	2.7 (1.4)	-1.0 (1.1)	2.7 (1.2)	0.0 (0.8)	2.87 (1.36)	-0.27 (1.10)	2.71 (1.40)	-0.89 (0.95)	2.89 (1.31)	-0.17 (1.21)	2.75 (1.36)	-0.39 (1.08)	2.51 (1.11)	-1.06 (0.86)	2.68 (1.21)	-0.19 (0.96)
Average NeuroQoL score across all domain items	2.8 (0.8)	2.8 (0.7)	2.9 (0.7)	2.7 (1.0)	2.4 (0.9)	-0.4 (0.6)	2.3 (0.9)	-0.6 (0.6)	2.6 (0.7)	0.2 (0.6)	2.3 (0.9)	-0.5 (0.5)	2.3 (0.9)	-0.6 (0.5)	2.7 (0.8)	-0.1 (0.8)	2.2 (0.9)	-0.6 (0.7)	2.1 (0.9)	-0.7 (0.7)	2.4 (0.8)	-0.2 (0.7)
Impact of foot symptoms on QoL	2.9 (1.3)	2.6 (1.3)	3.1 (1.1)	3.0 (1.6)	2.64 (1.29)	-0.82 (1.08)	3.09 (1.14)	-0.82 (1.18)	3.0 (1.56)	0.3 (0.95)	2.55 (1.04)	-0.91 (1.14)	3.1 (1.37)	-0.9 (1.45)	3.0 (1.41)	-0.1 (0.99)	2.45 (1.23)	-1.0 (1.34)	2.5 (1.27)	-1.3 (1.25)	2.73 (1.42)	-0.18 (0.6)
Overall QoL rating	3.0 (0.8)	3.1 (0.9)	3.3 (0.6)	2.7 (0.8)	3.09 (0.94)	0.34 (1.2)	3.27 (0.65)	0.73 (0.9)	2.7 (0.82)	0.2 (0.79)	3.27 (1.0)	0.55 (1.13)	2.8 (0.63)	0.2 (0.79)	2.5 (1.08)	0.3 (0.67)	3.0 (1.1)	0.27 (1.42)	2.8 (1.03)	0.2 (0.63)	2.64 (1.2)	0.27 (0.65)

**DPNPI ****																						
Impact on physical function	56.77 (27.97)	60.9 (25.9)	56.0 (25.8)	53.4 (33.5)	40.63 (24.49)	-25.85 (18.86)	42.61 (26.12)	-10.8 (17.31)	52.5 (25.12)	7.81 (26.97)	40.34 (22.25)	-26.14 (17.36)	47.19 (24.98)	-7.5 (24.88)	51.56 (29.95)	-3.13 (19.82)	41.76 (26.45)	-24.72 (18.72)	41.25 (29.38)	-9.06 (30.35)	46.02 (30.07)	-3.69 (18.53)
Impact on sleep	56.11 (33.83)	57.5 (27.7)	55.8 (38.9)	55 (36.9)	47.73 (27.24)	-10.0 (19.1)	39.46 (35.46)	-12.73 (25.92)	62.5 (28.7)	12.0 (32.68)	47.73 (28.67)	-10.0 (20.49)	36.5 (36.75)	-13.5 (22.86)	54.5 (29.86)	-5.5 (26.4)	43.18 (33.26)	-14.55 (26.59)	26.0 (30.89)	-22.5 (31.91)	46.81 (32.88)	-7.73 (30.61)
Impact on daily function	44.72 (24.32)	47.9 (19.7)	45.0 (24.8)	41.3 (29.2)	33.18 (29.86)	-15.01 (16.01)	32.27 (31.01)	-10.45 (23.29)	38.5 (22.12)	3.5 (31.98)	27.27 (26.49)	-21.82 (17.22)	35.0 (25.93)	-7.0 (19.03)	38.0 (26.48)	-4.0 (19.69)	34.55 (32.9)	-14.55 (20.43)	29.0 (31.52)	-10.5 (26.4)	37.73 (27.96)	-0.45 (22.96)
**Total DPNPI score**	**53.24 (24.61)**	**56.4 (20.3)**	**52.9 (24.2)**	**50.5 (30.1)**	**40.53 (24.31)**	**-18.69 (15.35)**	**38.89 (27.74)**	**-11.24 (17.29)**	**51.39 (18.04)**	**7.78 (28.71)**	**38.76 (23.36)**	**-20.45 (14.88)**	**40.83 (24.94)**	**-9.03 (19.44)**	**48.61 (25.03)**	**-4.03 (19.53)**	**40.15 (29.22)**	**-19.07 (18.41)**	**33.61 (25.6)**	**-13.19 (23.56)**	**43.94 (28.12)**	**-3.91 (21.49)**
Impact on social function ***	36.7 (28.5)	38.3 (31.3)	35.0 (27.1)	36.7 (29.3)	27.3 (32.6)	-14.5 (27.0)	18.2 (27.5)	-14.5 (23.8)	26.0 (26.7)	-4.0 (24.6)	25.5 (31.1)	-16.4 (28.0)	30.0 (28.7)	-2.0 (27.4)	34.3 (28.4)	-4.0 (22.7)	25.5 (31.1)	-16.4 (26.6)	14.0 (25.0)	-14.0 (21.2)	27.3 (25.7)	-7.3 (20.5)
**FES-I** Falls Efficacy scale																						
**Total score**	**16.7 (6.6)**	**17.6 (6.1)**	**16.2 (7.3)**	**16.3 (6.8)**	**16.4 (5.3)**	**-2.2 (4.2)**	**13.9 (6.3)**	**-1.7 (5.8)**	**14.6 (5.1) 15 (7-22)**	**-0.3 (6.2)**	**15.8 (6.6)**	**-2.7 (6.0)**	**13.8 (6.2)**	**-2.3 (5.2)**	**14.5 (5.3)**	**-2.1 (5.0)**	**17 (6.5)**	**-1.6 (3.2)**	**13.1 (6.9)**	**-1.9 (6.1)**	**14.7 (6.5)**	**-1.0 (6.0)**
Low	5 (14%)	1 (8%)	2 (17%)	2 (17%)	1 (9%)	+1%	3 (27%)	+10%	1 (10%)	-7%	1 (9%)	-1%	2 (20%)	+3%	1 (10%)	-7%	1 (9%)	+1%	3 (30%)	+13%	2 (18%)	+1%
Moderate	9 (25%)	2 (17%)	4 (33%)	3 (25%)	1 (9%)	-8%	3 (27%)	-6%	3 (30%)	+5%	3 (27%)	+10%	3 (30%)	-3%	3 (30%)	+5%	2 (18%)	-1%	4 (40%)	+7%	4 (36%)	+11%
High concern	22 (61%)	9 (75%)	6 (50%)	7 (58%)	9 (82%)	+7%	5 (45%)	-13%	6 (60%)	+2%	7 (64%)	-11%	5 (50%)	+/-0%	6 (60%)	+2%	8 (73%)	+2%	3 (30%)	-20%	5 (45%)	-13%

* Note that the Neuro-QoL was assessed in a non-weighted manner. This means that the 27 symptom- or domain-specific individual items were not additionally rated for their importance or bothersomeness to the patient, as can be done to "provide a more detailed picture of the degree to which the specific experience impacts satisfaction or quality of life" (as per NeuroQoL scoring manual). The summary score is thus simply additive, with a higher score indicating lower QoL related to neuropathy. Weighted rating was omitted due to the length of the questionnaire and burden to participants.

** DPNPI (Diabetic Peripheral Neuropathy Pain Impact measure) domain and total scores are converted into a 0-100 scale, with higher scores indicating worse health.

*** Social function is a single DPNPI question, not part of the total DPNPI score. For illustration purposes, the question has been converted to the same 0-100 scale as the remaining, combined DPNPI domains.

Participant Global Impression of Change, descriptive statistics

Table S6: Participant Global Impression of Change. Descriptive statistics of individuals rating the change in their overall health according to each PGIC domain and per group. Data presented for immediate follow-up, i.e., at the end of the treatment period or 5-weeks after randomisation; at follow-up 1 (8 weeks after randomisation); and at follow-up 2 (16 weeks after randomisation).

**Immediate**	**Control intervention**		**NeuOst**		**Usual Care**	
n	11		11		10	
Very much improved	2	18.2%	2	18.2%	0	0%
Much improved	3	27.3%	5	45.5%	1	10%
Minimally improved	3	27.3%	2	18.2%	1	10%
No Change	3	27.3%	2	18.2%	7	70%
Minimally Worse	0	0%	0	0%	1	10%
Much Worse	0	0%	0	0%	0	0%
Very much worse	0	0%	0	0%	0	0%
**Follow-up 1**	**Control intervention**		**NeuOst**		**Usual Care**	
n	11		10		10	
Very much improved	2	18.2%	0	0%	0	0%
Much improved	6	54.6%	4	40%	0	0%
Minimally improved	2	18.2%	2	20%	2	20%
No Change	1	9.1%	4	40%	4	40%
Minimally Worse	0	0%	0	0%	2	20%
Much Worse	0	0%	0	0%	2	20%
Very much worse	0	0%	0	0%	0	0%
**Follow-up 2**	**Control**		**NeuOst**		**Usual Care**	
n	11		10		11	
Very much improved	4	36.4%	1	10%	1	9.1%
Much improved	4	36.4%	2	20%	1	9.1%
Minimally improved	1	9.1%	4	40%	2	18.2%
No Change	2	18.2%	0	0%	4	36.4%
Minimally Worse	0	0%	3	30%	1	9.1%
Much Worse	0	0%	0	0%	2	18.2%
Very much worse	0	0%	0	0%	0	0%

Healthcare utilisation, descriptive statistics

Table S7: Healthcare utilisation in the preceding four weeks. Collected at baseline.

**Healthcare service used in past 4 weeks**	**Total** (n = 36)	**NeuOst** (n = 12)	**Control intervention** (n = 12)	**Usual Care** (n = 12)
None	5 (14% of patients)	3 (25% of patients)	1 (8% of patients)	1 (8% of patients)
Endocrinologist / Diabetologist	5	2	2	1
GP	12	1	6	5
Diabetes nurse	14	4	3	7
Podiatrist / Chiropodist	3		1	2
Other specialist doctor	13	3, of which:<br><br>2 (cardiologist)<br>1 (pain specialist)	5, of which:<br><br>1 (gastro-enterologist)<br>3 (ophthalm.)<br>1 (surgeon)	5, of which:<br><br>1 (nephrologist)<br>2 (ophthalm.)<br>1 (pain specialist)<br>1 (surgeon)
Physiotherapist	2	1	0	1
Fitness coach	1	1	0	0
Dietician	2	1	0	1
Manual Therapist	1	0	0	1
Alternative Therapy Provider	1	0	0	1
Dentist	1	0	1	0
Emergency or Urgent Care	1	1	1	0
Online or print materials	7	1	2	4
**Average number of different services used per patient ***	1.6 (1.3)<br>Median, range: 1.0 (0 – 6)	1.2 (0.9)<br>Median, range: 1.0 (0 – 3)	1.9 (1.0)<br>Median, range: 2.0 (1 – 4)	1.8 (1.6)<br>Median, range: 1.5 (0 – 6)
**Total** (individual services consulted)	**63**	**12**	**16**	**23**

* Not counting the use of online or print materials about healthcare-related topics

Exercise and physical activity, descriptive statistics

Figure S1: Spaghetti plots of self-reported stretch, strength and stability exercise levels in the past week across timepoints.

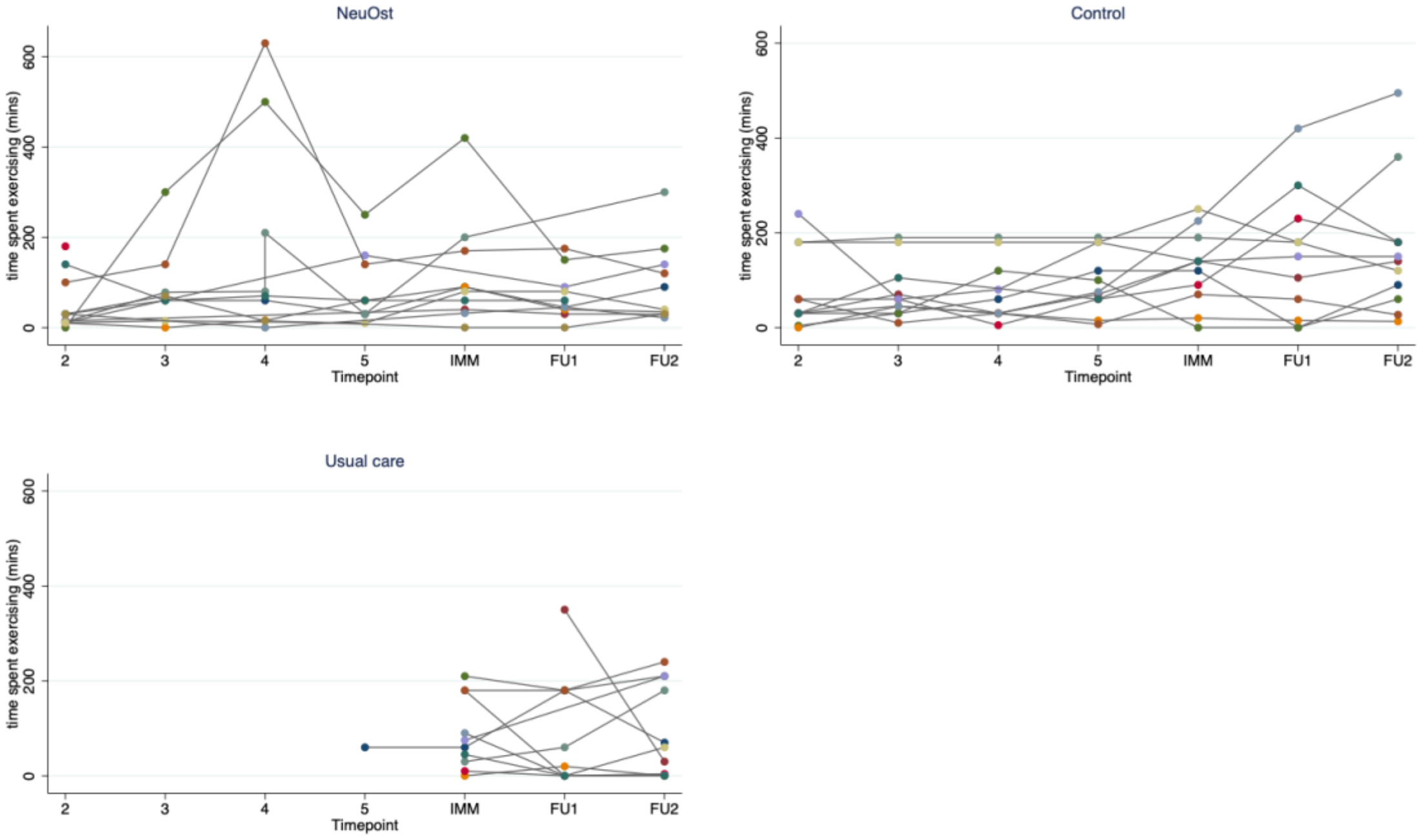

Figure S2: Spaghetti plots of self-reported cardiovascular activity levels in the past week across timepoints:

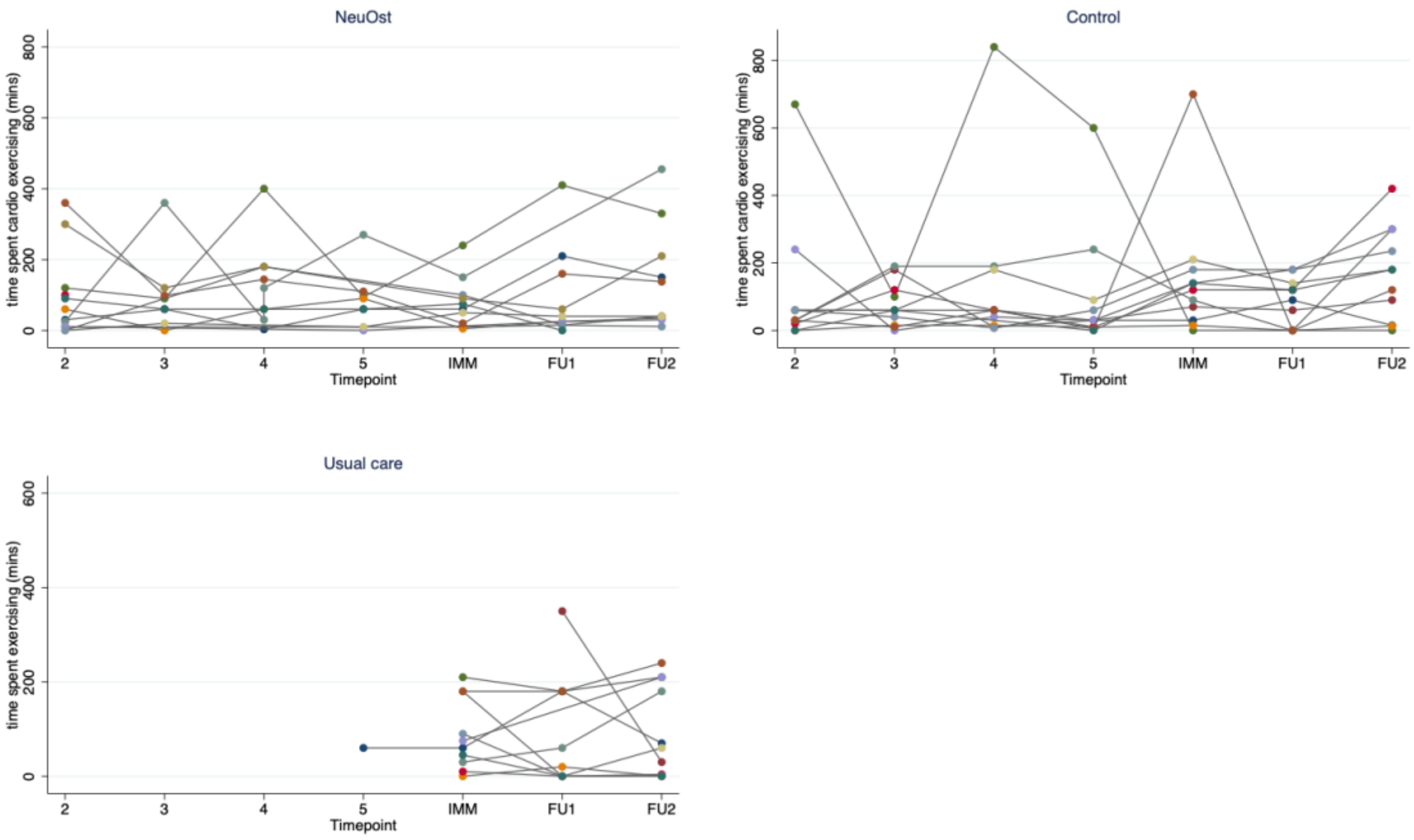

Table S8: Time spent doing strength, stretch or stability exercises in past week (in minutes).

Timepoint	Control	NeuOst	UC
Visit 2	76.7 (82.7)<br>n =11	47.5 (59.1)<br>n =12	
Visit 3	75.0 (59.7)<br>n =11	90.3 (94.6)<br>N = 8	
Visit 4	75.5 (66.3)<br>n =10	175.7 (231.4)<br>N = 9	
Visit 5	96.1 (64.6)<br>n =11	92.5 (83.1)<br>N = 8	
Immediate	125.9 (78.4)<br>n =11	118.2 (122.2)<br>N =10	n =10<br>124.0 (137.4)
8 weeks	149.1 (133.0)<br>n =11	71.5 (54.4)<br>N =10	109 (121.9)<br>n =10
16 weeks	165 (144.6)<br>n =11	98.2 (89.1)<br>N =10	91.4 (97.9)<br>n =11

Table S9: Time spent doing cardiovascular exercise in past week (in minutes).

Timepoint	Control	NeuOst	UC
Visit 2	109.1 (197.4)<br>n =11	92.2 (118.9)<br>n =12	
Visit 3	75.9 (65.0)<br>n =11	101.0 (111.9)<br>n = 8	
Visit 4	146.2 (252.4)<br>n =10	130.8 (119.4)<br>n = 9	
Visit 5	100.5 (179.2)<br>n =11	86.9 (83.7)<br>n = 8	
Immediate	154.1 (193.1)<br>n =11	80.0 (71.8)<br>n =10	133.5 (92.3)<br>n =10
8 weeks	80.9 (72.7)<br>n =11	96.0 (130.2)<br>n =10	136.5 (110.1)<br>n =10
16 weeks	168.5 (136.8)<br>n =11	143.9 (149.1)<br>n =10	154.9 (169.4)<br>n =11

Supplemental Digital Content, part 2: Completed reporting checklists

Completed 2025 CONSORT Checklist for

'A multimodal intervention of manual therapy, exercise, and psychological management for painful diabetic neuropathy: Results of the NeuOst feasibility randomised controlled trial'

Section/topic	No	CONSORT 2025 checklist item description	Reported on page no.
**Title and abstract**			
Title and structured abstract	1a	Identification as a randomised trial	Title
	1b	Structured summary of the trial design, methods, results, and conclusions	Abstract
**Open science**			
Trial registration	2	Name of trial registry, identifying number (with URL) and date of registration	Methods section: Pre-registration & Abstract
Protocol and statistical analysis plan	3	Where the trial protocol and statistical analysis plan can be accessed	Methods section: Pre-registration & Open Science and data access paragraphs
Data sharing	4	Where and how the individual de-identified participant data (including data dictionary), statistical code and any other materials can be accessed	Methods section: 1st paragraph
Funding and conflicts of interest	5a	Sources of funding and other support (eg, supply of drugs), and role of funders in the design, conduct, analysis and reporting of the trial	Methods: Funders paragraph
	5b	Financial and other conflicts of interest of the manuscript authors	Declarations of interest
**Introduction**			
Background and rationale	6	Scientific background and rationale	Introduction & protocol paper (doi 10.1080/17581869.2025.2515010)
Objectives	7	Specific objectives related to benefits and harms	Introduction: final paragraph
**Methods**			
Patient and public involvement	8	Details of patient or public involvement in the design, conduct and reporting of the trial	Methods: PPI paragraph & Intervention development paper (doi 10.1080/17581869.2025.2515010)
Trial design	9	Description of trial design including type of trial (eg, parallel group, crossover), allocation ratio, and framework (eg, superiority, equivalence, non-inferiority, exploratory)	Methods: trial design paragraph
Changes to trial protocol	10	Important changes to the trial after it commenced including any outcomes or analyses that were not prespecified, with reason	Methods: paragraph on Changes to trial protocol
Trial setting	11	Settings (eg, community, hospital) and locations (eg, countries, sites) where the trial was conducted	Methods: Trial setting paragraph

Eligibility criteria	12a	Eligibility criteria for participants	Methods: Eligibility criteria paragraph
	12b	If applicable, eligibility criteria for sites and for individuals delivering the interventions (eg, surgeons, physiotherapists)	Methods: Eligibility criteria paragraph
Intervention and comparator	13	Intervention and comparator with sufficient details to allow replication. If relevant, where additional materials describing the intervention and comparator (eg, intervention manual) can be accessed	Methods: Registered protocol, prior publication & Supplemental Digital Content
Outcomes	14	Prespecified primary and secondary outcomes, including the specific measurement variable (eg, systolic blood pressure), analysis metric (eg, change from baseline, final value, time to event), method of aggregation (eg, median, proportion), and time point for each outcome	Methods: Outcomes and data collection timepoints paragraph
Harms	15	How harms were defined and assessed (eg, systematically, non-systematically)	Methods: Harms paragraph
Sample size	16a	How sample size was determined, including all assumptions supporting the sample size calculation	Methods: Sample size and power paragraph
	16b	Explanation of any interim analyses and stopping guidelines	Methods: Statistical methods and analysis paragraph
Randomisation:			
Sequence generation	17a	Who generated the random allocation sequence and the method used	Methods: Randomisation paragraph
	17b	Type of randomisation and details of any restriction (eg, stratification, blocking and block size)	Methods: Randomisation paragraph
Allocation concealment mechanism	18	Mechanism used to implement the random allocation sequence (eg, central computer/telephone; sequentially numbered, opaque, sealed containers), describing any steps to conceal the sequence until interventions were assigned	Methods: Randomisation paragraph
Implementation	19	Whether the personnel who enrolled and those who assigned participants to the interventions had access to the random allocation sequence	Methods: Randomisation paragraph
Blinding	20a	Who was blinded after assignment to interventions (eg, participants, care providers, outcome assessors, data analysts)	Methods: Blinding paragraph
	20b	If blinded, how blinding was achieved and description of the similarity of interventions	Methods: Blinding & Intervention and comparator paragraphs; Also Supplemental Digital Content and separate reporting checklist
Statistical methods	21a	Statistical methods used to compare groups for primary and secondary outcomes, including harms	Methods: Statistical methods and analysis paragraph
	21b	Definition of who is included in each analysis (eg, all randomised participants), and in which group	Methods: Statistical methods and analysis paragraph
	21c	How missing data were handled in the analysis	Methods: Statistical methods and analysis paragraph
	21d	Methods for any additional analyses (eg, subgroup and sensitivity analyses), distinguishing prespecified from post hoc	Methods: Statistical methods and analysis paragraph
**Results**			
Participant flow, including flow diagram	22a	For each group, the numbers of participants who were randomly assigned, received intended intervention, and were analysed for the primary outcome	Results: Participant flow paragraph and CONSORT diagram
	22b	For each group, losses and exclusions after randomisation, together with reasons	Results: CONSORT diagram

Recruitment	23a	Dates defining the periods of recruitment and follow-up for outcomes of benefits and harms	Methods: Recruitment and consent & Outcomes and data collection timepoints
	23b	If relevant, why the trial ended or was stopped	Results: Participant flow and CONSORT diagram paragraph
Intervention and comparator delivery	24a	Intervention and comparator as they were actually administered (eg, where appropriate, who delivered the intervention/comparator, how participants adhered, whether they were delivered as intended (fidelity))	Results: Provider characteristics & Primary analysis: Feasibility and acceptability paragraphs
	24b	Concomitant care received during the trial for each group	Results: Secondary outcomes (clinical measures) – paragraph on Healthcare utilisation and medication use; Also: Supplemental Digital Content
Baseline data	25	A table showing baseline demographic and clinical characteristics for each group	Results: Sample characteristics at baseline paragraph and table
Numbers analysed, outcomes and estimation	26	For each primary and secondary outcome, by group:<br>● the number of participants included in the analysis<br>● the number of participants with available data at the outcome time point<br>● result for each group, and the estimated effect size and its precision (such as 95% confidence interval)<br>● for binary outcomes, presentation of both absolute and relative effect size	Results: Participant flow and CONSORT diagram paragraph & Respective results sections; Also: Supplemental Digital Content for secondary and exploratory outcomes
Harms	27	All harms or unintended events in each group	Results: Primary analysis: Feasibility and acceptability paragraphs – Adverse Events section and table
Ancillary analyses	28	Any other analyses performed, including subgroup and sensitivity analyses, distinguishing pre-specified from post hoc	Results: Subgroup and sensitivity analyses paragraph
**Discussion**			
Interpretation	29	Interpretation consistent with results, balancing benefits and harms, and considering other relevant evidence	Discussion section
Limitations	30	Trial limitations, addressing sources of potential bias, imprecision, generalisability, and, if relevant, multiplicity of analyses	Discussion: Limitations section

Citation: Hopewell S, Chan AW, Collins GS, Hróbjartsson A, Moher D, Schulz KF, et al. CONSORT 2025 Statement: updated guideline for reporting randomised trials. BMJ. 2025; 388:e081123. https://dx.doi.org/10.1136/bmj-2024-081123

© 2025 Hopewell et al. This is an Open Access article distributed under the terms of the Creative Commons Attribution License (https://creativecommons.org/licenses/by/4.0/), which permits unrestricted use, distribution, and reproduction in any medium, provided the original work is properly cited.

*We strongly recommend reading this statement in conjunction with the CONSORT 2025 Explanation and Elaboration and/or the CONSORT 2025 Expanded Checklist for important clarifications on all the items. We also recommend reading relevant CONSORT extensions. See www.consort-spirit.org.

CONSORT 2010 checklist of information to include when reporting a pilot or feasibility trial* - completed for: 'A multimodal intervention of manual therapy, exercise, and psychological management for painful diabetic neuropathy: Results of the NeuOst feasibility randomised controlled trial'

Section/Topic	Item No	Checklist item	Reported on page No
**Title and abstract**			
	1a	Identification as a pilot or feasibility randomised trial in the title	Title
	1b	Structured summary of pilot trial design, methods, results, and conclusions (for specific guidance see CONSORT abstract extension for pilot trials)	Abstract
**Introduction**			
Background and objectives	2a	Scientific background and explanation of rationale for future definitive trial, and reasons for randomised pilot trial	Introduction
	2b	Specific objectives or research questions for pilot trial	Introduction
**Methods**			
Trial design	3a	Description of pilot trial design (such as parallel, factorial) including allocation ratio	Methods: Trial design paragraph
	3b	Important changes to methods after pilot trial commencement (such as eligibility criteria), with reasons	Methods: Changes to trial protocol
Participants	4a	Eligibility criteria for participants	Methods: Eligibility criteria
	4b	Settings and locations where the data were collected	Results: Trial setting
	4c	How participants were identified and consented	Methods: Recruitment and consent
Interventions	5	The interventions for each group with sufficient details to allow replication, including how and when they were actually administered	Methods: Registered protocol, prior publication & Supplemental Digital Content

Outcomes	6a	Completely defined prespecified assessments or measurements to address each pilot trial objective specified in 2b, including how and when they were assessed	Methods: Outcomes and data collection timepoints
	6b	Any changes to pilot trial assessments or measurements after the pilot trial commenced, with reasons	Methods: Changes to trial protocol
	6c	If applicable, prespecified criteria used to judge whether, or how, to proceed with future definitive trial	Methods: Statistical methods and analysis
Sample size	7a	Rationale for numbers in the pilot trial	Methods: Sample size and power
	7b	When applicable, explanation of any interim analyses and stopping guidelines	Methods: Statistical methods and analysis
Randomisation:			
Sequence generation	8a	Method used to generate the random allocation sequence	Methods: Randomisation
	8b	Type of randomisation(s); details of any restriction (such as blocking and block size)	Methods: Randomisation
Allocation concealment mechanism	9	Mechanism used to implement the random allocation sequence (such as sequentially numbered containers), describing any steps taken to conceal the sequence until interventions were assigned	Methods: Randomisation
Implementation	10	Who generated the random allocation sequence, who enrolled participants, and who assigned participants to interventions	Methods: Randomisation
Blinding	11a	If done, who was blinded after assignment to interventions (for example, participants, care providers, those assessing outcomes) and how	Methods: Blinding
	11b	If relevant, description of the similarity of interventions	Methods: Blinding & Intervention and comparator paragraphs; Also Supplemental Digital Content and separate reporting checklist

Statistical methods	12	Methods used to address each pilot trial objective whether qualitative or quantitative	Methods: Statistical methods and analysis
**Results**			
Participant flow (a diagram is strongly recommended)	13a	For each group, the numbers of participants who were approached and/or assessed for eligibility, randomly assigned, received intended treatment, and were assessed for each objective	Results: Participant flow and CONSORT diagram & Primary analysis: Feasibility and acceptability
	13b	For each group, losses and exclusions after randomisation, together with reasons	Results: Participant flow and CONSORT diagram
Recruitment	14a	Dates defining the periods of recruitment and follow-up	Results: Recruitment, eligibility, and consent rates
	14b	Why the pilot trial ended or was stopped	Results: Participant flow and CONSORT diagram paragraph
Baseline data	15	A table showing baseline demographic and clinical characteristics for each group	Results: Sample characteristics at baseline paragraph and table
Numbers analysed	16	For each objective, number of participants (denominator) included in each analysis. If relevant, these numbers<br><br>should be by randomised group	Results: Participant flow and CONSORT diagram paragraph & Respective results sections; Also: Supplemental Digital Content for secondary and exploratory outcomes
Outcomes and estimation	17	For each objective, results including expressions of uncertainty (such as 95% confidence interval) for any estimates. If relevant, these results should be by randomised group	Results: Respective results sections; Also: Supplemental Digital

			Content for secondary and exploratory outcomes
Ancillary analyses	18	Results of any other analyses performed that could be used to inform the future definitive trial	Results: Respective results sections; Also: Supplemental Digital Content for secondary and exploratory outcomes
Harms	19	All important harms or unintended effects in each group (for specific guidance see CONSORT for harms)	Results: Primary analysis: Feasibility and acceptability paragraphs – Adverse Events section and table
	19a	If relevant, other important unintended consequences	Not relevant
**Discussion**			
Limitations	20	Pilot trial limitations, addressing sources of potential bias and remaining uncertainty about feasibility	Discussion section, incl. Limitations
Generalisability	21	Generalisability (applicability) of pilot trial methods and findings to future definitive trial and other studies	Discussion section, incl. Limitations
Interpretation	22	Interpretation consistent with pilot trial objectives and findings, balancing potential benefits and harms, and considering other relevant evidence	Discussion section
	22a	Implications for progression from pilot to future definitive trial, including any proposed amendments	Discussion section
**Other information**			
Registration	23	Registration number for pilot trial and name of trial registry	Abstract & Methods: Pre-registration
Protocol	24	Where the pilot trial protocol can be accessed, if available	Methods: Pre-registration
Funding	25	Sources of funding and other support (such as supply of drugs), role of funders	Methods: Funders

	26	Ethical approval or approval by research review committee, confirmed with reference number	Methods: Sponsor and ethics

Citation: Eldridge SM, Chan CL, Campbell MJ, Bond CM, Hopewell S, Thabane L, et al. CONSORT 2010 statement: extension to randomised pilot and feasibility trials. BMJ. 2016;355.
*We strongly recommend reading this statement in conjunction with the CONSORT 2010, extension to randomised pilot and feasibility trials, Explanation and Elaboration for important clarifications on all the items. If relevant, we also recommend reading CONSORT extensions for cluster randomised trials, non-inferiority and equivalence trials, non-pharmacological treatments, herbal interventions, and pragmatic trials. Additional extensions are forthcoming: for those and for up to date references relevant to this checklist, see www.consort-statement.org.

2017 CONSORT checklist of information to include when reporting a randomized trial assessing nonpharmacologic treatments (NPTs)*. *Modifications of the extension appear in italics and blue.*

Completed for: 'A multimodal intervention of manual therapy, exercise, and psychological management for painful diabetic neuropathy: Results of the NeuOst feasibility randomised controlled trial'

Section/Topic Item	Checklist item no.	CONSORT item	Extension for NPT trials	Reported in manuscript location
**Title and abstract**				
	1a	Identification as a randomized trial in the title		For all main CONSORT checklist items, see completed 2025 checklist.
	1b	Structured summary of trial design, methods, results, and conclusions (for specific guidance see CONSORT for abstracts)	*Refer to CONSORT extension for abstracts for NPT trials*	
**Introduction**				
Background and objectives	2a	Scientific background and explanation of rationale		
	2b	Specific objectives or hypotheses		
**Methods**				
Trial design	3a	Description of trial design (such as parallel, factorial) including allocation ratio	When applicable, how care providers were allocated to each trial group	

Section/Topic Item	Checklist item no.	CONSORT item	Extension for NPT trials	Reported in manuscript location
	3b	Important changes to methods after trial commencement (such as eligibility criteria), with reasons		
Participants	4a	Eligibility criteria for participants	When applicable, eligibility criteria for centers and for *care providers*	Methods: Eligibility criteria
	4b	Settings and locations where the data were collected		
Interventions†	5	The interventions for each group with sufficient details to allow replication, including how and when they were actually administered	Precise details of both the experimental treatment and comparator	
	5a		Description of the different components of the interventions and, when applicable, description of the procedure for tailoring the interventions to individual participants.	
	5b		Details *of whether and* how the interventions were standardized.	Methods: Intervention and comparator & Supplemental Digital Content & published intervention development article: https://doi.org/10.1080/17581869.2025.2515010

Section/Topic Item	Checklist item no.	CONSORT item	Extension for NPT trials	Reported in manuscript location
	5c.		Details *of whether and* how adherence of care providers to the protocol was assessed or enhanced	Methods: Fidelity promotion and monitoring
	5d		*Details of whether and how adherence of participants to interventions was assessed or enhanced*	Methods: Fidelity promotion and monitoring & Results: Primary analysis: Feasibility and acceptability
Outcomes	6a	Completely defined pre-specified primary and secondary outcome measures, including how and when they were assessed		
	6b	Any changes to trial outcomes after the trial commenced, with reasons		
Sample size	7a	How sample size was determined	When applicable, details of whether and how the clustering by care providers or centers was addressed	N/A (single-centre trial)
	7b	When applicable, explanation of any interim analyses and stopping guidelines		
Randomization:				
- Sequence generation	8a	Method used to generate the random allocation sequence		
	8b	Type of randomization; details of any restriction (such as blocking and block size)		

Section/Topic Item	Checklist item no.	CONSORT item	Extension for NPT trials	Reported in manuscript location
- Allocation concealment mechanism	9	Mechanism used to implement the random allocation sequence (such as sequentially numbered containers), describing any steps taken to conceal the sequence until interventions were assigned		
- Implementation	10	Who generated the random allocation sequence, who enrolled participants, and who assigned participants to interventions		
Blinding	11a	If done, who was blinded after assignment to interventions (for example, participants, care providers, those assessing outcomes) and how	~~Whether or not those administering co-interventions were blinded to group assignment~~<br>If done, who was blinded after assignment to interventions (e.g., participants, care providers, *those administering co-interventions,* those assessing outcomes) and how	Methods: Blinding (Co-interventions: N/A as none present)
	11b	If relevant, description of the similarity of interventions	~~If blinded, method of blinding and description of the similarity of interventions~~	
	11c		*If blinding was not possible, description of any attempts to limit bias*	Methods: Blinding

Section/Topic Item	Checklist item no.	CONSORT item	Extension for NPT trials	Reported in manuscript location
Statistical methods	12a	Statistical methods used to compare groups for primary and secondary outcomes	When applicable, details of whether and how the clustering by care providers or centers was addressed	N/A (single-centre trial)
	12b	Methods for additional analyses, such as subgroup analyses and adjusted analyses		
**Results**				
Participant flow (a diagram is strongly recommended)	13a	For each group, the numbers of participants who were randomly assigned, received intended treatment, and were analyzed for the primary outcome	The number of care providers or centers performing the intervention in each group and the number of patients treated by each care provider or in each center	Results: Provider characteristics; Not documented: Number of patients treated by each care provider
	13b	For each group, losses and exclusions after randomization, together with reasons		
	13c		*For each group, the delay between randomization and the initiation of the intervention*	Not documented
	new		Details of the experimental treatment and comparator as they were implemented	Interventionist fidelity in treatment delivery
Recruitment	14a	Dates defining the periods of recruitment and follow-up		
	14b	Why the trial ended or was stopped		

Section/Topic Item	Checklist item no.	CONSORT item	Extension for NPT trials	Reported in manuscript location
Baseline data	15	A table showing baseline demographic and clinical characteristics for each group	When applicable, a description of care providers (case volume, qualification, expertise, etc.) and centers (volume) in each group.	Results: Provider characteristics
Numbers analyzed	16	For each group, number of participants (denominator) included in each analysis and whether the analysis was by original assigned groups		
Outcomes and estimation	17a	For each primary and secondary outcome, results for each group, and the estimated effect size and its precision (such as 95% confidence interval)		
	17b	For binary outcomes, presentation of both absolute and relative effect sizes is recommended		
Ancillary analyses	18	Results of any other analyses performed, including subgroup analyses and adjusted analyses, distinguishing pre-specified from exploratory		
Harms	19	All important harms or unintended effects in each group (for specific guidance see CONSORT for harms)		
**Discussion**				

Section/Topic Item	Checklist item no.	CONSORT item	Extension for NPT trials	Reported in manuscript location
Limitations	20	Trial limitations, addressing sources of potential bias, imprecision, and, if relevant, multiplicity of analyses	In addition, take into account the choice of the comparator, lack of or partial blinding, and unequal expertise of care providers or centers in each group	Discussion section
Generalizability	21	Generalizability (external validity, applicability) of the trial findings	Generalizability (external validity) of the trial findings according to the intervention, comparators, patients, and care providers and centers involved in the trial	Discussion section
Interpretation	22	Interpretation consistent with results, balancing benefits and harms, and considering other relevant evidence		
**Other information**				
Registration	23	Registration number and name of trial registry		
Protocol	24	Where the full trial protocol can be accessed, if available		
Funding	25	Sources of funding and other support (such as supply of drugs), role of funders		

**Additions or modifications to the 2010 CONSORT checklist. CONSORT = Consolidated Standards of Reporting Trials*

†The items 5, 5a, 5b, 5c, 5d are consistent with the Template for Intervention Description and Replication (TIDieR) checklist

***Cite as:** Boutron I, Altman DG, Moher D, Schulz KF, Ravaud P. CONSORT Statement for Randomized Trials of Nonpharmacologic Treatments: A 2017 Update and a CONSORT Extension for Nonpharmacologic Trial Abstracts. Annals of Internal Medicine. 2017 Jul 4;167(1):40–7.*

TIDieR-Placebo Checklist with added items from the Recommendations for the Development, Implementation, and Reporting of Control Interventions in Efficacy Trials of Physical, Psychological, and Self-Management Therapies (CoPPS) statement

–

Checklist completed for the NeuOst feasibility trial.

Note: In the below table, Supplemental Digital Content 2 (SDC2) refers to the document 'Development and description of the NeuOst intervention', SDC3 refers to the document 'Development and description of the control intervention', SDC4 refers to an additional data/results supplement of the publication.

Item	Where located			Where located	
	Primary paper (page or appendix number)	Other (details)		Primary paper (page or appendix number)	Other (details)
**Active intervention**			**Control intervention**		
0 Processes of sham intervention development					For all items, also see Supplemental Digital Content (control intervention description and manual) of the feasibility RCT publication
			Sources and processes that informed the development of the control intervention.	Methods (section "Control intervention")	SDC3 (section "Control intervention rationale and development")
**1 Brief Name**					
Provide the name or a phrase that	Abstract, Introduction ("study objectives"),	For all items, also see Supplemental Digital Content	Provide the name or a phrase that describes the control intervention	Abstract, Introduction ("study objectives"),	

describes the intervention	Methods (“Intervention and comparators”)	(NeuOst intervention description and manual) of the feasibility RCT publication		Methods (“Intervention and comparators”)	
**2 Why**					
Describe any rationale, theory, or goal of the elements essential to the intervention	Methods (“NeuOst intervention”)	SDC (section “Intervention theory”)	Describe any rationale, theory, or goal of the elements essential to the control intervention*<br><br>*Theoretical considerations underlying the control intervention (including explicit mechanistic rationales and objectives of the control intervention)*	Methods (“Interventions - Control intervention”)	SDC (sections “Control intervention rationale and development” and “detailed description of control intervention”)
**3 What (materials)**					
			A highly detailed description of the content of the control intervention (covering all components listed in table 2 of the CoPPS publication and including resemblance or differences to the test intervention)	-	SDC (section “Description of control intervention” and control intervention manual)
Describe any physical or informational materials used in the intervention,	-	SDC (“Elaboration of individual programme components”)	Describe any physical or informational materials used in the control intervention, including those provided to	-	SDC (“Elaboration of individual programme components”), and SDC (“Detailed

including those provided to participants or used in intervention delivery or in training of intervention providers. Provide information on where the materials can be accessed (such as online appendix, URL)			participants or used in intervention delivery or in training of intervention providers. Provide information on where the materials can be accessed (such as an online appendix, URL)		description of control intervention”)
**4 What (procedures)**					
Describe each of the procedures, activities, and/or processes used in the intervention, including any enabling or support activities	Methods (sections “NeuOst intervention”)	SDC (“Elaboration of individual programme components”)	Describe each of the procedures, activities, and/or processes used in the control intervention, including any enabling or support activities	Methods (“Interventions - Control intervention”)	SDC (“Elaboration of individual programme components”), and SDC (“Detailed description of control intervention
**5 Who provided**					
For each category of intervention provider (such as psychologist, nursing assistant), describe their expertise, background, and any specific training given<br><br>*Should also include a description of provider behaviour, verbal and non-verbal*	Methods (“Providers and provider training”)<br><br>Results (“Provider characteristics”)	SDC (sections “provider training course” and “providers and provider training”)	For each category of control intervention provider (such as psychologist, nursing assistant), describe their expertise, background, and any specific training given<br><br>*Should also include a description of provider behaviour, verbal and non-verbal communication, and issues of equipoise as detailed in the text of the CoPPS guideline; as well*	Results (“Trial provider training and eligibility”)	SDC (sections “provider training course” and “providers and provider training”)

communication, and issues of equipoise as detailed in the text of the CoPPS guideline; as well as means to control these provider-related factors.			*as means to control these provider-related factors.*		
Additional provider-related information<br><br>*Reporting should further include how issues of equipoise and provider expectancy were addressed; and if and how provider behaviour and verbal and non-verbal communication were controlled in each group. If different sets of providers were employed to report test and control interventions, this needs to be reported along with differences in their characteristics.*	-	SDC (sections "provider training course" and "providers and provider training")	*Additional provider-related information*<br><br>*Reporting should further include how issues of equipoise and provider expectancy were addressed; and if and how provider behaviour and verbal and non-verbal communication were controlled in each group. If different sets of providers were employed to report test and control interventions, this needs to be reported along with differences in their characteristics.*	-	SDC (sections "provider training course" and "providers and provider training")
**6 How**					
Describe the modes of delivery (such as face to face or by some other mechanism, such as internet or telephone)	Methods ("NeuOst intervention")	SDC ("Intervention theory" and "Elaboration of programme components" sections)	Describe the modes of delivery (such as face to face or by some other mechanism, such as internet or telephone) of the control intervention	Results ("Interventions - Control intervention")	SDC (section "Detailed description of control intervention", and "Matched components")

of the intervention and whether it was provided individually or in a group			and whether it was provided individually or in a group		
**7 Where**					
Describe the type(s) of location(s) where the intervention occurred, including any necessary infrastructure or relevant features	Methods ("Setting")	-	Describe the type(s) of locations(s) and settings where the control intervention occurred, including any necessary infrastructure or relevant features	Results ("Study setting")	-
**8 When and how much**					
Describe the number of times the intervention was delivered and over what period of time including the number of sessions, their schedule, and their duration, intensity, or dose	Results ("Interventions")	SDC ("Intervention theory", "Elaboration of programme components", and "Treatment protocol for feasibility trial" sections)	Describe the number of times the control intervention was delivered and over what period of time including the number of sessions, their schedule, and their duration, intensity, or dose. If relevant, include the duration of the pre-, and post-randomisation consultations	Results ("Interventions")	SDC ("Control intervention protocol per trial session")
**9 Tailoring**					
If the intervention was planned to be personalised, titrated or adapted, then describe what, why, when, and how	-	SDC ("Treatment protocol for feasibility trial")	If the control intervention was planned to be personalised, titrated or adapted, then describe what, why, when, and how	-	SDC ("Detailed description of control intervention", "Matched components" and "Control intervention protocol per trial session")
**10 Modifications**					

If the intervention was modified during the course of the study, describe the changes (what, why, when, and how)	Results ("Fidelity")	-	If the control intervention was modified during the course of the study, describe the changes (what, why, when, and how)	N/A (due to protocol nature of publication)	-
**11 How well: planned**					
Planned: If intervention adherence or fidelity was assessed, describe how and by whom, and if any strategies were used to maintain or improve fidelity, describe them	Methods ("Fidelity")	Full protocol (Open Science Framework, osf.io/jyftb)	Planned: If control intervention adherence or fidelity was assessed, describe how and by whom, and if any strategies were used to maintain or improve fidelity, describe them	Results "Primary (feasibility) outcomes")	Full protocol (Open Science Framework, osf.io/jyftb)
**12 How well: actual**					
Actual: If intervention adherence or fidelity was assessed, describe the extent to which the intervention was delivered as planned	Results ("Fidelity")	SDC ("Additional feasibility and clinical data")	Actual: If control intervention adherence or fidelity was assessed, describe the extent to which the intervention was delivered as planned	-	SDC ("Additional feasibility and clinical data")
			Whether any reasons for loss to follow-up (participant attrition) during the trial were related to the control intervention.	Results ("CONSORT flow chart")	-
**13 Measuring the success of blinding**					

Was blinding measured, and if so: how, and what were the results of such measurement?<br><br>*Blinding should always be assessed if it was an objective of the control intervention, and that results should be reported as summary statistics per group, allowing independent calculation of blinding indices.*	Results ("Primary analysis")	SDC ("Additional feasibility and clinical data")	*Blinding should always be assessed if it was an objective of the control intervention, and that results should be reported as summary statistics per group, allowing independent calculation of blinding indices.*	Results ("Primary analysis")	SDC ("Additional feasibility and clinical data")
14 Assessment of participant expectation					
Report the method of assessment, timepoints, and results as summary statistics per group.	Results ("Primary analysis")	SDC ("Additional feasibility and clinical data")	*Report the method of assessment, timepoints, and results as summary statistics per group.*	Results ("Primary analysis")	SDC ("Additional feasibility and clinical data")

Reference to checklist: Hohenschurz-Schmidt, D., Vase, L., Scott, W., Annoni, M., Ajayi, O.K., Barth, J., Bennell, K., Berna, C., Bialosky, J., Braithwaite, F., Finnerup, N.B., Williams, A.C. de C., Carlino, E., Cerritelli, F., Chaibi, A., Cherkin, D., Colloca, L., Côté, P., Darnall, B.D., Evans, R., Fabre, L., Faria, V., French, S., Gerger, H., Häuser, W., Hinman, R.S., Ho, D., Janssens, T., Jensen, K., Johnston, C., Lunde, S.J., Keefe, F., Kerns, R.D., Koechlin, H., Kongsted, A., Michener, L.A., Moerman, D.E., Musial, F., Newell, D., Nicholas, M., Palermo, T.M., Palermo, S., Peerdeman, K.J., Pogatzki-Zahn, E.M., Puhl, A.A., Roberts, L., Rossettini, G., Matthiesen, S.T., Underwood, M., Vaucher, P., Vollert, J., Wartolowska, K., Weimer, K., Werner, C.P., Rice, A.S.C., Draper-Rodi, J., 2023. Recommendations for the development, implementation, and reporting of control interventions in efficacy and mechanistic trials of physical, psychological, and self-management therapies: the CoPPS Statement. BMJ 381, e072108. https://doi.org/10.1136/bmj-2022-072108

A multimodal manual therapy-based intervention for people with painful diabetic neuropathy:

Development and description of the NeuOst intervention (incl. treatment manual used during feasibility trial)

A NEW APPROACH FOR PEOPLE WITH PAINFUL DIABETIC NEUROPATHY

Authors and collaborators

David Hohenschurz-Schmidt* and the NeuOst working group

*** Chief investigator:**

Imperial College London, London, UK

University College of Osteopathy, London, UK

Version 3.0, 11 March 2024

Intervention theory of NeuOst: context and components

In the following, the context, programme theory, stakeholders, key uncertainties, and plans for intervention refinement of NeuOst will be described.

Context definition

The socio-political context of NeuOst is given by the situation of people with diabetes, and more specifically pDPN, in the UK. As a disease with strong links to socioeconomic determinants of health, type 2 diabetes has become more prevalent in the UK in the past 12 years of austerity policies (Whicher et al., 2020; Marmot, 2020). Economically, at least 10% of the National Health Service's (NHS) budget (£10 billion) is spent on diabetes, 80% of which on disease complications.

With multiple complications, including foot ulcerations, blindness, mental health problems, and neuropathic pain, diabetes causes personal suffering and has a large impact on families, carers, and society (Whicher et al., 2020).

In the NHS, diabetes patients are managed by their General Practitioners or referred to specialist services. Whilst management of type 1 diabetes relies on education, blood glucose management, physical activity, and insulin therapy, the treatment of type 2 diabetes can involve medications such as metformin. In the NHS, non-pharmacological management of type 2 diabetes focuses on dietary interventions for weight and blood glucose management, structured education, and screening for and management of disease complications (McGuire et al., 2016). The uptake of many educational and behavioural interventions is, however, poor. Importantly, accessibility of key NHS services is also suboptimal (Whicher et al., 2020).

Amongst the sequelae of diabetes is neuropathic pain (pDPN). Depression, anxiety, low quality of life, and poor sleep are associated with pain in pDPN (Kioskli et al., 2019a). Healthcare options for people with pDPN are largely limited to pharmacological therapy, notably amitriptyline, duloxetine, gabapentin or pregabalin (Tan et al., 2010). These drugs have only moderate effectiveness and can produce side effects such as drowsiness and dizziness (Finnerup et al., 2015), likely reducing their acceptability amongst patients. Non-pharmacological pain management is only provided in specialist NHS pain services, notably by psychologists, physiotherapists and specialist nurses. However, these services may not be accessible to people with pDPN because other patients are usually classed a higher priority (Annina Schmid, personal correspondence). Because of the low average income of people with (type 2) diabetes (Espelt et al., 2011), access to private services is likely limited.

Osteopaths are Allied Healthcare Professionals, trained in medical differential diagnosis, manual therapy techniques, and the conservative biopsychosocial management of people experiencing pain (Institute of Osteopathy, n.d.). This expertise, however, mainly pertains to people with musculoskeletal conditions and non-specific pain, most of whom are treated in private practice (Plunkett et al., 2021). In the UK, osteopathy is a profession regulated by the General Osteopathic Council which also defines practice standards, including the need for osteopaths to recognise whether they have the training, skills and competence to treat a particular patient (General Osteopathic Council, 2018). Taken together, the current focus of osteopathic training on musculoskeletal health and the predominance of private practice models means two things: First, additional postgraduate training may be required to enable osteopaths to safely and competently work with people with diabetes and neuropathic pain. Secondly, low-threshold access to care may require subsidised private practice models, the help of educational institutions (some of which have existing community clinics providing subsidised care), or, eventually integration into NHS pathways.

Within the above-described context, NeuOst is to be provided by specifically trained osteopaths to people with diabetes and neuropathic pain, likely in private practice or teaching institutions and over the course of multiple treatment sessions.

NeuOst can also be illustrated with a logic model, visualising the proposed theory of change (Figure 4). Importantly, the MRC framework also asks how complex interventions may be influenced by, and may themselves influence, their context. Ideally, NeuOst is hoped to alter the relationship of people with pDPN with their social and physical environment, by promoting interaction and initiative. Within the osteopathic community, NeuOst is hoped to stimulate discussion about the role of osteopaths in a post-reductionist paradigm of care, in the wider UK healthcare system but also in society at large. Clearly, the socio-political context of many people with diabetes in the UK imposes limitations on their ability to engage in treatment programmes and is not conducive to the prevention of type 2 diabetes in the first place. Therefore, questions of real-world effectiveness and generalizability to non-trail populations will have to be considered carefully at later stages of the intervention development; Conversely, interaction with this group of people may encourage osteopaths to take on advocacy roles at various levels.

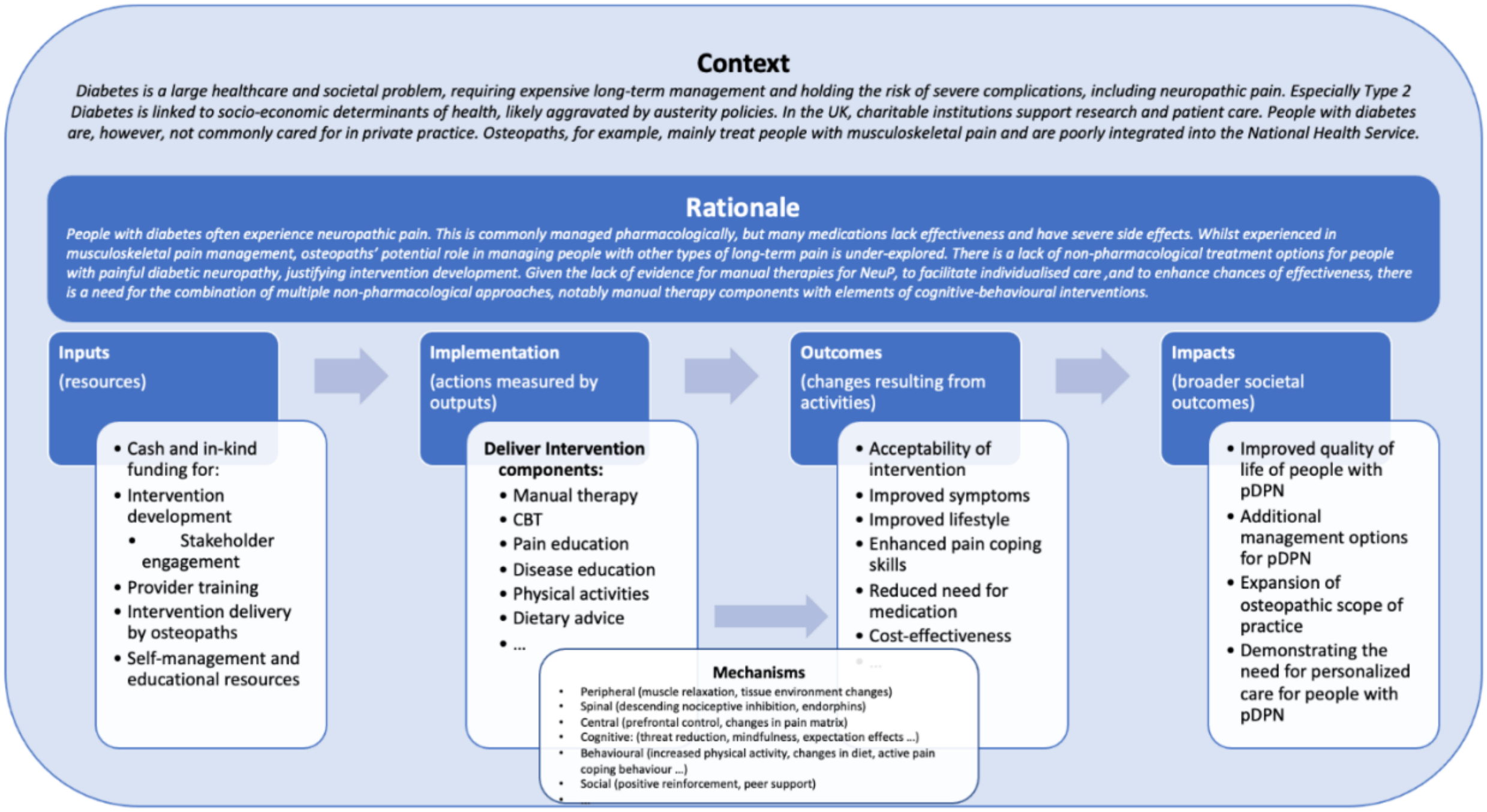

Figure 1: Logic model of NeuOst complex intervention. Created based on: https://www.gov.uk/guidance/evaluation-in-health-and-wellbeing-creating-a-logic-model & https://www.strategyunitwm.nhs.uk/sites/default/files/2018-03/Logic%20models%20and%20complex%20programmes%20-%20a%20brief%20guide.pdf

Programme theory

Programme theory describes the key components of an intervention, how an intervention is expected to have effects, as well as how intervention components are expected to interact with one another and with the intervention context (Skivington et al., 2021) (Figure 1).

The NeuOst intervention has several basic components, defined by current osteopathic practice and curricula, and informed by engagement of practicing osteopaths. Added components were chosen to 'augment' osteopathic practice with compatible techniques thought to be effective for people with pDPN (See table 1), based on involvement of subject experts, providers, and people with painful DPN in the intervention development. The basic osteopathic components include individualised biopsychosocial case history taking, manual therapy techniques for diagnosis (palpation, inspection, functional testing) and intervention (soft tissue techniques, joint and spinal articulation and manipulation), and some behavioural or pain management advice, and often anatomical education (Fawkes et al., 2009). Additional components include physical therapy and rehabilitation elements, educational components from diabetes care, and psychologically-informed techniques.

The below table describes the content of individual components of the multimodal NeuOst programme. The table also provides a description of how these components are thought to influence outcomes (mechanism) and interact with other components. It further lists existing evidence of components' efficacy / effectiveness. Importantly, the table begins to outline how individual components may be integrated into the multimodal programme. This includes considerations of how each component may be "triggered" by individual patient needs (vs. being a standard component of the programme), and processes of adapting components to patient needs (individualisation) versus needs for standardisation. During intervention development, the below table formed the main tool for subject experts to comment on programme components, and for patient and provider stakeholder preferences to be incorporated. In doing so, programme theory "*promotes a shared understanding of the intervention among diverse stakeholders*" (Skivington et al., 2021. p. 4). Once a theory of change had been agreed, the table formed the basis of a programme flowchart and decision-making algorithm, as well as the provider training that conveys NeuOst content to practicing osteopaths. In the subsequent stage, options for the evaluation of NeuOst were developed (feasibility trial protocol design) (Craig and Campbell, 2015).

Intervention components table

The below table provides an overview of potential intervention components, amenable to MSK practice, and to be combined in *NeuOst*. At the development stage, this table served as the basis for discussion amongst the research team and during stakeholder engagements.

Intervention elements can be split into manual and non-manual groups and pre-defined 'techniques' in each group (following the UK Back pain Exercise And Manipulation (UK BEAM) trial approach (Harvey et al., 2003)). During the feasibility trial, practitioners will be expected to deliver certain components in each, with some flexibility as to specific techniques and their mode of delivery, and are not allowed to deliver treatment components not specified in the protocol. ACT-informed techniques were based on the unpublished 'ACT OPEN Workbook', used to accompany online ACT treatment delivered for people with HIV-related neuropathy (Scott et al., 2021) and similar to the workbook used in Kioskli et al.'s (2020) study with people with pDPN.

Table 1: Intervention components of NeuOst. Manual therapy components and techniques based on Harvey et al. (2003) classification, with 'subtle' manual therapy components added.

Intervention Type	Intervention Components	Treatment targets / Mechanisms relevant to pDPN	Evidence of Efficacy and / or Effectiveness *(population, study design, comparator, n total, main findings)*	Potential role in Multimodal Intervention	Expected interaction with other components
**Manual therapy-based components**					
**Soft tissue components**	Massage of painful and non-symptomatic areas; deep vs superficial; painful vs pleasant techniques<br><br>(Techniques: cross-fibre stretch, longitudinal stretch, direct pressure, deep friction)	counter-pain mechanisms; overall / ANS relaxation; muscle relaxation; expectation effects	A review that included (six) manual therapy trials found Thai foot massage, Gua Sha, reflexology and aromatherapy to be more effective on pain and function parameters than education controls, TAU, or no treatment. Also included trials on CTS in people with DM (Hernández-Secorún et al., 2021). Largest trial apparently Chatchawan et al. (2015) on six sessions of Thai foot massage vs educational control (total n = 60),	List of eligible techniques that can be applied freely depending on provider choice and patient preferences.	Forming the basis of 'treatment' during in-person sessions; Hoped to facilitate engagement in more active self-management components through short-term relief.<br><br>Arguably enhancing the therapeutic relationship / alliance through touch

**Articulatory / mobilisation components**	(Techniques: low- through high-amplitude passive movements of lumbar spine and sacroiliac joints (and necessarily hips); flexion, extension, rotation, side-bending, manual traction; oscillation)		finding benefits in functional and sensation tests.<br><br>Looking at primary pain-outcomes and >3-week follow-up in neuropathies, Moisset et al. (2020) identified no eligible studies from most non-drug fields, including physiotherapy, therapeutic patient education, Tai Chi or Chinese acupuncture.<br><br>For spinal manipulation for peripheral (poly-)neuropathies, no human studies could be found. (See Song et al., (2016); Onifer et al., (2018) for rodent studies)<br><br>Here you can cite the literature on immediate neurophys effects of spinal mobilisation, see also our (now rather old Systematic review in manual therapy 2008). Mostly short term effects though, but pain modulatory plus sympathetic NS.		(McGuirk, 2012; Kerr et al., 2019).
**'Subtle' Manual Therapy components**	(Myofascial, craniosacral, and visceral techniques)<br><br>Gentle hands-on approaches, usually based on structural models of health and disease or palpation of tissue tension.	ANS relaxation; muscle relaxation; expectation effects			
**Thrust components ('manipulations')**	(Techniques: high or low velocity; low amplitude; direct or leverage; directed at central lumbar, zygapophysial or sacroiliac joints; unilateral or bilateral; at one or more locations)<br><br>Thrust force; Cavitation; joint loading and tension; sensory experience	muscle relaxation; descending pain inhibition; temporarily enhanced joint mobility; expectation effects			
**Soft tissue component 2 (Neurodynamic techniques)**	Manual techniques that aim at mobilising a nerve in its peripheral course.	Usually applied to suspected entrapment neuropathies. Role in	Single trial on "nerve mobilisation" (Singh et al., 2012): n = 30, DM2, 21 days tibial nerve stretch (Dorsiflexion	Introduced during in-person treatment sessions and provided	Potentially reinforcing benefits of manual therapy; Linking in with activity-increasing aspect

	Can also be provided as home-programme.	pDPN to be explored. Techniques for sciatic and distal nerves likely most relevant.	+ Eversion + SLR, 10 mins per day) compared to no treatment. Found sign. b/w group differences in changes in vibration thresholds. High RoB and 4/11 PEDro score according to (Hernández-Secorún et al., 2021).<br><br>In chemotherapy-induced neuropathic pain, a recent exploratory RCT suggests usefulness of upper extremity home-based programme (daily 5-10 minutes starting prior to chemotherapy) (Andersen Hammond et al., 2020)	as part of home-exercise programme	of home-exercise programme.
**Non-manual therapy-based components**					
**Physical activity / Exercise components**					
**Exercise, Sensorimotor / balance advice and prescription**	Advice and provision of exercise plans, including detail on form, sets, and repetitions, frequency, and individualisation.<br><br>Type of exercises:<br><br>**Sensorimotor / balance training**; regimen provided as training plan (see Appendix in (Ahmad et al., 2020))	Sensorimotor / balance training:<br><br>Supposed altered motor programming and movement patterning through repeated proprioceptive and somatosensory input (Ahmad et al., 2020). Fall prevention and avoidance of pressure areas on feet (risk reduction	Sensorimotor / balance training: Hernández-Secorún et al. (2021) reviewed 12 moderate-to-high-quality trials of various balance programmes in DPN, one with vibration elements. They report largely significant benefits on various proprioceptive, some neurophysiological and EMG, and few functional or disability outcomes, compared to attention or TAU controls. E.g., some indication from small attention-controlled RCT (n = 38) that proprioception, EMG, and	Could be either provided to those people who express an interest in a home-based exercise plan, or as generic take-home programme. Equipment would have to be provided or improvised or exercises adapted; Initial practice within clinic session; Safety concerns?	May interact with below advice on general activity, for example by setting the basis for starting some form of physical activity or allowing for movement even during very painful episodes, or by improving proprioception and making daily activities easier. Also interact with mindfulness and pain education components, trying to

		for ulcerations) (Hernández-Secorún et al., 2021).	potentially nerve conduction velocity may be improved (Ahmad et al., 2020). Ankle-reaching task + virtual obstacle crossing (2x per week, 4 weeks; n = 39, elderly) showed some effects on balance over no-treatment control (Grewal et al., 2015). Note on **dosage**: All studies have session schedules of at least 40-60mins once per week, usually more frequent.		reframe movement as beneficial and non-threatening. Interaction with manual components: providing additional sensory input.
**Exercise (Physical, Stretching)**	Static and / or dynamic lengthening of muscle groups of varying duration and sequences.		No studies found that examined solely stretch exercises, but stretches were included in most regimes reviewed by (Hernández-Secorún et al., 2021)	Can be provided at very low intensity and may thus be suitable for people with high levels of pain or high kinesiophobia as introductory exercises. Practiced during in-person sessions and provided as "homework".	May be facilitated through short-term relief from manual therapy and enabled through cognitive changes from CBT-based and pain education components (incl. goal setting techniques). Could be combined with elements of mindfulness or controlled breathing.
**Exercise (Physical, anaerobic / Strength)**	Strengthening exercises with body-weight, resistance bands, or weights. Variable repetitions; load parameters; addressed muscle groups; May include social components.	Autonomic; muscle strength; coordination; proprioception; endorphins	Reviewing 4 trials in DPN, all of which deemed strength training effective in balance and strength variables compared with no-treatment control groups (Hernández-Secorún et al., 2021).	Provided depending on patient preference and disability levels; Potentially practiced during in-person sessions and provided as "homework";	May be facilitated through short-term relief from manual therapy and enabled through cognitive changes from CBT-based and pain education components (incl. goal setting techniques).

**Exercise (Physical, aerobic)**	Cardiovascular exercises from fast walking to running / cycling / swimming. Variable in duration; intensity; social; whole-body vs legs only	Cardiovascular and autonomic; proprioception; endorphins; (Diabetes-related mechanisms?); Weight loss	Hernández-Secorún et al. (2021) reviewing 7 aerobic-type training programmes, finding most of them to be effective than no-treatment controls for function, pain, and balance in DPN. Positive findings for both HIIT and moderate intensity training. Also, treadmill compared favourably to balance training. No meta-analysis and primary studies not always great.	Provided depending on patient preference, access to equipment and training facilities, and disability levels.	Interactions with dietary components; May be facilitated through short-term relief from manual therapy and enabled through cognitive changes from CBT-based and pain education components (incl. goal setting techniques and value-driven action).
**Breathing exercises**	OR breathing re-training. Controlled diaphragmatic breathing, usually with specific duration of in- and outbreaths.	Stress-reduction (ANS); prefrontal control; Control over physical reaction to pain.		Potentially practiced during in-person sessions and provided as "homework"	Can be incorporated in all physical exercises and forms part of many mindfulness techniques; May increase ability to engage in movement through altered attention focus (from pain experience to breathing)
**Communication, Education, and ACT-informed components**					
**Pain self-management advice**	Advice on rest, ice, heat, over-the-counter medication, and physical activity.	Pharmacological analgesia during flare-ups; Local and central effects on nociceptive processing.		Providers have access to list of best-practice advice for MSK and neuropathic pain flare-ups. Can be provided verbally and / or via an information sheet.	May complement intervention sessions by easily applicable, active, and healthy coping strategies. Basis of more specific exercise / regular engagement in activities. Links with pain education to understand value of active coping strategies and rationale for pharmacological pain

					relief in facilitating movement and (possibly) preventing chronification.
**General physical activity guidance**	Recommendations on starting or modifying physical activities. May include daily walking, taking stairs, interrupt sedentary periods, or exercise programmes such as gym, yoga, swimming, cycling, speed walking running, etc.; Could consider use of accelometre / pedometric app.<br><br>Technique: Can be delivered in 5A's manner (Estabrooks et al., 2003), a structured communication approach to evaluate and promote physical activity in primary care.	- slow muscle atrophy<br>- maintain gait mechanics<br>- reduce risk of falls<br>- cardiovascular benefits and enhanced glycaemic control<br><br>Statement of the American Diabetes Accoc.: "*Exercise improves blood glucose control in type 2 diabetes, reduces cardiovascular risk factors, contributes to weight loss, and improves well-being. Regular exercise may prevent or delay type 2 diabetes development. Regular exercise also has considerable health benefits for people with type 1 diabetes (e.g.,*	Activity promotion studied often alongside dietary intervention in diabetes risk prevention (Balk et al., 2015). Recent 3-armed RCT found little benefit on metabolic function from pedometer-based activity promotion, with or without additional group support for behaviour change and diabetes specialist 1:1 support, and any effects waning after intervention termination (Rossen et al., 2021). Reviews conclude that pedometers increase activity levels, with less evidence to suggest metabolic improvements (Baskerville et al., 2017; Franssen et al., 2020), and some indication that combination with in-person consultation may add benefit (Hodkinson et al., 2021).	Aim could be to build on patient's preferences and existing habits. Needs to be individualised; "Advice" would be provided using below CBT-informed communication strategies.	Hoped to be enhanced through osteopaths' training in CBT-informed techniques.

		improved cardiovascular fitness, muscle strength, insulin sensitivity, etc.).” Should be tailored to individual needs (Colberg et al., 2016)			
**Mindfulness-based Stress Reduction, MBSR**	Meditation, Rest, Breathing …	Stress-reduction (ANS); prefrontal control; empowerment	High-intensity 8-week group programme improved function, better health-related quality of life, and reduced pain intensity, pain catastrophizing, and depression compared to those receiving usual care (individual RCT, pDPN, n = 66) (Nathan et al., 2017).	Self-management component; Instructed through provider / other resources	
**Education (Pain Education)**	Learning about pain mechanisms; experiential and individualised components (incl. reflection on own experience); Neuropathic pain-related information	Reducing kinesiophobia and catastrophizing. Possibly reframing threat value of pain.	Systematic review finding improvements in kinesophobia and catastrophising but not pain intensity or quality of life (Watson et al., 2019). Also see (Ashar et al., 2021) regarding these cognitive mediators.	As part of provider interaction at outset (to set the scene for improved pain self-management); As (optional) component provided through external resources (online)	Possibly allowing for better engagement in physical activity and / or manual therapy through reductions in negative pain connotations.
**Sleep Management (Education)**	Learning about best-practice strategies to improve sleep	Addressing one of the most salient QoL disturbances (as identified during PPI and congruent with literature (Ferini-Strambi, 2017; Naranjo et al., 2019))	…	Provided as simple hand-out and discussed with provider	Possibly buffering the impact of NeuP on sleep and addressing relevant effect of symptom; thereby making intervention more logical and hopefully effective.

**Acceptance and Comittment Therapy (ACT) – informed components**	Techniques / exercises (from Scott & McCracken, n.d., ACT OPEN workbook), selection:<br><br>1. Focus on your breathing (Mindfulness)<br>2. Passenger on the bus Metaphor and reflective questions (Shifting attentional focus)<br>3. Notice five things (Awareness)<br>4. Unwanted party guest Metaphor and reflective questions (Openness)<br>5. Observe-Breathe-Open Up Meditation (combination of skills)<br>6. Walking Meditation	Mechanisms:<br><br>Learning to tolerate distressing experience and to learn self-acceptance (Lau and McMain, 2005)<br><br>More control over emotional aspect of pain experience	Moisset et al. (2020) included five open-label RCTs of psychological studies in their review on pain in (any) peripheral neuropathy, concluding: There is a weak recommendation for the use of CBT and mindfulness as an add-on therapy for neuropathic pain (moderate final quality of evidence).<br><br>Another review (Racaru et al., 2021) included nine RCTs with both TAU and attention control comparators. Meta-analyses of 2-4 studies each showed significant effects of interventions over controls for short- and medium-term pain intensity, pain interference, and depressive symptoms, and for short-term quality of life.	Several:<br>1. Combined mindfulness-based exercises during manual treatment<br>2. Dedicated mindfulness exercises during treatment session, such as body scans etc.<br>3. Homework-based practice possible for all (if programme inflexible) / some patients (if individualised)<br><br>In training programme:<br><br>Cultivate accepting and mindful provider state of mind (provider practice?)	Promoting an 'aware mode' characterised by a freedom of choice (Lau and McMain, 2005), possibly allowing for increased activity and engagement despite pain, fear of pain, or associated negative thoughts.<br><br>May interact with pain and psychoeducation, "about [pain]-related thoughts and symptoms can facilitate earlier detection of these experiences, thereby increasing the chance of timely interventions like a previously created relapse-prevention plan." (Lau and McMain, 2005), p. 865
**Relaxation (PMR)**	Guided sequence of contraction and relaxation of subsequent muscle groups.	Stress-reduction (ANS)	(Izgu et al., 2020) 3-arm parallel-group trial of 12-week self-managed PMR vs mindfulness vs single-session educational attention control (T2DM with NeuP, n total = 77), finding sign. b/w-group differences in favour of PMR and MM over control for pain intensity but not QoL.	Step-by-step protocol provided as audio-file onto patient's phone (see Izgu et al. supplement). Could be standard component or according to patient preference.	

**Mindfulness meditation**	Guided mindfulness tasks involving sustained awareness of bodily sensations, breathing, etc.	Stress-reduction (ANS); prefrontal control;	(Izgu et al., 2020), see above.<br><br>Evidence synthesis of *brief* mindfulness-based interventions on pain-related outcomes is inconclusive (McClintock et al., 2019). Longer programmes seem not to have consistent effects on pain, QoL, or mental health in acute or chronic pain (Ball et al., 2017); Effects on physical function in chronic pain are questionable (Jackson et al., 2019). No evidence of effects from n=20 pilot by Teixeira (2010): After 4-week pre-recorded meditation programme compared to nutrition diary for neuropathic pain, NP-related quality of life, and sleep quality.		Mindfulness elements could also be integrated into manual and exercise-based intervention components. Refocus of attention from pain experience to, eg, breathing may facilitate engagement in physical activity despite pain. Supported by positive effects of pain education and CBT / goal setting.
**Diabetes management components**					
**Diabetes complication screening and management**	Providers trained to routinely screen for diabetes complications and doing so at regular intervals.<br><br>Providers trained to recognise hypo and deliver hypo treatment in emergency situations	Prevention of complications and / or aggravation.	Part of NICE guideline for Type 2 Diabetes (NICE, 2015). Further guidelines exist for individual complications.	Incorporated into baseline assessments and regular check-ups.	Hoped to enhance therapeutic relationship through professionalism and reduce risk of disease complications alongside more active components. Comply with best-practice guidelines; role as first contact practitioner; Raising patients' awareness to signs and

					symptoms of potential complications (linked to education components).
**Education (Diabetes and its complications)**	Providers trained in pathophysiological knowledge and clinical presentations;		Systematic review of 42 RCTs finding that self-management education reduces all-cause mortality risk compared to usual care (He et al., 2017)	Could be short checking in with patient regarding their disease understanding and addressing of misconceptions if present	Basic knowledge to facilitate communication and management, and enhance therapeutic relationship.
**Dietary components**					
**Dietary (Advice or Planning)**	Intervention could range from simple encouragement to change eating habits, to online education session or peer-support group, to individualised dietary plans.			Basic checking of dietary habits and existing management strategies; qualified comment or further advice only if needed. Could also be (optional) online or group-based component.	Similar to above, but could play larger role and be easier / more relevant to deliver
**Integration with standard medical care**					
**Integration with pharmacological and other medical DM management**	Acknowledgement of medication list; Provider awareness of common side effects and prescribing guidelines. Communication with prescribing clinician if side effects suspected and desired by patient.	Potential removal of side-effect-producing medication.		Part of initial consultation. Need to acknowledge professional boundaries and competence of osteopaths. Medication changes could be considered as exploratory outcome measure in feasibility	Hoped to enhance patients' trust in provider competency as well as ability to communicate with other medical professionals if needed. Knowledge of common medications and their potential side-effects relates to education and choice of self-

	Providers aware of standard medical management, incl. medications and side effects.<br><br>Providers know the standard clinical steps / stages of patient management and can communicate with respective specialists.			study, will have to be monitored as potential confounder / mechanisms during definite trials.	management advice; Pain relief from other sources maybe allowing for reduction in medication.
**Integration with pharmacological and other medical <u>pain</u> management**					
**Excluded elements**					
	**Justification**				
**High-velocity thrusts to the neck**<br><br>(Cervical manipulation with a rotary component, taking the neck beyond its normal range**)**	To limit any risk of serious adverse effects and because not indicated for pDPN				
**Printed or digital materials outside the dedicated NeuOst material**	To reduce heterogeneity during a trial and avoid possible contradiction				

	with NeuOst management approach				
**Conservative treatment modalities occasionally provided by osteopaths**: Acupuncture, bed rest, biofeedback, electrotherapy.	To reduce heterogeneity during a trial and avoid possible contradiction with NeuOst management approach				
**Appliances and devices**: Belts, Straps, Braces, Splints, Tape, Massage guns, Laser and Ultrasound, Digital Therapeutics, Wellness smartphone or PC applications. (**Not excluded** is NeuP-relevant advice on e.g., compression gloves or socks, heated or low-sensory mittens, wrist splints for Carpal Tunnel Syndrome, desensitization and stereognosis exercises (Andersen Hammond et al., 2020))	To reduce heterogeneity during a trial and avoid possible contradiction with NeuOst management approach. To maintain pragmatic treatment provision				

Intervention elements per treatment session (treatment protocol for feasibility trial)

The intervention components will be delivered in a step-wise programme, delivered over five sessions. This approach was chosen to provide practitioners with an initial structure to deliver NeuOst. It will also facilitate high-fidelity intervention delivery during the feasibility trial. Clinically, graded exposure to physical activity, a step-by-step approach to psychological interventions, and repeated sessions of manual therapy are also common.

Briefly, target mechanisms of the NeuOst intervention are (detailed in above table):

- Behaviour change towards more physical activity
- Lower extremity muscle strength and sensorimotor control
- Cardiovascular fitness and downstream glucose metabolism
- Behaviour change towards more effective disease and complications management
- Cognitive-emotional changes towards more psychological flexibility and reduction of unhelpful pain beliefs
- Lower extremity joint mobility, soft tissue relaxation, and sensory input
- Autonomic nervous system control / stress reduction

**Intervention content** (providers decide the sequence per session)<br><br>*Components marked 'optional' are non-obligatory; all others must be delivered as part of the respective treatment session.*	**Fidelity checklist** (to be completed by trial providers)	**Notes** (Further detail such as amount and duration or provider experiences such as problems and opportunities)
**Session 1**		Session duration up to 90 mins
Communication elements:<br>**1. Active listening to patient narrative**<br>**2. Initial case history**<br>**3. Introduction to the therapeutic objectives and structure of NeuOst**<br>**4. Acknowledgement of medication list**<br>5. Conversation about medications (optional)<br>6. Communication with primary care / specialist care provider (optional)<br>7. Signposting to DM/NeuP-related care options (optional)<br>8. Exploration of patient beliefs (regarding pain and disease) (optional)<br>9. Education about pain and neuropathic pain, including management strategies, sleep management, and devices (optional)<br>ACT-informed elements:<br>**1. Exploration of currently employed pain control strategies** (via Session 1 ACT worksheet)<br>**2. Exploration of what matters to patient** (via Session 1 ACT worksheet)<br>**3. Explorations of "away" and "toward moves"** (via Session 1 ACT worksheet)<br>4. **Exploration of physcial activities and connection to what is important to patient**	Comms:<br>1. ☐<br>2. ☐<br>3. ☐<br>4. ☐<br>5. ☐<br>6. ☐<br>7. ☐<br>8. ☐<br>9. ☐<br>ACT:<br>1. ☐<br>2. ☐<br>3. ☐<br>4. ☐	Up to 30 mins
Manual therapy:<br>**1. Assessment** (inspection, active & passive joint movement, palpation)<br>**2. Peripheral joint articulation** (feet & ankles at least 2mins per side to end range-of-motion; then knees & hips any duration)<br>3. Other peripheral joint articulation (optional)<br>4. Spinal joint articulation (optional)<br>5. Spinal joint manipulation, except cervical spine (optional)<br>**6. Peripheral soft tissue manipulation** (gastrocnemius at least 1min per side)<br>7. Other peripheral soft tissue manipulation (optional)<br>8. Paraspinal soft tissue manipulation (optional)<br>9. **Active resisted muscle activation** (lower extremity: dorsiflexion, plantarflexion, knee flexion and knee extension, at least 10 times each per side, hold 5 seconds per rep.)<br>10. **Neurodynamic technique lower extremity** (active; 10 reps per side)<br>11. 'Subtle' osteopathic techniques (optional)	MT:<br>1. ☐<br>2. ☐<br>3. ☐<br>4. ☐<br>5. ☐<br>6. ☐<br>7. ☐<br>8. ☐<br>9. ☐<br>10. ☐<br>11. ☐<br>12. ☐	Up to 20 mins

12. Integration of patient breathing, relaxation, interoceptive awareness ("notice" and "open"), and/or values- and activity-exploration (ACT-based) into manual treatment (optional)		
<u>Neurological</u>:<br>1. **Provider baseline neurological examination** (in addition to research-related baseline testing; not used as outcome)	Neuro:<br>1. ☐	Up to 10 mins
<u>Physical activity</u>:<br>**1. Raise the topic of physical activity with permission (use motivational interviewing techniques throughout)** (5As': "Ask")<br>**2. Evaluation of current physical activity levels** ("Assess")<br>**3. Explore barriers to more engagement in physical activity**<br>**4. Educate about benefits of different types of physical activity** ("Advise")<br>**5. Provide "Exercise and diabetes" handout** (by the Diabetes Research & Wellness Foundation) ("Advise")<br>**6. Values-based goal setting for aerobic exercise activity** (patient choice of activity, use Borg scale for exertion level) ("Agree")<br>**7. Practice session 1 home-based sensorimotor, stretch, strength (SSS) programme content** (have patient go through all exercises and reps each time!)<br>8. Decide on suitable modifications for SSS programme (optional)<br>**9. Provide exercise plan handout / link** ("Arrange")<br>10. **Provide "wobble cushion"** (provided as part of trial)	Exx:<br>1. ☐<br>2. ☐<br>3. ☐<br>4. ☐<br>5. ☐<br>6. ☐<br>7. ☐<br>8. ☐<br>9. ☐<br>10. ☐	Up to 20 mins
<u>Diabetes related</u>:<br>**1. Diabetes complication screening** (red flags for foot health, vision changes, kidney disease etc.)<br>2. Conversation about dietary DM management (optional)<br>3. Education about DM (optional)<br>4. Education about DPN and other complications of DM, including management strategies (optional)<br>5. Provision of NeuOst educational materials (optional)	DM/DPN:<br>1. ☐<br>2. ☐<br>3. ☐<br>4. ☐<br>5. ☐	Up to 10 mins
Home programme content:<br><br>*Cardio:*<br>(Duration or distance, and level of exhaustion agreed in session and documented on handout; grade difficulty using Borg Rating Scale of Perceived Exertion to achieve "somewhat hard" to "hard" physical activity [13-15/20])<br><br>1. 3x p.w.: Engage in agreed amount of aerobic activity<br><br>*SSS (sensorimotor, stretch, strength) exercises:*<br><br>(Possible modification: support with handhold on desk etc if needed – safety; Eyes open vs. closed for difficulty; With or without wobble board)<br><br>2. 2x p.d.: 10x slump 'tensioner' neurodynamic movement<br>3. 1x p.d.: 10x sit-to-stand<br>4. 30-60 secs: Wobble cushion exercise (modifiable from simple two-legged standing to single-leg reaching)		

Also see next page!

PROVIDERS TO COMPLETE AT END OF SESSION:

Please rate your agreement with the following statement with regards to today's intervention session for the management of the patient's painful diabetic neuropathy symptoms.

This treatment will be completely effective.

Strongly disagree	Moderately disagree	Slightly disagree	Neither agree nor disagree	Slightly agree	Moderately agree	Strongly agree

**Session 2**		45-60 mins duration
<u>Communication</u>:<br>**1. Active listening to patient narrative**<br>**2. Follow-up case history**<br>3. Conversation about medications (optional)<br>4. Communication with primary care / specialist care provider (optional)<br>5. Signposting to DM/NeuP-related care options (optional)<br>6. Exploration of patient beliefs (regarding pain and disease) (optional)<br>7. Education about pain and neuropathic pain, including management strategies, sleep management, and devices (optional)<br><u>ACT-informed elements</u>:<br>**1. Revisiting last week's exploration of what matters to patient and away/towards moves**<br>**2. Exploring the problem with control via "Beach ball metaphor"** (via Session 2 ACT worksheet)<br>**3. "Notice 5 things" exercise** (via Session 2 ACT worksheet)<br>**4. Encouragement to practice awareness exercise at home**	1. ☐<br>2. ☐<br>3. ☐<br>4. ☐<br>5. ☐<br>6. ☐<br>7. ☐<br><br>1. ☐<br>2. ☐<br>3. ☐<br>4. ☐	Up to 20 mins
<u>Manual therapy</u>:<br>**1. Assessment** (inspection, active & passive joint movement, palpation)<br>**2. Peripheral joint articulation** (feet & ankles at least 2mins per side to end range-of-motion; then knees & hips any duration)<br>3. Other peripheral joint articulation (optional)<br>4. Spinal joint articulation (optional)<br>5. Spinal joint manipulation, except cervical spine (optional)<br>**6. Peripheral soft tissue manipulation** (gastrocnemius at least 1min per side)<br>7. Other peripheral soft tissue manipulation (optional)<br>8. Paraspinal soft tissue manipulation (optional)<br>**9. Active resisted muscle activation** (lower extremity: dorsiflexion, plantarflexion, knee flexion and knee extension, at least 10 times each per side, hold 5 seconds per rep.)<br>**10. Neurodynamic technique lower extremity** (active; 10 reps per side)<br>11. 'Subtle' osteopathic techniques (optional)<br>12. Integration of patient breathing, relaxation, interoceptive awareness ("notice" and "open"), and/or values- and activity-exploration (ACT-based) into manual treatment (optional)	1. ☐<br>2. ☐<br>3. ☐<br>4. ☐<br>5. ☐<br>6. ☐<br>7. ☐<br>8. ☐<br>9. ☐<br>10. ☐<br>11. ☐<br>12. ☐	Up to 20 mins
<u>Neurological</u>:<br>1. Follow-up neurological examination (if indicated, optional)	1. ☐	
<u>Physical activity</u>:<br>**1. Revisit experiences and/or exercises of SSS programme**<br>**2. Explore if exercises and stretches can now be performed at greater difficulty level or through larger range of movement**<br>3. Reinforce importance of exercise (optional)<br>4. Explore barriers to adherence and provide advice on overcoming those (optional)<br>**5. Practice session 2 home-based sensorimotor, stretch, strength (SSS) programme content** (have patient go through all exercises and reps each time!)<br>6. Decide on suitable modifications for SSS programme (optional)<br>**7. Ensure patient has access to exercise plan** ("Arrange")	1. ☐<br>2. ☐<br>3. ☐<br>4. ☐<br>5. ☐<br>6. ☐<br>7. ☐	Up to 10 mins

Diabetes management and education:<br>**1. Foot care education and practical run-through** (under guidance of below handout)<br>**2. Provide foot care handout** (“Diabetes and loking after your feet” from Diabetes UK)<br>3. Conversation about dietary DM management (optional)<br>4. Education about DM (optional)<br>5. Education about DPN and other complications of DM, including management strategies (optional)<br>6. Provision of other NeuOst educational materials (optional)	1. ☐<br>2. ☐<br>3. ☐<br>4. ☐<br>5. ☐<br>6. ☐	Up to 10 mins
**Home programme content Week 2:**<br>*Cardio:*<br>1. 3x p.w.: Engage in agreed amount of aerobic activity<br>*SSS (sensorimotor, stretch, strength) exercises:*<br>2. 2x p.d.: 10x slump ‘tenser’ neurodynamic movement<br>3. 1x p.d.: 10x sit-to-stand<br>4. 30-60 secs: Wobble cushion exercise (modifiable from simple two-legged standing to single-leg reaching)<br>**5. 1x p.d.: 10x heel-to-toe raises (new)**<br>**6. 1x p.d.: 10x per side: Standing knee raises or Step-up-and-down on stairs (new)**		

PROVIDERS TO COMPLETE AT END OF SESSION:

Please rate your agreement with the following statement with regards to today’s intervention session for the management of the patient’s painful diabetic neuropathy symptoms.

This treatment will be completely effective.

Strongly disagree	Moderately disagree	Slightly disagree	Neither agree nor disagree	Slightly agree	Moderately agree	Strongly agree

**Session 3**		45-60 mins duration
Communication:<br>**1. Active listening to patient narrative**<br>**2. Follow-up case history**<br>3. Conversation about medications (optional)<br>4. Communication with primary care / specialist care provider (optional)<br>5. Signposting to DM/NeuP-related care options (optional)<br>6. Exploration of patient beliefs (regarding pain and disease) (optional)<br>7. Education about pain and neuropathic pain, including management strategies, sleep management, and devices (optional)<br><u>ACT-informed elements</u>:<br>**1. Reviewing patient experiences with "Noticing 5 things" exercise**<br>**2. Providing information on values vs goals** (via Session 3 ACT worksheet)<br>**3. Completing "Values Compass" and setting SMART goal** (Session 3 ACT worksheet)	1. ☐<br>2. ☐<br>3. ☐<br>4. ☐<br>5. ☐<br>6. ☐<br>7. ☐<br><br>1. ☐<br>2. ☐<br>3. ☐	Up to 30 mins
<u>Manual therapy</u>:<br>**1. Assessment** (inspection, active & passive joint movement, palpation)<br>**2. Peripheral joint articulation** (feet & ankles at least 2mins per side to end range-of-motion; then knees & hips any duration)<br>3. Other peripheral joint articulation (optional)<br>4. Spinal joint articulation (optional)<br>5. Spinal joint manipulation, except cervical spine (optional)<br>**6. Peripheral soft tissue manipulation** (gastrocnemius at least 1min per side)<br>7. Other peripheral soft tissue manipulation (optional)<br>8. Paraspinal soft tissue manipulation (optional)<br>**9. Active resisted muscle activation** (lower extremity: dorsiflexion, plantarflexion, knee flexion and knee extension, at least 10 times each per side, hold 5 seconds per rep.)<br>**10. Neurodynamic technique lower extremity** (active; 10 reps per side)<br>11. 'Subtle' osteopathic techniques (optional)<br>12. Integration of patient breathing, relaxation, interoceptive awareness ("notice" and "open"), and/or values- and activity-exploration (ACT-based) into manual treatment (optional)	1. ☐<br>2. ☐<br>3. ☐<br>4. ☐<br>5. ☐<br>6. ☐<br>7. ☐<br>8. ☐<br>9. ☐<br>10. ☐<br>11. ☐<br>12. ☐	Up to 20 mins
<u>Neurological</u>:<br>1. Follow-up neurological examination (if indicated, optional)	1. ☐	
<u>Physical activity</u>:<br>**1. Revisit experiences and/or exercises of SSS programme**<br>**2. Explore if exercises and stretches can now be performed at greater difficulty level or through larger range of movement**<br>3. Reinforce importance of exercise (optional)<br>4. Explore barriers to adherence and provide advice on overcoming those (optional)<br>**5. Practice session 3 home-based sensorimotor, stretch, strength (SSS) programme content** (have patient go through all exercises and reps each time!)<br>6. Decide on suitable modifications for SSS programme (optional)<br>**7. Ensure patient has access to exercise plan ("Arrange")**	1. ☐<br>2. ☐<br>3. ☐<br>4. ☐<br>5. ☐<br>6. ☐<br>7. ☐	Up to 10 mins
<u>Diabetes management and education</u>:		Up to 10 mins

**1. Revisit content and experiences with foot care education and handout**<br>2. Conversation about dietary DM management (optional)<br>3. Education about DM (optional)<br>4. Education about DPN and other complications of DM, including management strategies (optional)<br>5. Provision of other NeuOst educational materials (optional)	1. ☐<br>2. ☐<br>3. ☐<br>4. ☐<br>5. ☐	
**Home programme content Week 3:**<br><br>*Cardio:*<br>1. 3x p.w.: Engage in agreed amount of aerobic activity<br>*SSS (sensorimotor, stretch, strength) exercises:*<br>2. 2x p.d.: 10x slump 'tenser' neurodynamic movement<br>3. 1x p.d.: 10x sit-to-stand<br>4. 30-60 secs: Wobble cushion exercise (modifiable from simple two-legged standing to single-leg reaching)<br>5. 1x p.d.: 10x heel-to-toe raises<br>6. 1x p.d.: 10x per side: Standing knee raises or Step-up-and-down on stairs<br>**7. 1x p.d.: 10x: Kneeling lunges or Getting-up-from-floor, with or without support (new)**		

PROVIDERS TO COMPLETE AT END OF SESSION:

Please rate your agreement with the following statement with regards to today's intervention session for the management of the patient's painful diabetic neuropathy symptoms.

This treatment will be completely effective.

Strongly disagree	Moderately disagree	Slightly disagree	Neither agree nor disagree	Slightly agree	Moderately agree	Strongly agree

**Session 4**		45-60 mins duration
Communication:<br>**1. Active listening to patient narrative**<br>**2. Follow-up case history**<br>3. Conversation about medications (optional)<br>4. Communication with primary care / specialist care provider (optional)<br>5. Signposting to DM/NeuP-related care options (optional)<br>6. Exploration of patient beliefs (regarding pain and disease) (optional)<br>7. Education about pain and neuropathic pain, including management strategies, sleep management, and devices (optional)<br>ACT-informed elements:<br>**1. Revisiting last week's SMART goal** (incl. any progress or barriers)<br>**2. Practicing "opening up" skill via "Leaves on a stream" exercise** (via Session 3 ACT worksheet)<br>**3. Encouragement to practice "openness" exercises at home**	1. ☐<br>2. ☐<br>3. ☐<br>4. ☐<br>5. ☐<br>6. ☐<br>7. ☐<br><br>1. ☐<br>2. ☐<br>3. ☐	Up to 20 mins
Manual therapy:<br>**1. Assessment** (inspection, active & passive joint movement, palpation)<br>**2. Peripheral joint articulation** (feet & ankles at least 2mins per side to end range-of-motion; then knees & hips any duration)<br>3. Other peripheral joint articulation (optional)<br>4. Spinal joint articulation (optional)<br>5. Spinal joint manipulation, except cervical spine (optional)<br>**6. Peripheral soft tissue manipulation** (gastrocnemius at least 1min per side)<br>7. Other peripheral soft tissue manipulation (optional)<br>8. Paraspinal soft tissue manipulation (optional)<br>9. **Active resisted muscle activation** (lower extremity: dorsiflexion, plantarflexion, knee flexion and knee extension, at least 10 times each per side, hold 5 seconds per rep.)<br>10. **Neurodynamic technique lower extremity** (active; 10 reps per side)<br>11. 'Subtle' osteopathic techniques (optional)<br>12. Integration of patient breathing, relaxation, interoceptive awareness ("notice" and "open"), and/or values- and activity-exploration (ACT-based) into manual treatment (optional)	1. ☐<br>2. ☐<br>3. ☐<br>4. ☐<br>5. ☐<br>6. ☐<br>7. ☐<br>8. ☐<br>9. ☐<br>10. ☐<br>11. ☐<br>12. ☐	Up to 20 mins
Neurological:<br>1. Follow-up neurological examination (if indicated, optional)	1. ☐	
Physical activity:<br>**1. Revisit experiences and/or exercises of SSS programme**<br>**2. Explore if exercises and stretches can now be performed at greater difficulty level or through larger range of movement**<br>3. Reinforce importance of exercise (optional)<br>4. Explore barriers to adherence and provide advice on overcoming those (optional)<br>**5. Practice session 4 home-based sensorimotor, stretch, strength (SSS) programme content** (have patient go through all exercises and reps each time!)<br>6. Decide on suitable modifications for SSS programme (optional)	1. ☐<br>2. ☐<br>3. ☐<br>4. ☐<br>5. ☐<br>6. ☐<br>7. ☐	Up to 10 mins

7. **Ensure patient has access to exercise plan (“Arrange”)**		
<u>Diabetes management and education</u>:<br>1. Conversation about dietary DM management (optional)<br>2. Education about DM (optional)<br>3. Education about DPN and other complications of DM, including management strategies (optional)<br>4. Provision of other NeuOst educational materials (optional)	1. ☐<br>2. ☐<br>3. ☐<br>4. ☐	Up to 10 mins
**Home programme content Week 4:**<br><br>*Cardio:*<br>1. 3x p.w.: Engage in agreed amount of aerobic activity<br>*SSS (sensorimotor, stretch, strength) exercises:*<br>2. 2x p.d.: 10x slump ‘tenser’ neurodynamic movement<br>3. 1x p.d.: 10x sit-to-stand<br>4. 30-60 secs: Wobble cushion exercise (modifiable from simple two-legged standing to single-leg reaching)<br>5. 1x p.d.: 10x heel-to-toe raises<br>6. 1x p.d.: 10x per side: Standing knee raises or Step-up-and-down on stairs<br>7. 1x p.d.: 10x: Kneeling lunges or Getting-up-from-floor, with or without support<br>**8. 1x p.d.: 10x per side: Star-steps (front, side, back large steps) (new)**		

PROVIDERS TO COMPLETE AT END OF SESSION:

Please rate your agreement with the following statement with regards to today’s intervention session for the management of the patient’s painful diabetic neuropathy symptoms.

This treatment will be completely effective.

Strongly disagree	Moderately disagree	Slightly disagree	Neither agree nor disagree	Slightly agree	Moderately agree	Strongly agree

Session 5		45-60 mins duration
Communication:<br>**1. Active listening to patient narrative**<br>**2. Follow-up case history**<br>3. Conversation about medications (optional)<br>4. Communication with primary care / specialist care provider (optional)<br>5. Signposting to DM/NeuP-related care options (optional)<br>6. Exploration of patient beliefs (regarding pain and disease) (optional)<br>7. Education about pain and neuropathic pain, including management strategies, sleep management, and devices (optional)<br>ACT-informed elements:<br>**1. Exploring experiences with practicing "Leaves on a stream" since last session**<br>**2. Reviewing any skills practice patient may be performing** (incl. impact on life, opportunities for further practice, barriers and how to manage those moving forward)<br>**3. Review session 3 SMART goal** (incl. any progress towards that)<br>**4. Discussing how to deal with difficulties**	1. ☐<br>2. ☐<br>3. ☐<br>4. ☐<br>5. ☐<br>6. ☐<br>7. ☐<br><br>1. ☐<br>2. ☐<br>3. ☐<br>4. ☐	Up to 20 mins
Manual therapy:<br>**1. Assessment** (inspection, active & passive joint movement, palpation)<br>**2. Peripheral joint articulation** (feet & ankles at least 2mins per side to end range-of-motion; then knees & hips any duration)<br>3. Other peripheral joint articulation (optional)<br>4. Spinal joint articulation (optional)<br>5. Spinal joint manipulation, except cervical spine (optional)<br>**6. Peripheral soft tissue manipulation** (gastrocnemius at least 1min per side)<br>7. Other peripheral soft tissue manipulation (optional)<br>8. Paraspinal soft tissue manipulation (optional)<br>9. **Active resisted muscle activation** (lower extremity: dorsiflexion, plantarflexion, knee flexion and knee extension, at least 10 times each per side, hold 5 seconds per rep.)<br>10. **Neurodynamic technique lower extremity** (active; 10 reps per side)<br>11. 'Subtle' osteopathic techniques (optional)<br>12. Integration of patient breathing, relaxation, interoceptive awareness ("notice" and "open"), and/or values- and activity-exploration (ACT-based) into manual treatment (optional)	1. ☐<br>2. ☐<br>3. ☐<br>4. ☐<br>5. ☐<br>6. ☐<br>7. ☐<br>8. ☐<br>9. ☐<br>10. ☐<br>11. ☐<br>12. ☐	Up to 20 mins
Neurological:<br>**1. Follow-up neurological examination (this week not optional)** (for providers; not used as outcome during research study)	1. ☐	Up to 15 mins
Physical activity:<br>**1. Revisit experiences and/or exercises of SSS programme**<br>**2. Explore if exercises and stretches can now be performed at greater difficulty level or through larger range of movement**<br>3. Reinforce importance of exercise (optional)<br>4. Explore barriers to adherence and provide advice on overcoming those (optional)<br>**5. Practice previous sessions' home-based sensorimotor, stretch, strength (SSS) programme content** (have patient go through all exercises and reps each time!)<br>6. Decide on suitable modifications for SSS programme (optional)	1. ☐<br>2. ☐<br>3. ☐<br>4. ☐<br>5. ☐<br>6. ☐<br>7. ☐	Up to 10 mins

<table>
<tr><td>7. Ensure patient has access to exercise plan (“Arrange”)</td><td></td><td></td></tr>
<tr><td><u>Diabetes management and education</u>:
1. Explore patient’s plans for future DM and painful DPN management
2. Conversation about dietary DM management (optional)
3. Education about DM (optional)
4. Education about DPN and other complications of DM, including management strategies (optional)
5. Provision of other NeuOst educational materials (optional)</td><td>1. ☐
2. ☐
3. ☐
4. ☐
5. ☐</td><td>Up to 10 mins</td></tr>
<tr><td>Home programme content Week 5: (same as previous week)

Cardio:
1. 3x p.w.: Engage in agreed amount of aerobic activity
SSS (sensorimotor, stretch, strength) exercises:
2. 2x p.d.: 10x slump ‘tenser’ neurodynamic movement
3. 1x p.d.: 10x sit-to-stand
4. 30-60 secs: Wobble cushion exercise (modifiable from simple two-legged standing to single-leg reaching)
5. 1x p.d.: 10x heel-to-toe raises
6. 1x p.d.: 10x per side: Standing knee raises or Step-up-and-down on stairs
7. 1x p.d.: 10x: Kneeling lunges or Getting-up-from-floor, with or without support
8. 1x p.d.: 10x per side: Star-steps (front, side, back large steps)</td><td></td><td></td></tr>
<tr><td colspan="3">PROVIDERS TO COMPLETE AT END OF SESSION:

Please rate your agreement with the following statement with regards to today’s intervention session for the management of the patient’s painful diabetic neuropathy symptoms.

This treatment will be completely effective.

<table>
<tr><td>Strongly disagree</td><td>Moderately disagree</td><td>Slightly disagree</td><td>Neither agree nor disagree</td><td>Slightly agree</td><td>Moderately agree</td><td>Strongly agree</td></tr>
<tr><td></td><td></td><td></td><td></td><td></td><td></td><td></td></tr>
</table>
</td></tr>
<tr><td>End of programme</td><td></td><td></td></tr>
</table>

Elaboration of individual programme components

This section summarises the take-home messages from intervention table, with justification and brief overview of NeuOst content.

Manual therapy

Massage, spinal manipulation, joint mobilisation, and other forms of manual therapy are poorly studied in people with painful diabetic neuropathy, or even peripheral neuropathies in general. Hernández-Secorún et al. (2021) summarised the current evidence as follows: "*As for manual therapy, there seems to be a short-term effect of the different types of massage after treatment in comparison with standard-care and treatment-free control groups, with high methodological quality of the studies*." (p. 27-28). Whilst suggesting some effectiveness, it needs to be noted that this enthusiastic assessment is based on a heterogeneous sample of non-sham controlled small-to-medium-sized RCTs. For some forms of manual therapy, such as spinal manipulation, evidence relevant to peripheral neuropathies is lacking entirely. For others, such as neurodynamic stretching, it is based on individual trials only. Therefore, manual therapy elements included in NeuOst are only partly based on scientific evidence. Mainly, they are informed by involvement of osteopathic practitioners who have experience treating people with pDPN.

Manual elements of NeuOst include:

- Global joint **mobilization** sequences ("General osteopathic treatment", GOT)
- Specific joint mobilization of lower extremity joints, notably ankle and foot joints
- Passive muscle **stretching** ("Muscle energy technique", MET)
- **Spinal manipulation** of lumbar and thoracic spinal segments
- **Neurodynamic** passive stretching
- **Soft tissue** techniques (incl. cross-fibre massage, longitudinal stretch, direct pressure / trigger point inhibition, deep friction)
- **'Subtle'** manual therapy techniques such as craniosacral or myofascial approaches
- **Active-resisted muscle strengthening** (active resistance programme, see image below)

Links to other NeuOst components:

- Mindfulness elements / biofeedback approach to passive manual therapy
- Integration with breathing exercises
- Transition to self-directed strengthening and stretching physical activity elements through active resistance and passive stretching elements

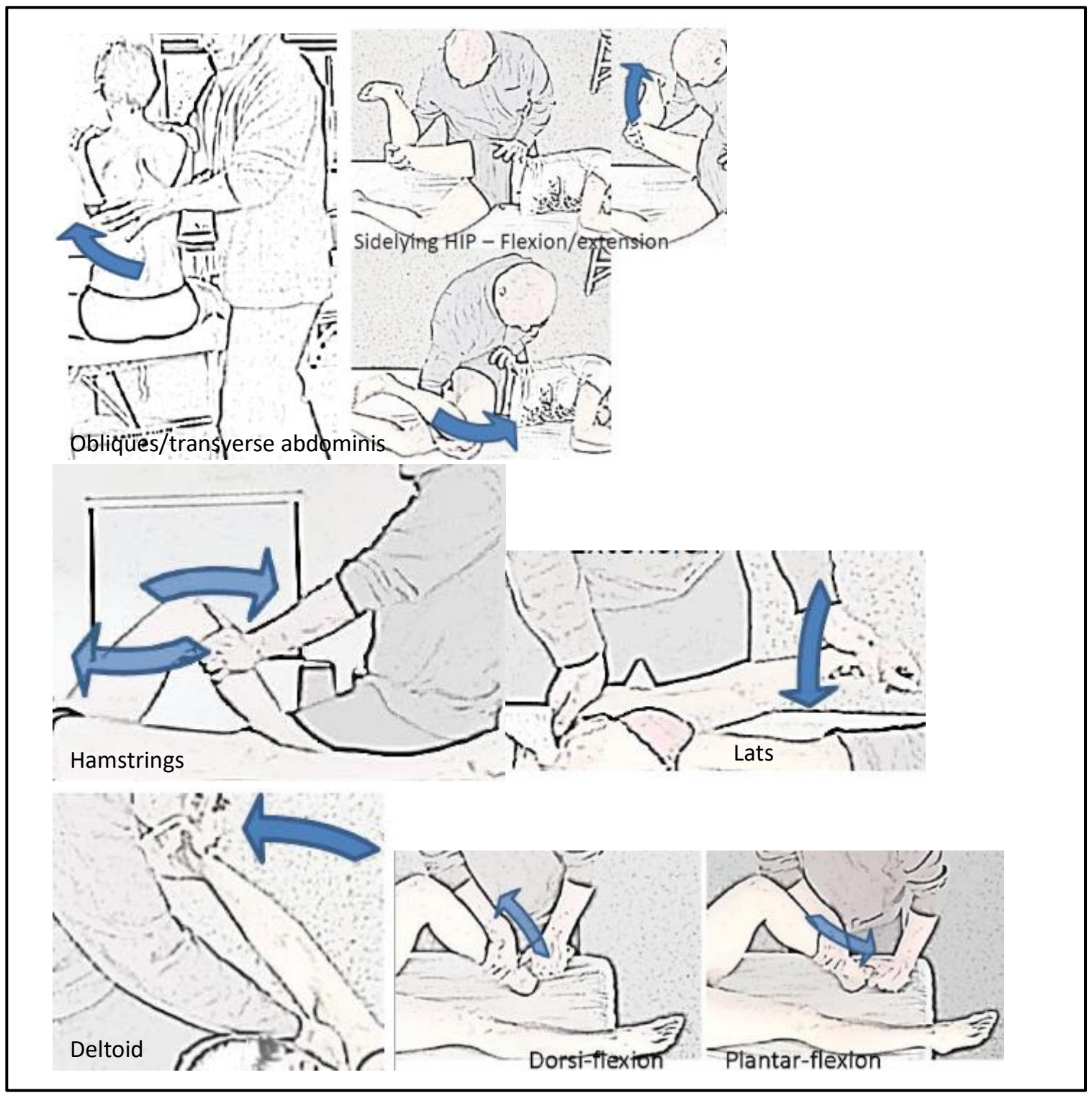

Figure 2: Active resistance programme. Exemplary indication of procedure principles and activities, and not exhaustive. In each movement, resistance is provided by the practitioner at a level to allow the patient to generate 5-6 RPE on the Borg scale.

Physical activity and exercise

A statement of the American Diabetes Association holds that "*[e]xercise improves blood glucose control in type 2 diabetes, reduces cardiovascular risk factors, contributes to weight loss, and improves well-being. Regular exercise may prevent or delay type 2 diabetes development. Regular exercise also has considerable health benefits for people with type 1 diabetes (e.g., improved cardiovascular fitness, muscle strength, insulin sensitivity, etc.).*" In their document, the ADA lists a large range of metabolic changes and clinical benefits of increased physical activity relevant for blood sugar control and reduced risk of diabetes complications. The authors further highlight that any activity programme should be tailored to individual needs (Colberg et al., 2016). The broad range of benefits for people with diabetes justifies the addressing of people's activity levels in any multimodal programme; In the subgroup of people with painful diabetic neuropathy, most trials focus on improving balance through sensorimotor and other training, with some evidence to suggest effectiveness. Evidence of effectiveness on pain and nerve function is limited. Effects of any physical activity on disease markers of diabetes are as relevant in programmes with people with neuropathy as for people without. However, a patient's

ability to engage in exercise programmes may be limited due to pain, balance problems, or foot complications.

Mechanistically, patients' prevalent pathophysiological changes, and associated symptoms or functional limitations, ought to guide intervention development. PPI and existing literature also point to the fear of falling as a major concern, requiring the possibility to adapt the training programme for safety and comfort.

Therefore, NeuOst includes exercise components that can be adapted to individual patients' abilities, preferences, and access to equipment / facilities:

- General **education, advice, and encouragement of any physical activity**, with the opportunity to individualise behavioural change techniques and specifics of recommended activity
- Training programme for paced **aerobic** activities performed at home
- Standardised and progressive training programme for home-exercise, focusing on **sensorimotor** exercises, **strengthening**, and including **neurodynamic and general stretching**
- Provision of a 'wobble cushion' to facilitate balancing exercises and enable the addition of balance elements to all anaerobic exercises

The physical activity components will follow a pacing approach, adding intensity / duration and new exercises each week. It is hoped that patients can retain the principles of pacing for their activities after the termination of the study intervention. Decisions are made in a shared manner between patient and provider, the latter of which will also draw on ACT-informed communication and techniques throughout.

E.g. for balance programme:

I. Ahmad et al.: Sensorimotor and gait training in diabetic peripheral neuropathy

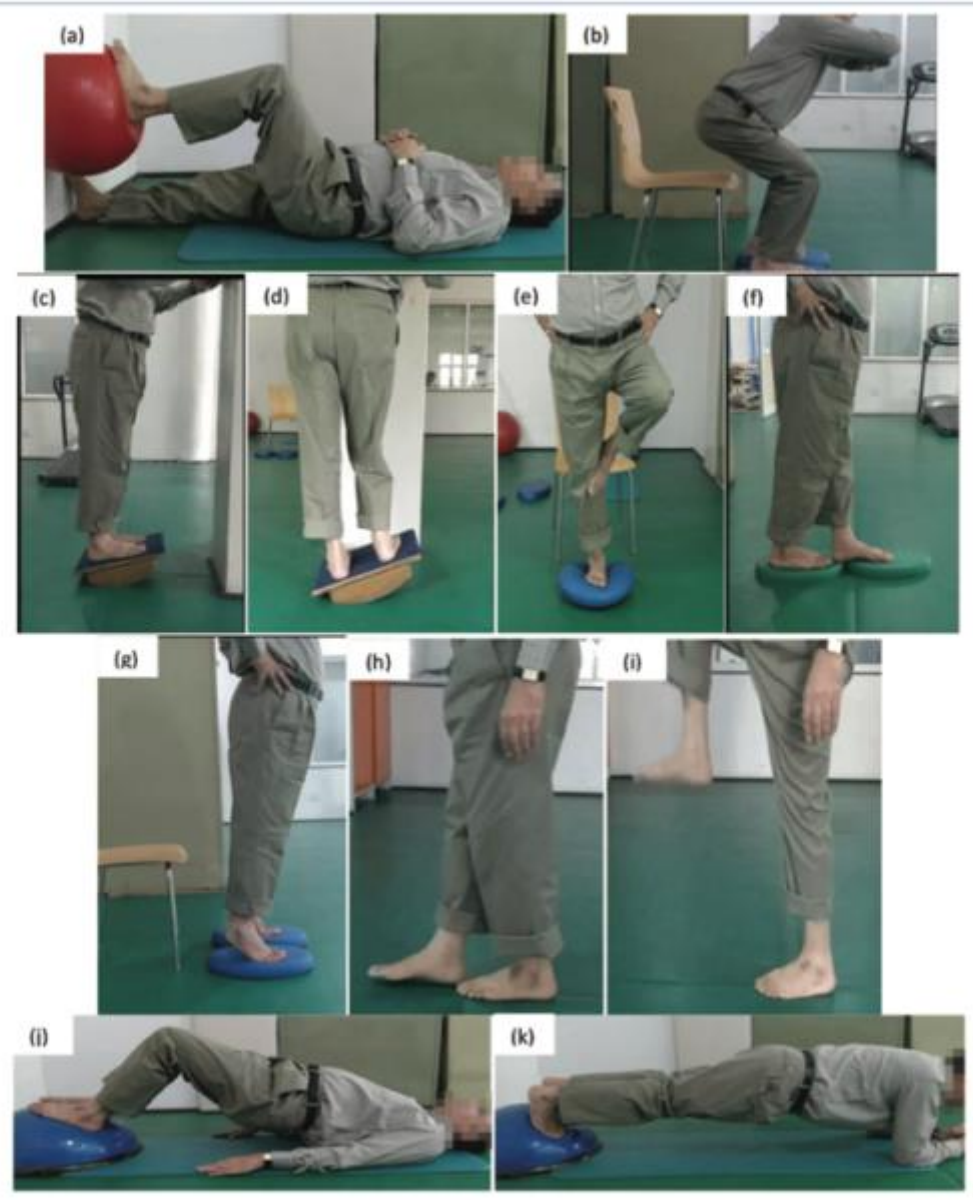

Appendix 3. Sensorimotor and gait exercises. *Sensorimotor exercises: (a) Wall slides with ball; (b) Sit to stand; (c) and (d) Bipedal wobble exercise; (e) Unipedal stance; (f) Tandem stance; (g) Toe and heel raise; Gait training: (h) Tandem walk (i) High march walk; Core training: (j) Back bridging on bosu ball (k) Prone bridging on bosu ball.*

NeuOst

YOUR NeuOst TRAINING PROGRAMME TO STRENGTHEN, STRETCH, STABILIZE, AND GET FIT

	WEEK 1	WEEK 2	WEEK 3	WEEK 4	WEEK 5	EACH WEEK, REVIEW
SSS EXERCISE	Try to do your exercises every day of the week!			Star-steps (front, side, back large steps)<br>1x per day: 10x per side	Star-steps (front, side, back large steps)<br>1x per day: 10x per side	Can you make larger steps?
			Kneeling lunges or Getting-up-from-floor, with or without support<br>1x per day: 10x	Kneeling lunges or Getting-up-from-floor, with or without support<br>1x per day: 10x	Kneeling lunges or Getting-up-from-floor, with or without support<br>1x per day: 10x	Can you use less support?
		Standing knee raises or Step-up-and-down on stairs<br>1x per day: 10x per side	Standing knee raises or Step-up-and-down on stairs<br>1x per day: 10x per side	Standing knee raises or Step-up-and-down on stairs<br>1x per day: 10x per side	Standing knee raises or Step-up-and-down on stairs<br>1x per day: 10x per side	Can you do more repetitions or rounds?
		Heel-to-toe raises, with or without support, option: on wobble board<br>1x per day: 10x	Heel-to-toe raises, with or without support or wobble board<br>1x per day: 10x	Heel-to-toe raises, with or without support or wobble board<br>1x per day: 10x	Heel-to-toe raises, with or without support or wobble board<br>1x per day: 10x	Can you use less support, or use the wobble board?
	Slump 'tenser' neurodynamic movement<br>2x per day: 10x	Slump 'tenser' neurodynamic movement<br>2x per day: 10x	Slump 'tenser' neurodynamic movement<br>2x per day: 10x	Slump 'tenser' neurodynamic movement<br>2x per day: 10x	Slump 'tenser' neurodynamic movement<br>2x per day: 10x	Can you perform this exercise through a larger range of motion?
	Sit-to-stand, option: on wobble board<br>1x per day: 10x	Sit-to-stand, option: on wobble board<br>1x per day: 10x	Sit-to-stand, option: on wobble board<br>1x per day: 10x	Sit-to-stand, option: on wobble board<br>1x per day: 10x	Sit-to-stand, option: on wobble board<br>1x per day: 10x	Can you do more repetitions or rounds, or use the wobble board?
	Wobble cushion exercise (modifiable from simple two-legged standing to single-leg reaching)<br>1x per day: 30 60 secs	Wobble cushion exercise (modifiable from simple two-legged standing to single-leg reaching)<br>1x per day: 30 60 secs	Wobble cushion exercise (modifiable from simple two-legged standing to single-leg reaching)<br>1x per day: 30 60 secs	Wobble cushion exercise (modifiable from simple two-legged standing to single-leg reaching)<br>1x per day: 30 60 secs	Wobble cushion exercise (modifiable from simple two-legged standing to single-leg reaching)<br>1x per day: 30 60 secs	Can you use less support, raise a leg briefly, or do this exercise on one leg only?
CARDIO	Do the amount and intensity of cardio exercise that was agreed with your NeuOst provider!<br>3x per week	Do the amount of cardio that was agreed with your NeuOst provider!<br>3x per week	Do the amount of cardio that was agreed with your NeuOst provider!<br>3x per week	Do the amount of cardio that was agreed with your NeuOst provider!<br>3x per week	Do the amount of cardio that was agreed with your NeuOst provider!<br>3x per week	Can you do a little bit more cardio or go a little bit faster?
						Now continue the good work of the previous weeks by following the routine from week 5.

EXERTION SCALE FOR CARDIO EXERCISES

	GREEN		YELLOW		ORANGE		RED
6	No exertion at all.	9	Very light (easy walking slowly at a comfortable peace).	13	Somewhat hard (it's quite an effort; you feel tired but can continue).	17	Very hard (very strenuous, and you are very fatigued).
7	Extremely light.	10	This is the effort level where you can't hear your breathing.	14	You start to hear your breathing, not gasping for air.	18	Breathing is vigorous. You can't talk, you're gasping for air.
8		11	You're able to easily talk and you can run here for a very long time.	15	You can talk, but more challenging, use one- or two- word answers.	19	EXTREMELY HARD | You're counting the minutes until it ends.
		12	LIGHT.	16	HARD	20	Maximal exertion.

NeuOst Strengthen, Stretch and Stabilise Training Programme – **Overview of Activities**

**Wobble cushion exercise**, modifiable from simple two-legged standing to single-leg reaching. 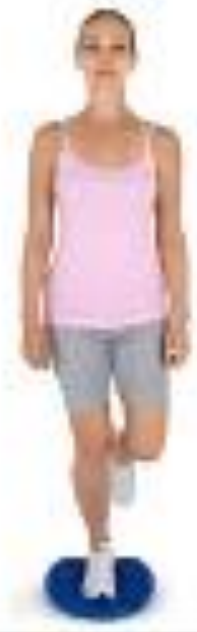	**Sit-to-stand**, option: on wobble board. 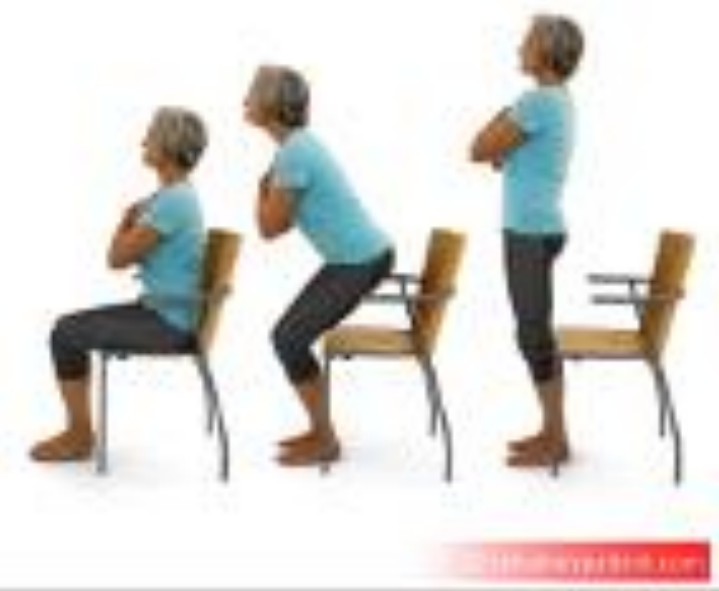
**Slump 'tenser' neurodynamic movement** 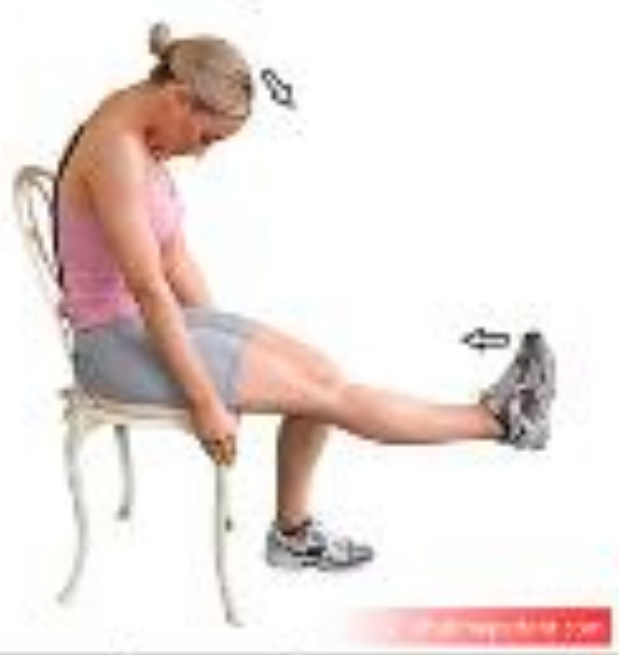	**Heel-to-toe raises**, with or without support, option: on wobble board. 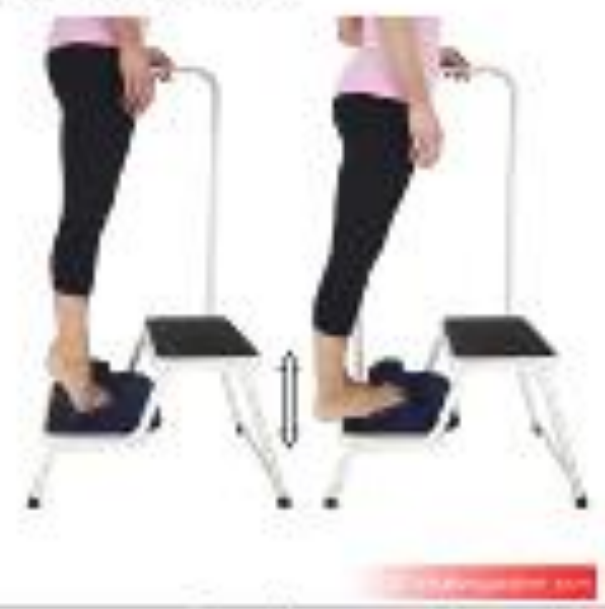
**Standing knee raises** or **Step-up-and-down on stairs** 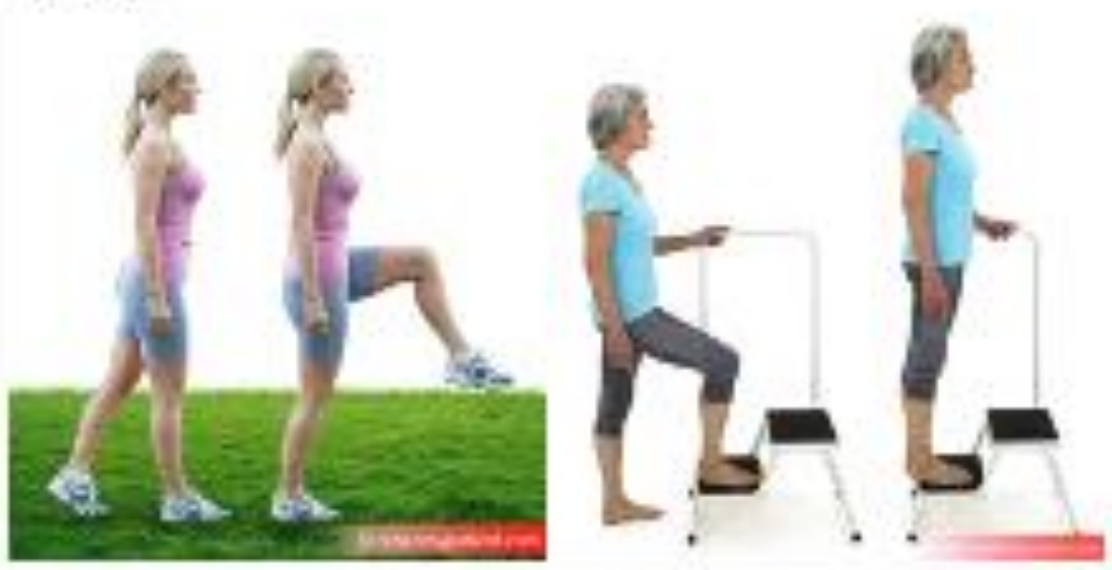	**Kneeling lunges** or Getting-up-from-floor, with or without support 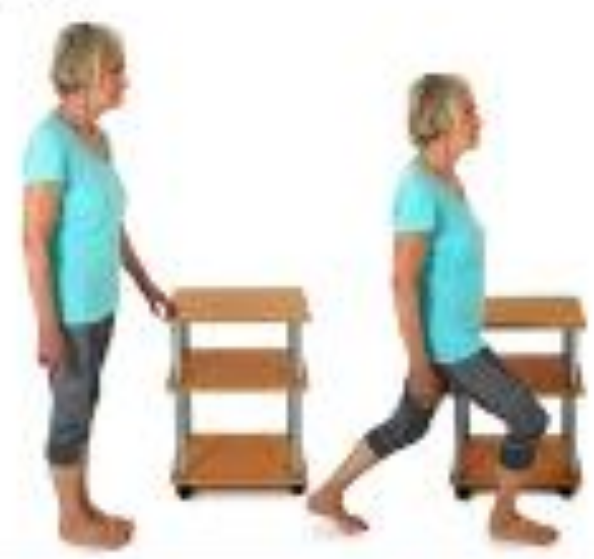
**Star-steps** (front, side, back large steps) 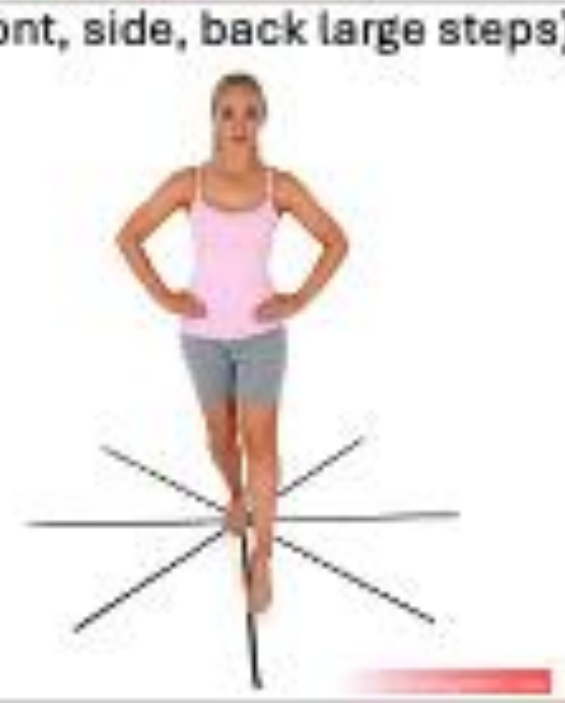	

(image soruces: Rehab My Patient ®)

Educational components for patients

In the context of NeuOst, several forms of patient education are relevant: General education on disease (diabetes) self-management and risk reduction is an integral part of guidelines and is supported by good evidence. Secondly, people with persistent pain may benefit from education about pain mechanisms (with little evidence for neuropathy-specific populations) and pain-related self-management strategies. Further potentially relevant informational components include dietary advice, sleep hygiene, ACT-informed concepts, and stress management.

Primarily, NeuOst providers will have to be able to integrate with the messaging patients receive from diabetes specialists such as nurses and doctors. Providers should be able to reinforce and support such messaging if needed. Secondly, osteopaths will have to be able to confidently answer questions related to pain mechanisms, self-management strategies, diet, sleep, and stress.

The patient-centred educational content of NeuOst will be delivered through providers in the treatment sessions, potentially supported by provision of educational material (leaflets / video links) and reinforced by discussions with providers during the in-person treatment sessions. Two leaflets will be provided to all patients prior to pre-defined treatments.

Core educational leaflets:

- "Exercise and diabetes" (Diabetes Research & Wellness Foundation)
- "Diabetes and loking after your feet" (Diabetes UK)

Optional educational videos / leaflets:

- Diet
- Sleep management
- Stress management
- Diabetes education, self-management, and risk reduction

Psychological components

The rationales of psychological interventions in the context of persistent pain vary, including the addressing of negative thoughts and emotions around pain, promoting relaxation and mindful awareness, fostering self-efficacy, and facilitating behavioural change. The goal of many cognitive-behavioural interventions is to help people manage pain, distress and disability (Williams et al., 2020). In Acceptance and Commitment Therapy (ACT), a mechanism is to increase 'psychological flexibility', a concept comprising of psychological skills such as being acceptant, present, and engaging in value-driven actions (McCracken and Morley, 2014). Pain reduction is not commonly a direct aim of psychological interventions but may result from upstream mechanisms.

Further, persistent pain is often associated with other psychological problems (Ref) and so is pDPN (Kioskli et al., 2019a), making it relevant to screen for these concomitant presentations and include them into the management (namely depression, anxiety, and worse sleep and quality of life).

In a diabetic neuropathy population, behaviour change is relevant both for more effective diabetes management (which is heavily reliant on blood sugar control and thus diet, lifestyle change, and medication compliance), as well as for neuropathy-related aspects. The latter include engagement in beneficial activities for pain management as well as activities for risk reduction. Here, regular and conscientious foot care plays a major role.

For NeuOst, providers can be equipped with a) an understanding of psychological problems and cognitive processes potentially involved when living with persistent pain, and b) psychologically-informed tools to acknowledge these processes in their patient management. Further, providers need to be able to screen for psychiatric disease potentially requiring referral.

We draw on input from expert collaborators, work around psychologically-informed physiotherapy (Keefe et al., 2018), communication skills training, and experience from previous 'augmentations' of osteopathy with cognitive-behavioural techniques (Carnes et al., 2017). Overall, the NeuOst approach is to incorporate patient attitudes, beliefs, and emotional responses into patient management through deliberate provider actions, whilst facilitating the use simple cognitive-behavioural and other psychological skills by patients. Clinically, the aim is to reduce the impact of psychological barriers on well-being, quality of life, and beneficial value-determined behaviour.

NeuOst providers will be introduced to the psychological impact and psychotherapeutic concepts during a 1-hour lecture. They will be encouraged to engage in ACT-informed reflective exercises and mindfulness-based relaxation training themselves prior to

attending the in-person training. At the in-person provider training event, a practitioner experienced in psychological interventions or psychologically-informed practice will facilitate communication training and engaging patients in ACT-informed techniques, including through role play. Providers will make further experience with the integration of mindfulness approaches into hands-on manual therapy sessions, and engage in reflective exercises themselves.

Supervision

Supervision through experienced providers plays an important role in psychotherapy training. Capacities for supervision during the NeuOst provider training phase are limited, but there will be intermittent supervision opportunities during the feasibility trial. A precedent for this approach is given by the Physical Therapy Informed by Acceptance and Commitment Therapy (PACT) study, where physiotherapists had monthly group supervision and authors commented that this “seems to have provide good support (…) to deliver PACT with high fidelity” (Godfrey et al., 2016, 2020).

Provider training course

V 1.0, 08 March 2024 (final version for training course as delivered in January 2024)

Authors: Dr David Hohenschurz-Schmidt, Steve Vogel

Course dates: 27 January 2024, 9am until end, University College of Osteopathy, Borough High Street

Delivery mode: 1 day in person + Asynchronous e-learning materials to be provided beforehand + live e-learning with trial providers after in-person training day.

Course Aims

This course aims to equip fully qualified UK osteopaths with the knowledge and practical skills to work with people living with painful diabetic neuropathy, and to do so safely and in accordance with the NeuOst intervention theory.

Further, this course aims to prepare participants as intervention providers on the NeuOst feasibility study.

Intended Learning Outcomes

The overall intended learning outcomes (ILOs) of the provider training are as follows. Each individual lesson will define learning objectives that align with the broader ILOs of the course.

At the end of the NeuOst training course, providers will:

1. Understand disease mechanisms, pathophysiological changes, complications, and clinical presentations of Diabetes Mellitus (DM), Diabetic Polyneuropathy (DPN), and painful DPN (pDPN).
2. Understand current medical options and pathways for people with pDPN in the UK.
3. Appreciate the patient perspective, and the psycho-emotional and social impact of pDPN.
4. Be able to perform and interpret a bedside neurological examination specific for people with pDPN as well as complication screening for diabetic foot disease and complications.
5. Know the conceptual basics of Acceptance and Commitment therapy (ACT), be able to draw on simple ACT-based techniques in their communication and introduce patients to ACT-informed self-management techniques.
6. Have a plan for possible manual therapy techniques and their potential rationale when working with people with pDPN. -

7. Understand the benefits of exercise in pDPN and appreciate ways of guiding patients in a pDPN-focused graded exercise programme.
8. Be able to educate patients about the basics of DM, diabetes management, pain mechanisms in pDPN, pain self-management, foot care, and DM complication screening and management.
9. Appreciate the importance of maintaining blinding and balancing of contextual factors in clinical research and be able to implement the NeuOst feasibility trial's test and control intervention with high fidelity and conviction.

Assessment

Whether course participants have achieved each session's learning objectives will be assessed by means of multiple-choice assessments after the training course and through observations of practical exercises during the course

Course plan

**Pre-course materials (asynchronous e-learning)**	**In-person 1 (synchronous)**
<u>Introduction to the NeuOst project and CPD course</u><br><br>Speaker: Dr David Hohenschurz-Schmidt<br>Duration: 25 mins<br>Delivery mode: Recorded.<br>Learning objectives:<br>• Familiarise yourself with the NeuOst project, including its objectives, underlying intervention development processes, the involved multidisciplinary team, and the basic design of the NeuOst feasibility trial.<br>• Learn about the learning outcomes, content, and delivery mode of the NeuOst training course.<br>Content:<br>• Brief overview presentation	<u>Introduction to the Practical Training Day</u><br><br>Speaker: Dr David Hohenschurz-Schmidt<br>Duration: 10 mins<br>Delivery mode: Face-to-face<br>Learning objectives:<br>• Reacquaint yourself with the Intended Learning Outcomes of the NeuOst course.<br>• Learn about the practical procedures of the in-person day.<br>Content:<br>• Brief overview presentation of plan on the day<br>• Fire safety<br>• Q&A
Theory Lesson 1:<br><br><u>Diabetes pathophysiology, risks, and medical management</u><br><br>Speaker: Dr David Hohenschurz-Schmidt<br>Duration: 1 hour<br>Delivery mode: Recorded<br>Learning objectives:<br>• Gain a comprehensive understanding of the epidemiology, pathogenesis and pathophysiological changes associated with Diabetes Mellitus (DM)<br>• Relate DM pathophysiology to common disease risks.<br>• Appreciate medical management and risk screening procedures for T2DM, as aligned with current UK NICE guidelines.<br>Content:<br>• DM basics<br>• Epidemiology and disease burden<br>• Diagnosis<br>• Risk factors and risk screening<br>• Pathophysiology (T1, T2, Prediabetes)<br>• Acute and chronic complications<br>• T2DM treatment (NICE guidance & nutrition)<br>• NHS management<br>• T2DM prevention	Practical Lesson 1:<br><br><u>Expert Q&A Session: Managing Patients with Diabetes and Diabetic Foot Disease</u><br><br>Speaker: *This session was not held in the course's iteration on 27 Jan 2024 due to unsuccessful speaker recruitment. The overall course validity was not deemed to have been affected due to the largely experiential learning objectives (objective no. 3) and having covered objectives 1 and 2 in theoretical sessions and through Practical Lesson 4.*<br><br>Duration: 0.5 hours<br>Delivery mode: Face-to-face / Speaker via videocall<br>Learning objectives:<br>• Appreciate the clinical complexity of diabetes and its complications.<br>• Understand the current best-practice pathways and their implementation in the NHS.<br>• Engage with the clinician's perspective on diabetes management.<br>Content:<br>• Semi-structured conversation and Q&A with the speaker.

• Future directions<br>• Resources	
Theory Lesson 2:<br><br><u>Diabetic Polyneuropathy</u><br><br>Speaker: Sasha Smith<br>Duration: 1 hour<br>Delivery mode: Recorded<br>Learning objectives:<br>• Understand the epidemiology and pathophysiology of DPN.<br>• Recognize clinical presentations and complications.<br>• Appreciate diagnostic criteria and methods.<br>• Explore medical management and evidence of effectiveness.<br>Contents:<br>• Terminology<br>• Prevalence<br>• Risk factors<br>• Cellular mechanisms<br>• Pathophysiological changes<br>• Symptoms<br>• Complications<br>• Diagnosis<br>• Management	Theory Lesson 8:<br><br><u>An Brief Introduction to Acceptance and Commitment Therapy (ACT) for Persistent Pain and its Application within NeuOst</u><br><br>Speaker: Dr Whitney Scott<br>Duration: 1 hour<br>Delivery mode: face-to-face<br>Learning objectives (TBC):<br>• Understand key aspects of the psychological flexibility model and the focus within ACT.<br>• Experience and practice ACT-based methods that will be delivered within NeuOst.<br>• Support reflective practice and noticing one's own responses and behaviours as a clinician.<br>Content:<br>• Psychosocial elements of living with persistent pain<br>• Principles of ACT<br>• Psychological risk screening and decision- and referral-processes
Theory Lesson 3:<br><br><u>Neuropathic Pain in Painful Diabetic Neuropathy</u><br><br>Speaker: Dr Harriet Kemp<br>Duration: 30 mins<br>Delivery mode: Recorded<br><br>Learning objectives:<br>• Understand the epidemiology and pathophysiology of neuropathic pain (NeuP) in general and painful DPN in particular.<br>• Recognize clinical presentations and complications of pDPN.<br>• Appreciate diagnostic criteria and methods.<br>• Explore medical management and evidence of effectiveness.<br>Contents:<br>• Categorisation of Pain<br>• Epidemiology<br>• Physiology of pain & painful DPN<br>• Clinical Presentation<br>• Diagnostic Criteria	Practical Lesson 2<br><br><u>An Brief Practical Introduction to ACT for Persistent Pain and its Application within NeuOst</u><br><br>Speaker: Dr Whitney Scott<br>Duration: 1 hour<br>Delivery mode: Face-to-face<br>Learning objectives:<br>• Understand key aspects of the psychological flexibility model and the focus within ACT.<br>• Experience and practice ACT-based methods that will be delivered within NeuOst.<br>• Support reflective practice and noticing one's own responses and behaviours as a clinician.<br>Content:<br>• Practical exercises of ACT-based methods.

<table>
<tr><td>• Medical Management</td><td></td></tr>
<tr><td>Theory Lesson 4

<u>Physical Activity and Exercise for People with Diabetes and Neuropathy</u>

Talk 1: The Role of Physical Activity and Exercise in the Management of Diabetes and DPN

Speaker: Dr David Hohenschurz-Schmidt
Duration: 0.75 hour
Delivery mode: Recorded
Learning objectives:
• Appreciate neurophysiological and metabolic mechanisms through which physical activity and exercise provides benefits in Diabetes (DM) and Diabetic Neuropathy (DPN).
• Understand the current evidence and official recommendations for various types of physical activity.
• Appreciate the challenges of exercise prescription in people living with painful DPN.
• Learn about the exercise component of the NeuOst programme.
Content:
• Principles of physical activity and therapeutic exercise
• Exercise in DM
• Exercise for DPN and neuropathic pain
• Challenges of exercise prescription in pDPN
• The NeuOst exercise component

Talk 2: Principles of Exercise Adherence

Speaker: Dr Daniel Bailey
Duration: 0.5 hour
Delivery mode: Recorded
Learning objectives:
• Understanding the concepts and importance of exercise adherence and its promotion in clinical practice.
• Relate to and reflect on the challenges of exercise adherence from provider, patient, and system perspectives.
• Appreciate the peculiar challenges of physical activity for peP2ople with multimorbidity, physical limitations, and for the elderly.
• Learn about evidence-based principles and practical strategies of encouraging exercise adherence.
• Apply the learned principles to hypothetical patient cases with painful Diabetic Neuropathy, developing exercise adherence strategies tailored to the specific challenges presented in these cases.</td><td>Practical Lesson 3:

<u>The Neurological Assessment of People with Suspected Peripheral Neuropathy</u>

Speaker: Dr Matthew Evans & Dr David Hohenschurz-Schmidt
Duration: 1 hour
Delivery mode: Hybrid: Recorded & Face-to-face
Learning objectives:
• Translate previously gained knowledge into a case history for people with suspected pDPN.
• Understand the nature, rationale, and validity of common bedside screening tests.
• Apply screening tests expertly.
Content:
• Go through questionnaire completion in roleplay.
• Practical bedside neurological testing</td></tr>
</table>

• Engage in and reflect on the practical application of exercise adherence principles in participants' next clinical session.<br>Content:<br>TBC	
Theory Lesson 5:<br><br><u>Manual Therapy in pDPN: Rationale and Evidence</u><br>Speaker: Dr David Hohenschurz-Schmidt<br>Duration: 0.5 hour<br>Delivery mode: Recorded<br>Learning objectives:<br>• Comprehend the Rationale Behind Manual Therapy in Painful Diabetic Neuropathy (pDPN):<br>• Explore neurological and musculoskeletal changes in people living with pDPN that may be amenable to manual therapy.<br>• Explore the potential mechanisms and target outcomes of manual therapy in pDPN.<br>• Appreciate the views of manual therapy providers and of patients on the potential role of manual therapy in the management of people with pDPN.<br>• Evaluate the Existing Evidence for Manual Therapy in pDPN.<br>• Explore the Patient-Centred Application of Manual Therapy:<br>• Learn about the core manual therapy techniques proposed as part of the NeuOst intervention.<br>• Understand how manual therapy can be tailored to individual patients.<br>• Appreciate how Manual Therapy Integrates with Other Components of the multimodal NeuOst Intervention.<br>• Develop an Awareness of Potential Risks and Contraindications Associated with Manual Therapy in Individuals with pDPN.<br>Content:<br>• Changes in pDPN relevant for MT<br>• Rationale for MT techniques in pDPN<br>• Integration within NeuOst intervention<br>• Research evidence and gaps<br>• Provider and patient views<br>• Techniques<br>• Safety considerations	Practical Lesson 4:<br><br><u>Expert Q&A Session: The Experience of Living with DPN and Neuropathic Pain</u><br><br>Speaker: Mrs Elizabeth Piggott<br>Duration: 1 hour<br>Delivery mode: face-to-face<br>Learning objectives:<br>• Appreciate and reflect on the personal experience and impact of living with DPN and pDPN.<br>• Engage with a person living with pDPN.<br>• Reflect on the implications for clinical management.<br>Content:<br>• Informal presentation of patient partner<br>• Open conversation / Q&A in plenum<br>○ How did your condition develop?<br>○ When and how does it affect you most?<br>○ What do you do to manage it?<br>○ What was / is your experience with your medical management?<br>○ What have you learnt from talking to other people with this condition?
Theory Lesson 6 (**optional**):<br><br><u>Placebo Effects in Osteopathy</u><br><br>Speaker: Dr David Hohenschurz-Schmidt<br>Duration: 1 hour<br>Delivery mode: Recorded<br>Learning outcomes:	Practical Lesson 5<br><br><u>Patient-Centred Communication when Working with People with Persistent Pain</u><br><br>Speaker: Steven Vogel<br>Duration: 2 hours<br>Delivery mode: Face-to-face<br>Learning objectives:

- Appreciate the historical development of the concepts: placebo, nocebo, placebo effects, and placebo response and understand their current use and challenges.<br>- Understand the neurophysiological and psychological mechanisms underpinning placebo effects, especially regarding pain.<br>- Relate the context-rich nature of osteopathic clinical practice to the potential for placebo and nocebo effects.<br>- Reflect on the implications of the current science of the placebo effect for our mechanistic understanding and research of osteopathy and for clinical practice.<br>Content:<br>• Placebo and nocebo effects: description, science, and mechanisms<br>• Implications for osteopathic practice, education, and research	• To explore the practicalities and core communication skills when working in partnership with patients with persistent pain.<br>• To explore opportunities to enhance person centred care and communication.<br>Content:<br>• Characteristics of person-centred care<br>• Role plays with observer feedback and peer discussions.<br>• Communication skills: Active listening, initiating the consultation, verbal and nonverbal communication, self-awareness.
Theory Lesson 7 (**only required from NeuOst trial providers**):<br><br><u>Nonpharmacological Intervention Research and the NeuOst Feasibility Trial</u><br><br>*Talk 1: The Fundamentals of Nonpharmacological Intervention Research and of Placebo Controlled Trials*<br><br>Speaker: DHS<br>Duration: 0.5 hours<br>Delivery mode: Recorded<br>Learning objectives:<br>• Appreciate the challenges of nonpharmacological / complex intervention clinical research, especially in so-called efficacy trials.<br>• Appreciate the potential for placebo effects in physical and psychological interventions and their implications for trial design.<br>• Understand the concepts of blinding and control interventions in efficacy trials.<br>• Know about current best-practice frameworks for control intervention design.<br>Content:<br>• 30-mins recording of a webinar held for the British Pain Society<br>• Includes an optional 30-mins talk by Prof David Beard on placebo-controlled surgery trials and a panel discussion.<br><br>*Talk 2: The NeuOst Trial Protocol and Considerations for Trial Providers*<br><br>Speaker: DHS<br>Duration: 0.5 hours<br>Delivery mode: Synchronous e-learning (live delivery after in-practice training day for selected trial providers)<br>Learning objectives:	Practical Lesson 6<br><br><u>Manual Therapy and Exercise Components of NeuOst</u><br><br>Speaker: DHS<br>Duration: 1 hour<br>Delivery mode: Face-to-face<br>Learning objectives:<br>• Learn the content and practical delivery of NeuOst manual therapy components.<br>• Learn the content and practical delivery of NeuOst physical activity components.<br>• Understand the flexibility in the programme contents and how to tailor recommendations / content.<br>• Practice how to integrate communication skills and psychologically informed methods into the delivery of manual and exercise components<br>Content:<br>• Summary of intervention theory and elaboration on intervention component interactions in theory and practice<br>• Detailed introduction to the manual therapy and exercise components of NeuOst.<br>• Practical on manual therapy and exercise administration amongst peers.<br>• Reflection on challenges for NeuOst administration in clinical practice.

• Understand the basic design elements NeuOst feasibility trial.<br>• Appreciate the tasks and role of clinical providers in an RCT in general and in NeuOst in particular.<br>• Reflect on and discuss concerns and challenges as trial providers.<br>• Gain access to materials for providers, such as detailed session protocol and ACT handbook.<br>• Commence practical feasibility trial participation planning, such as populating weekly diaries.<br>Content:<br>• Key design elements of the NeuOst feasibility trial<br>• Role and responsibilities of clinical trial providers in sham-controlled trials in general and in the NeuOst feasibility trial in particular.<br>• Experiences of trial providers from relevant related RCTs.<br>• Relevant ethical considerations.<br>• Reflection and discussion amongst participants.<br>• Training tasks.	

Aditional training for designated trial providers

For designated trial providers, the NeuOst training course included two obligatory lectures on 1) the Fundamentals of Nonpharmacological Intervention Research and of Placebo Controlled Trials, and 2) the NeuOst Trial Protocol and Considerations for Trial Providers, delivered by the chief investigator. This covered the following learning objectives and content. Course completion was ensured by the chief investigator and understanding was tested by means of a quiz.

Talk 1: The Fundamentals of Nonpharmacological Intervention Research and of Placebo Controlled Trials (Duration: 0.5 hours, Delivery mode: Recorded)

Learning objectives:

- Appreciate the challenges of nonpharmacological / complex intervention clinical research, especially in so-called efficacy trials.
- Appreciate the potential for placebo effects in physical and psychological interventions and their implications for trial design.
- Understand the concepts of blinding and control interventions in efficacy trials.
- Know about current best-practice frameworks for control intervention design.

Content:

- 30-mins recording of a webinar held for the British Pain Society (full recording: https://www.youtube.com/watch?v=WI4jaqf5_PE)
- Webinar includes an optional 30-mins talk by Prof David Beard on placebo-controlled surgery trials and a panel discussion.

Talk 2: The NeuOst Trial Protocol and Considerations for Trial Providers (Duration: 0.5 hours, Delivery mode: Synchronous e-learning - live delivery after in-practice training day for selected trial providers)

Learning objectives:

- Understand the basic design elements NeuOst feasibility trial.
- Appreciate the tasks and role of clinical providers in an RCT in general and in NeuOst in particular.
- Reflect on and discuss concerns and challenges as trial providers.
- Gain access to materials for providers, such as detailed session protocol and ACT handbook.
- Commence practical feasibility trial participation planning, such as populating weekly diaries.

Content:

- Key design elements of the NeuOst feasibility trial
- Role and responsibilities of clinical trial providers in sham-controlled trials in general and in the NeuOst feasibility trial in particular.
- Experiences of trial providers from relevant related RCTs.

- Relevant ethical considerations.
- Reflection and discussion amongst participants.
- Training tasks.

Finally, designated trial providers attended a practical 2-hour session with two patient partners, where the complete intervention and control intervention protocols were practised in small groups. Afterwards, providers reflected on the experience, facilitated by the chief investigator.

References, SDC part 3

Abate, M., Schiavone, C., Salini, V., Andia, I., 2013. Occurrence of tendon pathologies in metabolic disorders. Rheumatology 52, 599–608. https://doi.org/10.1093/rheumatology/kes395

About osteopathy | Institute of Osteopathy [WWW Document], n.d. URL https://www.iosteopathy.org/about-osteopathy/ (accessed 12.3.21).

Ahmad, I., Verma, S., Noohu, M.M., Shareef, Mohd.Y., Hussain, M.E., 2020. Sensorimotor and gait training improves proprioception, nerve function, and muscular activation in patients with diabetic peripheral neuropathy: a randomized control trial. J Musculoskelet Neuronal Interact 20, 234–248.

Alam, U., Riley, D.R., Jugdey, R.S., Azmi, S., Rajbhandari, S., D'Août, K., Malik, R.A., 2017. Diabetic Neuropathy and Gait: A Review. Diabetes Ther 8, 1253–1264. https://doi.org/10.1007/s13300-017-0295-y

Almurdhi, M.M., Reeves, N.D., Bowling, F.L., Boulton, A.J.M., Jeziorska, M., Malik, R.A., 2016. Reduced Lower-Limb Muscle Strength and Volume in Patients With Type 2 Diabetes in Relation to Neuropathy, Intramuscular Fat, and Vitamin D Levels. Diabetes Care 39, 441–447. https://doi.org/10.2337/dc15-0995

Amato Nesbit, S., Sharma, R., Waldfogel, J.M., Zhang, A., Bennett, W.L., Yeh, H.-C., Chelladurai, Y., Feldman, D., Robinson, K.A., Dy, S.M., 2019. Non-pharmacologic treatments for symptoms of diabetic peripheral neuropathy: a systematic review. Curr Med Res Opin 35, 15–25. https://doi.org/10.1080/03007995.2018.1497958

Andersen, H., 2012. Motor dysfunction in diabetes. Diabetes/Metabolism Research and Reviews 28, 89–92. https://doi.org/10.1002/dmrr.2257

Andersen Hammond, E., Pitz, M., Steinfeld, K., Lambert, P., Shay, B., 2020. An Exploratory Randomized Trial of Physical Therapy for the Treatment of Chemotherapy-Induced Peripheral Neuropathy. Neurorehabil Neural Repair 34, 235–246. https://doi.org/10.1177/1545968319899918

Ashar, Y.K., Gordon, A., Schubiner, H., Uipi, C., Knight, K., Anderson, Z., Carlisle, J., Polisky, L., Geuter, S., Flood, T.F., Kragel, P.A., Dimidjian, S., Lumley, M.A., Wager, T.D., 2021. Effect of Pain Reprocessing Therapy vs Placebo and Usual Care for Patients With Chronic Back Pain: A Randomized Clinical Trial. JAMA Psychiatry. https://doi.org/10.1001/jamapsychiatry.2021.2669

Balk, E.M., Earley, A., Raman, G., Avendano, E.A., Pittas, A.G., Remington, P.L., 2015. Combined Diet and Physical Activity Promotion Programs to Prevent Type 2 Diabetes Among Persons at Increased Risk: A Systematic Review for the Community Preventive Services Task Force. Ann Intern Med 163, 437–451. https://doi.org/10.7326/M15-0452

Ball, E.F., Nur Shafina Muhammad Sharizan, E., Franklin, G., Rogozińska, E., 2017. Does mindfulness meditation improve chronic pain? A systematic review. Current Opinion in Obstetrics and Gynecology 29, 359–366. https://doi.org/10.1097/GCO.0000000000000417

Baskerville, R., Ricci-Cabello, I., Roberts, N., Farmer, A., 2017. Impact of accelerometer and pedometer use on physical activity and glycaemic control in people with Type 2 diabetes: a systematic review and meta-analysis. Diabet Med 34, 612–620. https://doi.org/10.1111/dme.13331

Bialosky, J.E., Beneciuk, J.M., Bishop, M.D., Coronado, R.A., Penza, C.W., Simon, C.B., George, S.Z., 2017. Unraveling the Mechanisms of Manual Therapy: Modeling an Approach. J Orthop Sports Phys Ther 48, 8–18. https://doi.org/10.2519/jospt.2018.7476

Bishop, M.D., Torres-Cueco, R., Gay, C.W., Lluch-Girbés, E., Beneciuk, J.M., Bialosky, J.E., 2015. What effect can manual therapy have on a patient's pain experience? Pain Management 5, 455–464. https://doi.org/10.2217/pmt.15.39

Bono Mira, M., 2008. EFFECTS OF THE MANIPULATION OF D6 IN THE GLUCEMIA OF DIABETIC PATIENTS [WWW Document].

Çakici, N., Fakkel, T.M., van Neck, J.W., Verhagen, A.P., Coert, J.H., 2016. Systematic review of treatments for diabetic peripheral neuropathy. Diabet Med 33, 1466–1476. https://doi.org/10.1111/dme.13083

Carnes, D., Mars, T., Plunkett, A., Nanke, L., Abbey, H., 2017. A mixed methods evaluation of a third wave cognitive behavioural therapy and osteopathic treatment programme for chronic pain in primary care (OsteoMAP). International Journal of Osteopathic Medicine 24, 12–17. https://doi.org/10.1016/j.ijosm.2017.03.005

Carpenter, M., 2016. can an osteopathic rib-raising technique influence blood glucose levels in type 2 diabetic patients? An ABAB single case research design. [WWW Document].

Castelnuovo, G., Pietrabissa, G., Manzoni, G.M., Cattivelli, R., Rossi, A., Novelli, M., Varallo, G., Molinari, E., 2017. Cognitive behavioral therapy to aid weight loss in obese patients: Current perspectives. Psychology Research and Behavior Management 10. https://doi.org/10.2147/PRBM.S113278

Chan, C.L., Taljaard, M., Lancaster, G.A., Brehaut, J.C., Eldridge, S.M., 2021. Pilot and feasibility studies for pragmatic trials have unique considerations and areas of uncertainty. Journal of Clinical Epidemiology 138, 102–114. https://doi.org/10.1016/j.jclinepi.2021.06.029

Chao, C.-C., Tseng, M.-T., Hsieh, P.-C., Lin, C.-H. (Janice), Huang, S.-L., Hsieh, S.-T., Chiang, M.-C., 2021. Brain Mechanisms of Pain and Dysautonomia in Diabetic Neuropathy: Connectivity Changes in Thalamus and Hypothalamus. The Journal of Clinical Endocrinology & Metabolism. https://doi.org/10.1210/clinem/dgab754

Chatchawan, U., Eungpinichpong, W., Plandee, P., Yamauchi, J., 2015. Effects of Thai Foot Massage on Balance Performance in Diabetic Patients with Peripheral Neuropathy: A Randomized Parallel-Controlled Trial. Med Sci Monit Basic Res 21, 68–75. https://doi.org/10.12659/MSMBR.894163

Colberg, S.R., Sigal, R.J., Yardley, J.E., Riddell, M.C., Dunstan, D.W., Dempsey, P.C., Horton, E.S., Castorino, K., Tate, D.F., 2016. Physical Activity/Exercise and Diabetes: A Position Statement of the American Diabetes Association. Diabetes Care 39, 2065–2079. https://doi.org/10.2337/dc16-1728

Craig, P., Campbell, M., 2015. Evaluability Assessment: a systematic approach to deciding whether and how to evaluate programmes and policies: a What Works Scotland Working paper. https://doi.org/10.13140/RG.2.1.2007.4725

Davies, B., Cramp, F., Gauntlett-Gilbert, J., Wynick, D., McCabe, C.S., 2015. The role of physical activity and psychological coping strategies in the management of painful diabetic neuropathy – A systematic review of the literature. Physiotherapy 101, 319–326. https://doi.org/10.1016/j.physio.2015.04.003

Davies, M., Brophy, S., Williams, R., Taylor, A., 2006. The Prevalence, Severity, and Impact of Painful Diabetic Peripheral Neuropathy in Type 2 Diabetes. Diabetes Care 29, 1518–1522. https://doi.org/10.2337/dc05-2228

Díaz Cerrato, I., 2008. MODIFICACIONES EN LA PIO Y EN LA TA EN DIABÉTICOS TIPO I TRAS LA MANIPULACIÓN GLOBAL OAA SEGÚN FRYETTE.(ECA) [WWW Document].

Dixit, S., Asiri, F., 2014. Pharmacological and nonpharmacological therapies in the management of diabetic peripheral neuropathy in type 2 diabetes: A comprehensive review. Journal of Cardiovascular Disease Research 5.

Eklund, A., Jensen, I., Lohela-Karlsson, M., Hagberg, J., Leboeuf-Yde, C., Kongsted, A., Bodin, L., Axén, I., 2018. The Nordic Maintenance Care program: Effectiveness of chiropractic maintenance care versus symptom-guided treatment for recurrent and persistent low back pain—A pragmatic randomized controlled trial. PLOS ONE 13, e0203029. https://doi.org/10.1371/journal.pone.0203029

Eldridge, S.M., Chan, C.L., Campbell, M.J., Bond, C.M., Hopewell, S., Thabane, L., Lancaster, G.A., 2016a. CONSORT 2010 statement: extension to randomised pilot and feasibility trials. BMJ 355, i5239. https://doi.org/10.1136/bmj.i5239

Eldridge, S.M., Lancaster, G.A., Campbell, M.J., Thabane, L., Hopewell, S., Coleman, C.L., Bond, C.M., 2016b. Defining Feasibility and Pilot Studies in Preparation for Randomised Controlled Trials: Development of a Conceptual Framework. PLoS One 11. https://doi.org/10.1371/journal.pone.0150205

Espelt, A., Arriola, L., Borrell, C., Larranaga, I., Sandin, M., Escolar-Pujolar, A., 2011. Socioeconomic Position and Type 2 Diabetes Mellitus in Europe 1999- 2009: a Panorama of Inequalities. Current Diabetes Reviews 7, 148–158. https://doi.org/10.2174/157339911795843131

Estabrooks, P.A., Glasgow, R.E., Dzewaltowski, D.A., 2003. Physical activity promotion through primary care. JAMA 289, 2913–2916. https://doi.org/10.1001/jama.289.22.2913

Evans, M.C., Wade, C., Hohenschurz-Schmidt, D., Lally, P., Ugwudike, A., Shah, K., Bangerter, N., Sharp, D.J., Rice, A.S.C., 2021. Magnetic Resonance Imaging as a Biomarker in Diabetic and HIV-Associated Peripheral Neuropathy: A Systematic Review-Based Narrative. Frontiers in Neuroscience 15, 1169. https://doi.org/10.3389/fnins.2021.727311

Evans, R.E., Moore, G., Movsisyan, A., Rehfuess, E., 2021. How can we adapt complex population health interventions for new contexts? Progressing debates and research priorities. J Epidemiol Community Health 75, 40–45. https://doi.org/10.1136/jech-2020-214468

Fawkes, C., Carnes, D., 2021. Patient reported outcomes in a large cohort of patients receiving osteopathic care in the United Kingdom. PLOS ONE 16, e0249719. https://doi.org/10.1371/journal.pone.0249719

Fawkes, C., Leach, J., Matias, S., Moore, A., 2009. The standardised data collection project. Standardised data collection within osteopathic practice in the UK: development and first use of a tool to profile osteopathic care in 2010.

Ferini-Strambi, L., 2017. Neuropathic Pain and Sleep: A Review. Pain Ther 6, 19–23. https://doi.org/10.1007/s40122-017-0089-y

Finnerup, N.B., Attal, N., Haroutounian, S., McNicol, E., Baron, R., Dworkin, R.H., Gilron, I., Haanpää, M., Hansson, P., Jensen, T.S., Kamerman, P.R., Lund, K., Moore, A., Raja, S.N., Rice, A.S.C., Rowbotham, M., Sena, E., Siddall, P., Smith, B.H., Wallace, M., 2015. Pharmacotherapy for neuropathic pain in adults: a systematic review and

meta-analysis. The Lancet Neurology 14, 162–173. https://doi.org/10.1016/S1474-4422(14)70251-0

Fjeldsoe, B., Neuhaus, M., Winkler, E., Eakin, E., 2011. Systematic review of maintenance of behavior change following physical activity and dietary interventions. Health Psychology 30, 99–109. https://doi.org/10.1037/a0021974

Franssen, W.M.A., Franssen, G.H.L.M., Spaas, J., Solmi, F., Eijnde, B.O., 2020. Can consumer wearable activity tracker-based interventions improve physical activity and cardiometabolic health in patients with chronic diseases? A systematic review and meta-analysis of randomised controlled trials. Int J Behav Nutr Phys Act 17, 57. https://doi.org/10.1186/s12966-020-00955-2

Freedland, K.E., King, A.C., Ambrosius, W.T., Mayo-Wilson, E., Mohr, D.C., Czajkowski, S.M., Thabane, L., Collins, L.M., Rebok, G.W., Treweek, S.P., Cook, T.D., Edinger, J.D., Stoney, C.M., Campo, R.A., Young-Hyman, D., Riley, W.T., 2019. The selection of comparators for randomized controlled trials of health-related behavioral interventions: recommendations of an NIH expert panel. Journal of Clinical Epidemiology 110, 74–81. https://doi.org/10.1016/j.jclinepi.2019.02.011

Fu, Q., Yang, H., Zhang, L., Liu, Yang, Li, X., Dai, M., Yang, Y., Yang, S., Xie, Y., Liu, Ying, Fu, L., Liu, Z., Zhang, Q., 2020. Traditional Chinese medicine foot bath combined with acupoint massage for the treatment of diabetic peripheral neuropathy: A systematic review and meta-analysis of 31 RCTs. Diabetes/Metabolism Research and Reviews 36, e3218. https://doi.org/10.1002/dmrr.3218

General Osteopathic Council, 2018. Standards of practice [WWW Document]. URL https://www.osteopathy.org.uk/standards/osteopathic-practice/ (accessed 4.20.20).

Gilron, I., Baron, R., Jensen, T., 2015. Neuropathic Pain: Principles of Diagnosis and Treatment. Mayo Clinic Proceedings 90, 532–545. https://doi.org/10.1016/j.mayocp.2015.01.018

Godfrey, E., Holmes, M.G., Wileman, V., McCracken, L., Norton, S., Moss-Morris, R., Pallet, J., Sanders, D., Barcellona, M., Critchley, D., 2016. Physiotherapy informed by Acceptance and Commitment Therapy (PACT): protocol for a randomised controlled trial of PACT versus usual physiotherapy care for adults with chronic low back pain. BMJ Open 6, e011548. https://doi.org/10.1136/bmjopen-2016-011548

Godfrey, E., Wileman, V., Galea Holmes, M., McCracken, L.M., Norton, S., Moss-Morris, R., Noonan, S., Barcellona, M., Critchley, D., 2020. Physical Therapy Informed by Acceptance and Commitment Therapy (PACT) Versus Usual Care Physical Therapy for Adults With Chronic Low Back Pain: A Randomized Controlled Trial. The Journal of Pain 21, 71–81. https://doi.org/10.1016/j.jpain.2019.05.012

Grewal, G.S., Schwenk, M., Lee-Eng, J., Parvaneh, S., Bharara, M., Menzies, R.A., Talal, T.K., Armstrong, D.G., Najafi, B., 2015. Sensor-Based Interactive Balance Training with Visual Joint Movement Feedback for Improving Postural Stability in Diabetics with Peripheral Neuropathy: A Randomized Controlled Trial. GER 61, 567–574. https://doi.org/10.1159/000371846

Hall, K.T., Loscalzo, J., 2019. Drug-Placebo Additivity in Randomized Clinical Trials. Clinical Pharmacology & Therapeutics 106, 1191–1197. https://doi.org/10.1002/cpt.1626

Hamid, N., 2011. Effects of stress management training on glycemic control in women with type 2 diabetes. Iranian Journal of Endocrinology and Metabolism 13, 346–353.

Hart, A.J.P., Beckley, K., 2015. 5. Acceptance and Commitment Therapy, in: Formulation in ActionApplying Psychological Theory to Clinical Practice. De Gruyter, Berlin, Boston. https://doi.org/10.1515/9783110471014-007

Harvey, E., Burton, A.K., Moffett, J.K., Breen, A., 2003. Spinal manipulation for low-back pain: a treatment package agreed by the UK chiropractic, osteopathy and physiotherapy professional associations. Manual Therapy 8, 46–51. https://doi.org/10.1054/math.2002.0472

He, X., Li, J., Wang, B., Yao, Q., Li, L., Song, R., Shi, X., Zhang, J., 2017. Diabetes self-management education reduces risk of all-cause mortality in type 2 diabetes patients: a systematic review and meta-analysis. Endocrine 55, 712–731. https://doi.org/10.1007/s12020-016-1168-2

Hernández-Secorún, M., Vidal-Peracho, C., Márquez-Gonzalvo, S., Corral-de-Toro, J., Müller-Thyssen-Uriarte, J., Rodríguez-Sanz, J., Lucha-López, M.O., Tricás-Moreno, J.M., Hidalgo-García, C., 2021. Exercise and Manual Therapy for Diabetic Peripheral Neuropathy: A Systematic Review. Applied Sciences 11, 5665. https://doi.org/10.3390/app11125665

Hewston, P., Deshpande, N., 2018. Fear of Falling and Balance Confidence in Older Adults With Type 2 Diabetes Mellitus: A Scoping Review. Canadian Journal of Diabetes 42, 664–670. https://doi.org/10.1016/j.jcjd.2018.02.009

Hill-Briggs, F., Adler, N.E., Berkowitz, S.A., Chin, M.H., Gary-Webb, T.L., Navas-Acien, A., Thornton, P.L., Haire-Joshu, D., 2021. Social Determinants of Health and Diabetes: A Scientific Review. Diabetes Care 44, 258–279. https://doi.org/10.2337/dci20-0053

Hodkinson, A., Kontopantelis, E., Adeniji, C., van Marwijk, H., McMillian, B., Bower, P., Panagioti, M., 2021. Interventions Using Wearable Physical Activity Trackers Among Adults With Cardiometabolic Conditions: A Systematic Review and Meta-analysis. JAMA Netw Open 4, e2116382. https://doi.org/10.1001/jamanetworkopen.2021.16382

International Diabetes Federation, 2019. IDF Diabetes Atlas 9th edition [WWW Document]. URL https://diabetesatlas.org/en/ (accessed 9.8.21).

Izgu, N., Gok Metin, Z., Karadas, C., Ozdemir, L., Metinarikan, N., Corapcıoglu, D., 2020. Progressive Muscle Relaxation and Mindfulness Meditation on Neuropathic Pain, Fatigue, and Quality of Life in Patients With Type 2 Diabetes: A Randomized Clinical Trial. Journal of Nursing Scholarship 52, 476–487. https://doi.org/10.1111/jnu.12580

Jackson, W., Zale, E.L., Berman, S.J., Malacarne, A., Lapidow, A., Schatman, M.E., Kulich, R., Vranceanu, A.-M., 2019. Physical functioning and mindfulness skills training in chronic pain: a systematic review. J Pain Res 12, 179–189. https://doi.org/10.2147/JPR.S172733

Javed, S., Petropoulos, I.N., Alam, U., Malik, R.A., 2015. Treatment of painful diabetic neuropathy. Therapeutic Advances in Chronic Disease 6, 15–28. https://doi.org/10.1177/2040622314552071

Karran, E.L., Grant, A.R., Moseley, G.L., 2020. Low back pain and the social determinants of health: a systematic review and narrative synthesis. PAIN 161, 2476–2493. https://doi.org/10.1097/j.pain.0000000000001944

Kec, D., Rajdova, A., Raputova, J., Adamova, B., Srotova, I., Nekvapilova, E.K., Michalcakova, R.N., Horakova, M., Belobradkova, J., Olsovsky, J., Weber, P., Hajas, G., Kaiserova, M., Mazanec, R., Potockova, V., Ehler, E., Forgac, M., Birklein, F., Üçeyler, N., Sommer, C., Bednarik, J., Vlckova, E., 2021. Risk factors for depression and anxiety in painful and painless diabetic polyneuropathy: A multicentre observational cross-sectional study. European Journal of Pain n/a. https://doi.org/10.1002/ejp.1865

Keefe, F.J., Main, C.J., George, S.Z., 2018. Advancing Psychologically Informed Practice for Patients With Persistent Musculoskeletal Pain: Promise, Pitfalls, and Solutions. Phys Ther 98, 398–407. https://doi.org/10.1093/ptj/pzy024

Kerr, F., Wiechula, R., Feo, R., Schultz, T., Kitson, A., 2019. Neurophysiology of human touch and eye gaze in therapeutic relationships and healing: a scoping review. JBI Database System Rev Implement Rep 17, 209–247. https://doi.org/10.11124/JBISRIR-2017-003549

Khan, K.S., Andersen, H., 2022. The Impact of Diabetic Neuropathy on Activities of Daily Living, Postural Balance and Risk of Falls - A Systematic Review. J Diabetes Sci Technol 16, 289–294. https://doi.org/10.1177/1932296821997921

Kiegerl, G., 2007. Does Osteopathy Influence Diabetes Mellitus Type II? [WWW Document].

King, H.H., 2016. Manual Therapy Shown To Improve Diabetic Foot Ulcer Healing. Journal of Osteopathic Medicine 116, 685–686. https://doi.org/10.7556/jaoa.2016.135

Kioskli, K., Scott, W., Winkley, K., Godfrey, E., McCracken, L., 2020. Online Acceptance and Commitment Therapy for people with painful diabetic neuropathy in the United Kingdom: A single-arm feasibility trial. Pain Med 1–35.

Kioskli, K., Scott, W., Winkley, K., Kylakos, S., McCracken, L.M., 2019a. Psychosocial Factors in Painful Diabetic Neuropathy: A Systematic Review of Treatment Trials and Survey Studies. Pain Medicine 20, 1756–1773. https://doi.org/10.1093/pm/pnz071

Kioskli, K., Winkley, K., McCracken, L.M., 2019b. Might psychological flexibility processes and Acceptance and Commitment Therapy (ACT) apply in adults with painful diabetic neuropathy? A cross-sectional survey. Journal of Contextual Behavioral Science 13, 66–73. https://doi.org/10.1016/j.jcbs.2019.07.002

Kuhmann, O., 2008. The impact of osteopathic treatment on intraocular hypertension – an experimental study [WWW Document].

Lau, M.A., McMain, S.F., 2005. Integrating Mindfulness Meditation with Cognitive and Behavioural Therapies: The Challenge of Combining Acceptance- and Change-Based Strategies. Can J Psychiatry 50, 863–869. https://doi.org/10.1177/070674370505001310

Liampas, A., Rekatsina, M., Vadalouca, A., Paladini, A., Varrassi, G., Zis, P., 2020. Non-Pharmacological Management of Painful Peripheral Neuropathies: A Systematic Review. Adv Ther 37, 4096–4106. https://doi.org/10.1007/s12325-020-01462-3

Licciardone, J.C., Kearns, C.M., Hodge, L.M., Minotti, D.E., 2013. Osteopathic Manual Treatment in Patients With Diabetes Mellitus and Comorbid Chronic Low Back Pain: Subgroup Results From the OSTEOPATHIC Trial. Journal of Osteopathic Medicine 113, 468–478. https://doi.org/10.7556/jaoa.2013.113.6.468

Lin, T., Huang, F., Zhao, S., Qiu, M., Wen, J., Liu, M., 2021. Acupuncture for diabetic peripheral neuropathy: An overview of systematic reviews. Complementary Therapies in Clinical Practice 43, 101375. https://doi.org/10.1016/j.ctcp.2021.101375

Maggard-Gibbons, M., Maglione, M., Livhits, M., Ewing, B., Maher, A.R., Hu, J., Li, Z., Shekelle, P.G., 2013. Bariatric Surgery for Weight Loss and Glycemic Control in Nonmorbidly Obese Adults With Diabetes: A Systematic Review. JAMA 309, 2250–2261. https://doi.org/10.1001/jama.2013.4851

Marmot, M., 2020. Health equity in England: the Marmot review 10 years on. BMJ 368. https://doi.org/10.1136/bmj.m693

McClintock, A.S., McCarrick, S.M., Garland, E.L., Zeidan, F., Zgierska, A.E., 2019. Brief Mindfulness-Based Interventions for Acute and Chronic Pain: A Systematic Review.

The Journal of Alternative and Complementary Medicine 25, 265–278. https://doi.org/10.1089/acm.2018.0351

McCracken, L.M., Morley, S., 2014. The Psychological Flexibility Model: A Basis for Integration and Progress in Psychological Approaches to Chronic Pain Management. The Journal of Pain 15, 221–234. https://doi.org/10.1016/j.jpain.2013.10.014

McCracken, L.M., Yu, L., Vowles, K.E., 2022. New generation psychological treatments in chronic pain. BMJ 376, e057212. https://doi.org/10.1136/bmj-2021-057212

McGuire, H., Longson, D., Adler, A., Farmer, A., Lewin, I., 2016. Management of type 2 diabetes in adults: summary of updated NICE guidance. BMJ 353, i1575. https://doi.org/10.1136/bmj.i1575

McGuirk, J., 2012. The place of touch in Counselling and Psychotherapy and the potential for healing within the therapeutic relationship. Inside Out 68.

Meisinger, C., Bongaerts, B.W.C., Heier, M., Amann, U., Kowall, B., Herder, C., Rückert-Eheberg, I.-M., Rathmann, W., Ziegler, D., 2018. Neuropathic pain is not adequately treated in the older general population: Results from the KORA F4 survey. Pharmacoepidemiol Drug Saf 27, 806–814. https://doi.org/10.1002/pds.4559

Moisset, X., Bouhassira, D., Avez Couturier, J., Alchaar, H., Conradi, S., Delmotte, M.H., Lanteri-Minet, M., Lefaucheur, J.P., Mick, G., Piano, V., Pickering, G., Piquet, E., Regis, C., Salvat, E., Attal, N., 2020. Pharmacological and non-pharmacological treatments for neuropathic pain: Systematic review and French recommendations. Rev Neurol (Paris) 176, 325–352. https://doi.org/10.1016/j.neurol.2020.01.361

Molsted, S., Tribler, J., Snorgaard, O., 2012. Musculoskeletal pain in patients with type 2 diabetes. Diabetes Research and Clinical Practice 96, 135–140. https://doi.org/10.1016/j.diabres.2011.12.022

Naranjo, C., Del Reguero, L., Moratalla, G., Hercberg, M., Valenzuela, M., Failde, I., 2019. Anxiety, depression and sleep disorders in patients with diabetic neuropathic pain: a systematic review. Expert Review of Neurotherapeutics 19, 1201–1209. https://doi.org/10.1080/14737175.2019.1653760

Nathan, H.J., Poulin, P., Wozny, D., Taljaard, M., Smyth, C., Gilron, I., Sorisky, A., Lochnan, H., Shergill, Y., 2017. Randomized Trial of the Effect of Mindfulness-Based Stress Reduction on Pain-Related Disability, Pain Intensity, Health-Related Quality of Life, and A1C in Patients With Painful Diabetic Peripheral Neuropathy. Clinical Diabetes 35, 294–304. https://doi.org/10.2337/cd17-0077

NICE, 2015. Overview | Type 2 diabetes in adults: management | Guidance | NICE [WWW Document]. URL https://www.nice.org.uk/guidance/ng28 (accessed 9.8.21).

Onifer, S.M., Sozio, R.S., DiCarlo, D.M., Li, Q., Donahue, R.R., Taylor, B.K., Long, C.R., 2018. Spinal Manipulative Therapy Reduces Peripheral Neuropathic Pain in the Rat. Neuroreport 29, 191–196. https://doi.org/10.1097/WNR.0000000000000949

Pandey, A., Tripathi, P., Pandey, R., Srivatava, R., Goswami, S., 2011. Alternative therapies useful in the management of diabetes: A systematic review. J Pharm Bioallied Sci 3, 504–512. https://doi.org/10.4103/0975-7406.90103

Parasoglou, P., Rao, S., Slade, J.M., 2017. Declining Skeletal Muscle Function in Diabetic Peripheral Neuropathy. Clinical Therapeutics 39, 1085–1103. https://doi.org/10.1016/j.clinthera.2017.05.001

Pêgas de Oliveira, A., 2008. VERIFICATION OF THE ALTERATIONS OF GLUCEMIA AND INSULINEMIA IN PATIENTS WITH DIABETES TYPE II SUBMISSIVE TO A PROTOCOL OF OSTEOPATHIC TREATMENT [WWW Document].

Plunkett, A., Fawkes, C., Carnes, D., 2021. UK osteopathic practice in 2019: a retrospective analysis of practice data. https://doi.org/10.1101/2021.01.28.21250601

Racaru, S., Sturt, J., Celik, A., 2021. The Effects of Psychological Interventions on Diabetic Peripheral Neuropathy: A Systematic Review and Meta-Analysis. Pain Management Nursing 22, 302–311. https://doi.org/10.1016/j.pmn.2020.11.001

Raveendran, A.V., Chacko, E.C., Pappachan, J.M., 2018. Non-pharmacological Treatment Options in the Management of Diabetes Mellitus. Eur Endocrinol 14, 31–39. https://doi.org/10.17925/EE.2018.14.2.31

Rossen, J., Larsson, K., Hagströmer, M., Yngve, A., Brismar, K., Ainsworth, B., Åberg, L., Johansson, U.-B., 2021. Effects of a three-armed randomised controlled trial using self-monitoring of daily steps with and without counselling in prediabetes and type 2 diabetes-the Sophia Step Study. Int J Behav Nutr Phys Act 18, 121. https://doi.org/10.1186/s12966-021-01193-w

Safren, S.A., Gonzalez, J.S., Wexler, D.J., Psaros, C., Delahanty, L.M., Blashill, A.J., Margolina, A.I., Cagliero, E., 2014. A Randomized Controlled Trial of Cognitive Behavioral Therapy for Adherence and Depression (CBT-AD) in Patients With Uncontrolled Type 2 Diabetes. Diabetes Care 37, 625–633. https://doi.org/10.2337/dc13-0816

Said, G., 2007. Diabetic neuropathy—a review. Nat Rev Neurol 3, 331–340. https://doi.org/10.1038/ncpneuro0504

Scott, W., Badenoch, J., Solar, M.G.C.M. del, Brown, D.A., Kemp, H., McCracken, L., Williams, A.C. de C., Rice, A.S., 2020. Acceptability of psychologically-based pain management and online delivery for people living with HIV and chronic neuropathic pain: a qualitative study. Scandinavian Journal of Pain.

Scott, W., Guildford, B.J., Badenoch, J., Driscoll, E., Chilcot, J., Norton, S., Kemp, H.I., Lee, M.J., Lwanga, J., Boffito, M., Moyle, G., Post, F.A., Campbell, L., Josh, J., Clift, P., Williams, A.C. de C., Rice, A., McCracken, L.M., 2021. Feasibility Randomized-Controlled Trial of Online Acceptance and Commitment Therapy for Painful Peripheral Neuropathy in People Living with HIV: The OPEN Study. Eur J Pain 25, 1493–1507. https://doi.org/10.1002/ejp.1762

Scott, W., Hann, K.E.J., McCracken, L.M., 2016. A Comprehensive Examination of Changes in Psychological Flexibility Following Acceptance and Commitment Therapy for Chronic Pain. J Contemp Psychother 46, 139–148. https://doi.org/10.1007/s10879-016-9328-5

Shafer, D., Gooing, J., Lee, V., Seffinger, M.A., 2020. Musculoskeletal Conditions in Patients With Diabetes: A Narrative Review. Journal of Osteopathic Medicine 120, 660–664. https://doi.org/10.7556/jaoa.2020.123

Singh, S.B., Singh, P.P., Singh, S., Aggarwal, R., Singh, J., 2012. Effect of Nerve Mobilization on Vibration Perception Threshold in Diabetic Peripheral Neuropathy. Indian Journal of Physiotherapy & Occupational Therapy-An International Journal 6, 189–195.

Skivington, K., Matthews, L., Simpson, S.A., Craig, P., Baird, J., Blazeby, J.M., Boyd, K.A., Craig, N., French, D.P., McIntosh, E., Petticrew, M., Rycroft-Malone, J., White, M., Moore, L., 2021. A new framework for developing and evaluating complex interventions: update of Medical Research Council guidance. BMJ 374, n2061. https://doi.org/10.1136/bmj.n2061

Sobhani, S., Asayesh, H., Sharifi, F., Djalalinia, S., Baradaran, H.R., Arzaghi, S.M., Mansourian, M., Rezapoor, A., Ansari, H., Masoud, M.P., 2014. Prevalence of diabetic peripheral neuropathy in Iran: a systematic review and meta-analysis. Journal of Diabetes & Metabolic Disorders 13, 1–7.

Song, X.-J., Huang, Z.-J., Song, W.B., Song, X.-S., Fuhr, A.F., Rosner, A.L., Ndtan, H., Rupert, R.L., 2016. Attenuation Effect of Spinal Manipulation on Neuropathic and Postoperative Pain Through Activating Endogenous Anti-Inflammatory Cytokine Interleukin 10 in Rat Spinal Cord. Journal of Manipulative and Physiological Therapeutics 39, 42–53. https://doi.org/10.1016/j.jmpt.2015.12.004

Sözen, T., Başaran, N.Ç., Tınazlı, M., Özışık, L., 2018. Musculoskeletal problems in diabetes mellitus. Eur J Rheumatol 5, 258–265. https://doi.org/10.5152/eurjrheum.2018.18044

Staniszewska, S., Brett, J., Simera, I., Seers, K., Mockford, C., Goodlad, S., Altman, D.G., Moher, D., Barber, R., Denegri, S., Entwistle, A., Littlejohns, P., Morris, C., Suleman, R., Thomas, V., Tysall, C., 2017. GRIPP2 reporting checklists: tools to improve reporting of patient and public involvement in research. BMJ 358, j3453. https://doi.org/10.1136/bmj.j3453

Tan, T., Barry, P., Reken, S., Baker, M., 2010. Pharmacological management of neuropathic pain in non-specialist settings: summary of NICE guidance. BMJ 340, c1079. https://doi.org/10.1136/bmj.c1079

Teixeira, E., 2010. The Effect of Mindfulness Meditation on Painful Diabetic Peripheral Neuropathy in Adults Older Than 50 Years. Holistic Nursing Practice 24, 277–283. https://doi.org/10.1097/HNP.0b013e3181f1add2

Themistocleous, A.C., Ramirez, J.D., Shillo, P.R., Lees, J.G., Selvarajah, D., Orengo, C., Tesfaye, S., Rice, A.S.C., Bennett, D.L.H., 2016. The Pain in Neuropathy Study (PiNS): a cross-sectional observational study determining the somatosensory phenotype of painful and painless diabetic neuropathy. Pain 157, 1132–1145. https://doi.org/10.1097/j.pain.0000000000000491

Tölle, T., Xu, X., Sadosky, A.B., 2006. Painful diabetic neuropathy: a cross-sectional survey of health state impairment and treatment patterns. Journal of Diabetes and its Complications 20, 26–33. https://doi.org/10.1016/j.jdiacomp.2005.09.007

Touma, C., Pannain, S., 2011. Does lack of sleep cause diabetes? Cleve Clin J Med 78, 549–558. https://doi.org/10.3949/ccjm.78a.10165

Underwood, M.R., Morton, V., Farrin, A., on behalf of the UK BEAM trial team, 2007. Do baseline characteristics predict response to treatment for low back pain? Secondary analysis of the UK BEAM dataset [ISRCTN32683578]. Rheumatology 46, 1297–1302. https://doi.org/10.1093/rheumatology/kem113

Vermeersch, A., Quaghebeur, J., 2018. The effect of an osteopathic treatment on the blood glucose level in diabetic patients. [WWW Document].

Wagner, J.A., Bermudez-Millan, A., Damio, G., Segura-Perez, S., Chhabra, J., Vergara, C., Feinn, R., Perez-Escamilla, R., 2016. A randomized, controlled trial of a stress management intervention for Latinos with type 2 diabetes delivered by community health workers: Outcomes for psychological wellbeing, glycemic control, and cortisol. Diabetes Research and Clinical Practice 120, 162–170. https://doi.org/10.1016/j.diabres.2016.07.022

Watson, J.A., Ryan, C.G., Cooper, L., Ellington, D., Whittle, R., Lavender, M., Dixon, J., Atkinson, G., Cooper, K., Martin, D.J., 2019. Pain Neuroscience Education for Adults With Chronic Musculoskeletal Pain: A Mixed-Methods Systematic Review and Meta-Analysis. J Pain 20, P1140.e1-1140.e2. https://doi.org/10.1016/j.jpain.2019.02.011

Whicher, C.A., O'Neill, S., Holt, R.I.G., 2020. Diabetes in the UK: 2019. Diabetic Medicine 37, 242–247. https://doi.org/10.1111/dme.14225

Williams, A.C. de C., Fisher, E., Hearn, L., Eccleston, C., 2020. Psychological therapies for the management of chronic pain (excluding headache) in adults. Cochrane Database of Systematic Reviews. https://doi.org/10.1002/14651858.CD007407.pub4

Yamamoto, J.M., Kellett, J.E., Balsells, M., García-Patterson, A., Hadar, E., Solà, I., Gich, I., Beek, E.M. van der, Castañeda-Gutiérrez, E., Heinonen, S., Hod, M., Laitinen, K., Olsen, S.F., Poston, L., Rueda, R., Rust, P., Lieshout, L. van, Schelkle, B., Murphy, H.R., Corcoy, R., 2018. Gestational Diabetes Mellitus and Diet: A Systematic Review and Meta-analysis of Randomized Controlled Trials Examining the Impact of Modified Dietary Interventions on Maternal Glucose Control and Neonatal Birth Weight. Diabetes Care 41, 1346–1361. https://doi.org/10.2337/dc18-0102

Ziegler, D., Tesfaye, S., Spallone, V., Gurieva, I., Al Kaabi, J., Mankovsky, B., Martinka, E., Radulian, G., Thy Nguyen, K., Stirban, A.O., Tankova, T., Varkonyi, T., Freeman, R., Kempler, P., Jm Boulton, A., 2021. Screening, diagnosis and management of diabetic sensorimotor polyneuropathy in clinical practice: International expert consensus recommendations. Diabetes Res Clin Pract 109063. https://doi.org/10.1016/j.diabres.2021.109063

A multimodal manual therapy-based intervention for people with painful diabetic neuropathy:

Development and description of the high-similarity control intervention (incl. treatment manual used during feasibility trial)

Authors and collaborators

David Hohenschurz-Schmidt* and the NeuOst working group

*** Chief investigator:**

Imperial College London, London, UK

University College of Osteopathy, London, UK

V.1.0, 15 March 2024

Conceptual background

Control intervention objectives

The objectives of the control intervention are to replicate the contextual elements of the test intervention (in other words, allow the study of the efficacy of intervention components of interest), and to blind participants to group allocation (thereby balancing expectancy and conditioning effects). The development of the control intervention follows the CoPPS Statement (Hohenschurz-Schmidt et al., 2023), and included co-development with practitioners and patient partners. As per CoPPS guidance, the control intervention resembles the tested intervention in all aspects but for the components whose effect the trial – or in this case a future full-scale trial - is designed to study.

Control intervention rationale and development

The control intervention was developed upon completion of the NeuOst intervention's development, following current best-practice guidance for control interventions in mechanistic and efficacy trials (Hohenschurz-Schmidt et al., 2023). This included conceptual discussions within the working group, and two practical piloting sessions with designated trial providers and the chief investigator and two people with lived experience.

First, the working group discussed which NeuOst treatment components were of interest to study in a full-scale efficacy trial. It was agreed that these components of interest were the ones for which there was a therapeutic rationale and mechanism of action, such as force parameters during manual therapy application, the elements of the exercise plan aimed at strengthening, stretching, and enhancing balance and cardiovascular fitness, and the elements of Acceptance and Commitment Therapy-derived techniques that aimed at enhancing cognitive flexibility. It was decided that, at this development stage, the combined effect of these intervention components was of greater scientific interest than their individual effects, but it was acknowledged that future intervention development and optimisation may wish to study isolated effects, for example in factorial designs (Collins et al., 2024; Ciolino et al., 2025). Together with working group subject experts, ideas for reducing or replacing these components of interest were explored. Components not of interest include: Attention from providers and a therapeutic relationship, fostered, for example, through the NeuOst intervention components of case history-taking, neurological and manual therapy examination, and complication screening. Disease education and lifestyle advice were also considered not of interest for study at this stage. Touch, including affective touch, and minimal joint or tissue movements are also not considered as components of interest in this study.

Secondly, following this conceptual development, people with lived experienced and designated trial provides were involved in the practical development of the control intervention. This included a one-to-one session of the chief investigator with a patient partner, where control and test interventions' manual treatment sequences were tested practically, asking for feedback and adapting approaches based on feedback. Similarly, draft materials for exercises and psychologically-informed approaches were reviewed, including their counterparts developed for the control intervention (see below). Next, upon completion of the NeuOst provider training course, designated trial providers joined a practical development session where manual therapy protocols were adapted in line with the conceptual parameters outlined above. As part of this workshop and the training webinar detailed below, the importance of matched behaviours, verbal and non-verbal communication, and of maintaining blinding were discussed and practised in training scenarios. Finally, a practical training and refinement session with trial providers and two people with lived experience took place, where the complete intervention and control intervention protocols were practised in small groups.

Provider input during co-development

During preparatory meetings and practice sessions with all designated tribe providers, providers also developed their own approach to delivering the controlled intervention within the pre-specified boundaries. They explored ways of delivering the control intervention that felt most authentic, credible, and acceptable to them. These approaches were then further refined in practice clinical appointments with patient partners and documented as part of a trial provider control intervention handbook. During those preparatory sessions, the trial providers also highlighted where they felt communication scripts may be useful and made proposals for their content.

Detailed description of control intervention

The session-by-session provider manual for the control intervention is provided at the end of the document.

Matched components

The control intervention matches the following features of the NeuOst intervention: the number, duration, and frequency of sessions; co-interventions; assessments and reassessments; follow-up contact frequency and mode; intervention standardisation and treatment protocol flexibility; fidelity monitoring; intervention tailoring (advice, communication, conversational topics, and physical areas treated are adapted to the patient in both trial arms); application modes and delivery formats; information regarding

intervention efficacy; thematic content of conversations and information material (except for differences in topics concerning the Acceptance and Commitment Therapy-derived components, as detailed below); body areas addressed; physical procedures performed (except for differences in manual therapy and exercise components, as detailed below); level of participant participation; procedural steps per session; equipment or tools employed (including provision of a ‘wobble cushion’ in both study arms); treatment ritual (through the same assessment and overall sequence of events from case history conversation, physical assessment, manual components above, to advice and good-bye); treatment-specific sensory cues (except for differences in sensation resulting from altered manual therapy components, as detailed below); treatment environment; personal interactions with therapy providers; and provider characteristics, including education, professional qualifications, experience, trial-specific training, and behaviour (including empathic listening and reassuring attitude and communication) – since all providers deliver interventions in both the NeuOst treatment and the control intervention arm.

Components of interest

Several components were decided to be ‘of interest’ during the conceptual development phase (Hohenschurz-Schmidt et al., 2023) and are thus not matched between NeuOst intervention and control intervention arms. Modifications (called ‘equivalents’ in the attached control intervention treatment manual), omitted components, and strategies to nonetheless achieve the control intervention’s objectives, are:

Manual therapy components:

Soft tissue techniques are performed with less force, aiming to move dermal layers only (this is based on the mechanistic assumption that muscles need to be stretched for soft tissue techniques to be effective; touch-based or potential lymphatic mechanisms are therefore not controlled for with this superficial approach and may still have effects).

Peripheral joint articulation / mobilisation techniques are performed with less amplitude (not approaching the end of range of the movements and staying within 50% of the total ROM). This assumes that articulatory techniques have effects through stimulation of stretch receptors located in periarticular tendons and joint capsules (Bialosky et al., 2009), and on neural function through improving blood flow (Cook et al., 2023).

Spinal articulation / mobilisation and manipulation techniques are not performed and replaced by the above superficial movement of dermal layers in spinal and paraspinal areas. This acknowledges that mechanistic studies of manual therapy do not support the importance of specific force parameters so that any force or movement applied to axial joints is controlled for instead (Bialosky et al., 2017; Nim et al., 2025).

Active resisted muscle activation in the lower extremities is not performed.

Neurodynamic techniques are performed with less amplitude (not approaching the end of range of the movements and staying within 50% of the total ROM). This assumes that mechanical stress during 'slider' and 'tensioner' techniques mobilises the nerve within its sheath (Cook et al., 2023).

Psychologically informed approaches:

ACT-derived approaches are modified to no longer target the supposed mechanisms but still resemble the original activity. Key mechanisms targeted in the NeuOst intervention and thus omitted in the control intervention are awareness, openness, values-driven action, and cognitive flexibility (McCracken and Morley, 2014).

Rather than exploring patients' towards and away-moves in response to pain (the original ACT worksheet in session no. 1), patients are asked to reflect on their pain experience and explore pain management strategies they can do by themselves or with the help of others; Metaphors are modified to no longer be relevant to creating 'openness' but resemble the original ACT metaphor. For example, the 'letting go' metaphor of trying to push a beach ball underneath the water is replaced with a reflection on different strategies of getting the ball out of the water.

Awareness exercises ('notice five things about your body') are modified to 'remember five things you can do when living with diabetes'. (both activities are from session 2).

In session 3, instead of exploring values and goals and setting a value-based SMART goal, patients are asked to remember 3-5 good experiences they had while experiencing pDPN symptoms. They are then invited to remember those while reflecting on their bodily sensations and emotions.

In session 4, instead of a mindfulness exercise to practice 'awareness', patients are asked to list symptoms they ascribe to their neuropathy. Together with the provider, they then explore possible pathophysiological explanations for those symptoms.

In the final session, instead of discussing how to plan for continued practice of the acquired skills, patients are asked to recapitulate the previous sessions and consider how they can "move forward from here", which barriers they might encounter, and how they could deal with those. Instead of providing guidance or advice, providers simply engage in reflective questioning.

In the intervention arm, providers can integrate interoceptive, mindfulness, rhythmic breathing, or other relaxation exercises into their manual therapy treatment. In control

intervention arm, they should limit themselves to encouraging patients to report what they are feeling or how pleasant or unpleasant a certain touch or body area feels.

Physical exercise:

Anaerobic stretch-and-strengthen programme: In the control intervention arm, participants are given unspecific advice and encouragement to “stretch and strengthen more, for example simply getting up out of chairs repeatedly or stretching the calves against a wall”. Exercises may be demonstrated briefly but are not rehearsed.

Aerobic activity is encouraged through unspecific advice and encouragement, such as “try to go for walks more often”, “try to take the stairs instead of the elevator if possible”, or “try to break a sweat more often”.

Apart from this, and instead of a specific training programme, a generic leaflet on the potential benefits of exercise is provided, including some exercise examples but no detailed programme. During treatment sessions, exercise compliance is not followed up beyond a simple verbal enquiry from the provider, and control intervention participants have no exercise diary to self-monitor their activities. (Note that physical activity levels are still monitored as part of the study’s data collection in all study arms.)

Protocol boundaries of control intervention

Both in the intervention and in the controlled intervention arm, the limits of what is permissible are established by the respective session by session protocols. Only obligatory or optional components listed in those protocols are within bounds.

It is noteworthy that treatment provider’s behaviour is not defined by the protocol, beyond ‘active listening’ and due to the use of an ACT-informed provider ‘stance’, both of which require the demonstration of compassion and a non-judgmental manner. Providers are required to employ the same verbal and nonverbal communication techniques and interpersonal behaviour in both the intervention and the controlled intervention arm.

Since the control intervention aims to produce the same level of positive expectations as the tested intervention, it is permissible to promote positive expectancy in the control intervention arm, albeit only to the level of what the provider also does in the intervention arm. In general, providers are encouraged to abstain from overly assured promises, which may be unbelievable to patients and are also not required since this is a research study where a certain level of uncertainty is presumed. However, positive framing, verbal and nonverbal encouragement, and talking about potential positive outcomes or desirable objectives of techniques and activities, can be used to produce positive and realistic expectations.

Communication scripts

How to introduce the therapeutic objectives and structure of the trials' interventions. (Session 1, protocol point 3)

With this treatment, we are hoping to help you to better manage your diabetic neuropathy symptoms and cope with them better. This treatment will involve 5 appointments with me, where we will talk about your symptoms but also about how they affect you and maybe about different ways you can look at them or deal with them. We will talk about different things that are important when you live with diabetic neuropathy, although I'm sure you'll be already aware of many of these issues. In each of those treatment sessions, you'll also have hands on treatment from me, where I mainly work on your legs and maybe back to help relieve some of your symptoms. We'd also be talking about the importance of physical activity and exercise from time to time. Before we start do you have any questions?

How to give generic exercise advice in the control arm.

We've discussed the importance of moving for diabetes management and possibly also to stop the progression of neuropathy symptoms. It might be good to try and increase your activity levels somewhat, for example by doing some cycling, going for a walk as a higher speed than usual, or doing some exercise at home or in the gym. Maybe try that until next week?

What if patients want more specific advice on exercises in the control arm?

Briefly demonstrate a simple calf stretch and/or hamstring stretch. If required, give generic advice, such as: *these exercises should be held for a few moments and performed regularly*. You can also add: *with regards to exercise that gets you breathless, you could for example a few flights of stairs go for a first walk around the block or do some dancing or running if you can. It really depends on what you like to do, the important thing is that you get your heart rate up and maybe break a little sweat.*

What if a patient asks for a more forceful or larger range of motion approach during the manual therapy component of the control intervention?

There are many different osteopathic techniques, some of which are quite forceful, while others are quite gentle and more specific. This research study aims to investigate the more subtle approaches, which is why I'm not able to change what I do with my hands too much. I am however choosing my techniques depending on what I feel where it might be useful to treat you. I'm sure this will also be beneficial to you.

What if a patient asks why we're talking about a certain topic or performing a certain hands-on technique?

Here it is important to provide an explanation. Usually these can be held generic and borrowed from the rationale in the intervention arm. For example, when performing the control intervention's ACT activities, you may wish to point out that talking about one's experience can produce important new insights and acknowledge that it might not always feel comfortable at first. For the control intervention manual therapy techniques, one could explain that they aim to provide sensory input, relaxation, and movement, which may or may not reduce discomfort or enable patients to move better.

Providers and provider training

For designated trial providers, the NeuOst training course (see NeuOst intervention protocol) included two obligatory lectures on 1) the Fundamentals of Nonpharmacological Intervention Research and of Placebo Controlled Trials, and 2) the NeuOst Trial Protocol and Considerations for Trial Providers, delivered by the chief investigator. This covered the following learning objectives and content. Course completion was ensured by the chief investigator and understanding was tested by means of a quiz.

Talk 1: The Fundamentals of Nonpharmacological Intervention Research and of Placebo Controlled Trials (Duration: 0.5 hours, Delivery mode: Recorded)

Learning objectives:

- Appreciate the challenges of nonpharmacological / complex intervention clinical research, especially in so-called efficacy trials.
- Appreciate the potential for placebo effects in physical and psychological interventions and their implications for trial design.
- Understand the concepts of blinding and control interventions in efficacy trials.
- Know about current best-practice frameworks for control intervention design.

Content:

- 30-mins recording of a webinar held for the British Pain Society (full recording: https://www.youtube.com/watch?v=WI4jaqf5_PE)
- Webinar includes an optional 30-mins talk by Prof David Beard on placebo-controlled surgery trials and a panel discussion.

Talk 2: The NeuOst Trial Protocol and Considerations for Trial Providers (Duration: 0.5 hours, Delivery mode: Synchronous e-learning - live delivery after in-practice training day for selected trial providers)

Learning objectives:

- Understand the basic design elements NeuOst feasibility trial.
- Appreciate the tasks and role of clinical providers in an RCT in general and in NeuOst in particular.
- Reflect on and discuss concerns and challenges as trial providers.
- Gain access to materials for providers, such as detailed session protocol and ACT handbook.
- Commence practical feasibility trial participation planning, such as populating weekly diaries.

Content:

- Key design elements of the NeuOst feasibility trial
- Role and responsibilities of clinical trial providers in sham-controlled trials in general and in the NeuOst feasibility trial in particular.
- Experiences of trial providers from relevant related RCTs.
- Relevant ethical considerations.
- Reflection and discussion amongst participants.
- Training tasks.

Finally, designated trial providers attended a practical 2-hour session with two patient partners, where the complete intervention and control intervention protocols were practised in small groups. Afterwards, providers reflected on the experience, facilitated by the chief investigator.

In between individual training sessions, providers were encouraged to revisit theoretical materials and practice their NeuOst-related skills with their own patients, friends, or colleagues. Trial processes and (control) intervention delivery were refined based on experiences during this work with designated trial providers. This included the development of conversation scripts for clinical appointments (see above). As part of this preparation, trial providers were also trained to recognise the need for a control intervention and maintaining participant blinding in this research study. During the trial, trial providers communicate with one another about challenges in control intervention delivery and attend regular supervision and troubleshooting sessions with the chief investigator and the working group's clinical psychologist.

Fidelity monitoring in control intervention arm

Fidelity monitoring processes are the same as in the intervention arm (NeuOst feasibility trial protocol section 8.13), except that the below (control) intervention protocol will be used for fidelity checking of audio- and video recordings and for provider self-report of delivered intervention components.

Control intervention protocol per trial session

**Control Intervention content** (providers decide the sequence per session)<br><br>Components marked 'optional' are non-obligatory; all others must be delivered as part of the respective treatment session.	Fidelity checklist (to be completed by trial providers)	**Notes** (Further detail such as amount and duration or provider experiences such as problems and opportunities)
Session 1		Session duration up to 90 mins
Communication elements:<br>10. Active listening to patient narrative<br>11. Initial case history<br>12. Introduction to the therapeutic objectives and structure of NeuOst<br>13. Acknowledgement of medication list<br>14. Conversation about medications (optional)<br>15. Communication with primary care / specialist care provider (optional)<br>16. Signposting to DM/NeuP-related care options (optional)<br>17. Exploration of patient beliefs (regarding pain and disease) (optional)<br>18. Education about pain and neuropathic pain, including management strategies, sleep management, and devices (optional)<br><br>ACT-informed elements:<br>5. Exploration of possible self-help and external support strategies (via Session 1 ACT worksheet)<br>6. Exploration of patient feelings (via Session 1 ACT worksheet)<br>7. Explorations of additional, personally-relevant self-help or support strategies (via Session 1 ACT worksheet)	Comms:<br>10. ☐<br>11. ☐<br>12. ☐<br>13. ☐<br>14. ☐<br>15. ☐<br>16. ☐<br>17. ☐<br>18. ☐<br><br>ACT:<br>5. ☐<br>6. ☐<br>7. ☐	Up to 30 mins
Manual therapy:<br>**13. Assessment** (inspection, active & passive joint movement, palpation)<br>**14. Peripheral joint articulation equivalent** (feet & ankles at least 2mins per side; then knees & hips any duration)	MT:<br>13. ☐<br>14. ☐	Up to 20 mins

15. Other peripheral joint articulation equivalents (optional)<br>16. Spinal joint articulation equivalents (optional)<br>17. Peripheral soft tissue manipulation equivalent (gastrocnemius at least 1min per side)<br>18. Other peripheral soft tissue manipulation equivalents (optional)<br>19. Paraspinal soft tissue manipulation equivalents (optional)<br>20. **Active muscle activation** (lower extremity: dorsiflexion, plantarflexion, knee flexion and knee extension, at least 10 times each per side, hold 5 seconds per rep.)<br>21. Neurodynamic technique lower extremity equivalent (active; 10 reps per side)<br>22. 'Subtle' osteopathic technique equivalents (optional)<br>23. Integration of patient breathing, relaxation, interoceptive awareness ("notice" and "open"), and/or activity-exploration (ACT-equivalents based) into manual treatment (optional)	15. ☐<br>16. ☐<br>17. ☐<br>18. ☐<br>19. ☐<br>20. ☐<br>21. ☐<br>22. ☐<br>23. ☐	
Neurological:<br>2. **Provider baseline neurological examination** (in addition to research-related baseline testing; not used as outcome)	Neuro:<br>2. ☐	Up to 10 mins
Physical activity:<br>11. Raise the topic of physical activity with permission (use motivational interviewing techniques throughout) (5As': "Ask")<br>12. Evaluation of current physical activity levels ("Assess")<br>13. Explore barriers to more engagement in physical activity<br>14. Educate about benefits of different types of physical activity ("Advise")<br>**15. Provide "Exercise and diabetes" handout** (by the Diabetes Research & Wellness Foundation) ("Advise")	Exx:<br>11. ☐<br>12. ☐<br>13. ☐<br>14. ☐<br>15. ☐<br>16. ☐	Up to 20 mins

16. **Values-based goal setting for aerobic exercise activity** (patient choice of activity, use Borg scale for exertion level) ("Agree")<br>17. Give general advice on possible stretch, strength, or stability exercises.<br>18. Decide on suitable modifications for exercises (optional)<br>19. **Provide "wobble cushion"** ("Arrange") (provided as part of trial)	17. ☐<br>18. ☐<br>19. ☐	
Diabetes related:<br>**6. Diabetes complication screening** (red flags for foot health, vision changes, kidney disease etc.)<br>7. Conversation about dietary DM management (optional)<br>8. Education about DM (optional)<br>9. Education about DPN and other complications of DM, including management strategies (optional)<br>10. Provision of NeuOst educational materials (optional)	DM/DPN:<br>6. ☐<br>7. ☐<br>8. ☐<br>9. ☐<br>10. ☐	Up to 10 mins

PROVIDERS TO COMPLETE AT END OF SESSION:

Please rate your agreement with the following statement with regards to today's intervention session for the management of the patient's painful diabetic neuropathy symptoms.

This treatment will be completely effective.

Strongly disagree	Moderately disagree	Slightly disagree	Neither agree nor disagree	Slightly agree	Moderately agree	Strongly agree

Session 2		45-60 mins duration
Communication:<br>8. Active listening to patient narrative<br>9. Follow-up case history<br>10. Conversation about medications (optional)<br>11. Communication with primary care / specialist care provider (optional)<br>12. Signposting to DM/NeuP-related care options (optional)<br>13. Exploration of patient beliefs (regarding pain and disease) (optional)<br>14. Education about pain and neuropathic pain, including management strategies, sleep management, and devices (optional)<br><br>ACT-informed elements:<br>5. Revisiting last week's exploration of self-help and external help strategies<br>6. Exploring the problem with doing it alone "Beach ball metaphor" (via Session 2 ACT worksheet)<br>7. "5 things to do when you live with diabetes" exercise (via Session 2 ACT worksheet)<br>8. Encouragement to practice these activities at home	8. ☐<br>9. ☐<br>10. ☐<br>11. ☐<br>12. ☐<br>13. ☐<br>14. ☐<br><br>5. ☐<br>6. ☐<br>7. ☐<br>8. ☐	Up to 20 mins
Manual therapy:<br>**1. Assessment** (inspection, active & passive joint movement, palpation)<br>**2. Peripheral joint articulation equivalent** (feet & ankles at least 2mins per side; then knees & hips any duration)<br>3. Other peripheral joint articulation equivalents (optional)<br>4. Spinal joint articulation equivalents (optional)<br>5. Peripheral soft tissue manipulation equivalent (gastrocnemius at least 1min per side)<br>6. Other peripheral soft tissue manipulation equivalents (optional)<br>7. Paraspinal soft tissue manipulation equivalents (optional)<br>8. **Active muscle activation** (lower extremity: dorsiflexion, plantarflexion, knee flexion and knee extension, at least 10 times each per side, hold 5 seconds per rep.)	MT:<br>1. ☐<br>2. ☐<br>3. ☐<br>4. ☐<br>5. ☐<br>6. ☐<br>7. ☐<br>8. ☐<br>9. ☐<br>10. ☐<br>11. ☐	Up to 20 mins

9. Neurodynamic technique lower extremity equivalent (active; 10 reps per side)<br>10. ‘Subtle’ osteopathic technique equivalents (optional)<br>11. Integration of patient breathing, relaxation, interoceptive awareness (“notice” and “open”), and/or activity-exploration (ACT- equivalents based) into manual treatment (optional)		
Neurological:<br>2. Follow-up neurological examination (if indicated, optional)	2. ☐	
Physical activity:<br>8. Revisit experiences and/or exercises of performed / advised at last session<br>9. Explore if exercises and stretches can now be performed at greater difficulty level or through larger range of movement<br>10. Reinforce importance of exercise (optional)<br>11. Explore barriers to adherence and provide advice on overcoming those (optional)<br>12. Decide on suitable modifications for exercise activities (optional)	8. ☐<br>9. ☐<br>10. ☐<br>11. ☐<br>12. ☐	Up to 10 mins
Diabetes management and education:<br>7. Foot care education and practical run-through (under guidance of below handout)<br>8. **Provide foot care handout** (“Diabetes and looking after your feet” from Diabetes UK)<br>9. Conversation about dietary DM management (optional)<br>10. Education about DM (optional)<br>11. Education about DPN and other complications of DM, including management strategies (optional)<br>12. Provision of other NeuOst educational materials (optional)	7. ☐<br>8. ☐<br>9. ☐<br>10. ☐<br>11. ☐<br>12. ☐	Up to 10 mins
PROVIDERS TO COMPLETE AT END OF SESSION:<br><br>Please rate your agreement with the following statement with regards to today’s intervention session for the management of the patient’s painful diabetic neuropathy symptoms.		

This treatment will be completely effective.

Strongly disagree	Moderately disagree	Slightly disagree	Neither agree nor disagree	Slightly agree	Moderately agree	Strongly agree

Session 3		45-60 mins duration
Communication:<br>8. Active listening to patient narrative<br>9. Follow-up case history<br>10. Conversation about medications (optional)<br>11. Communication with primary care / specialist care provider (optional)<br>12. Signposting to DM/NeuP-related care options (optional)<br>13. Exploration of patient beliefs (regarding pain and disease) (optional)<br>14. Education about pain and neuropathic pain, including management strategies, sleep management, and devices (optional)<br>ACT-informed elements:<br>4. Reviewing patient experiences with "5 Things to do when you have diabetes" exercise<br>5. Providing information on positive experiences (via Session 3 ACT worksheet)<br>6. Completing "Experience Compass" and planning on recreating positive experiences (Session 3 ACT worksheet)	8. ☐<br>9. ☐<br>10. ☐<br>11. ☐<br>12. ☐<br>13. ☐<br>14. ☐<br>4. ☐<br>5. ☐<br>6. ☐	Up to 30 mins
Manual therapy:<br>**1. Assessment** (inspection, active & passive joint movement, palpation)<br>**2. Peripheral joint articulation equivalent** (feet & ankles at least 2mins per side; then knees & hips any duration)<br>3. Other peripheral joint articulation equivalents (optional)<br>4. Spinal joint articulation equivalents (optional)<br>5. Peripheral soft tissue manipulation equivalent (gastrocnemius at least 1min per side)<br>6. Other peripheral soft tissue manipulation equivalents (optional)<br>7. Paraspinal soft tissue manipulation equivalents (optional)<br>8. **Active muscle activation** (lower extremity: dorsiflexion, plantarflexion, knee flexion and knee	MT:<br>1. ☐<br>2. ☐<br>3. ☐<br>4. ☐<br>5. ☐<br>6. ☐<br>7. ☐<br>8. ☐<br>9. ☐	Up to 20 mins

extension, at least 10 times each per side, hold 5 seconds per rep.)<br>9. Neurodynamic technique lower extremity equivalent (active; 10 reps per side)<br>10. 'Subtle' osteopathic technique equivalents (optional)<br><br>Integration of patient breathing, relaxation, interoceptive awareness ("notice" and "open"), and/or activity-exploration (ACT- equivalents based) into manual treatment (optional)	10. ☐	
Neurological:<br>2. Follow-up neurological examination (if indicated, optional)	2. ☐	
Physical activity:<br>1. Revisit experiences and/or exercises of performed / advised at last session<br>2. Explore if exercises and stretches can now be performed at greater difficulty level or through larger range of movement<br>3. Reinforce importance of exercise (optional)<br>4. Explore barriers to adherence and provide advice on overcoming those (optional)<br>5. Decide on suitable modifications for exercise activities (optional)	1. ☐<br>2. ☐<br>3. ☐<br>4. ☐<br>5. ☐	Up to 10 mins
Diabetes management and education:<br>6. Revisit content and experiences with foot care education and handout<br>7. Conversation about dietary DM management (optional)<br>8. Education about DM (optional)<br>9. Education about DPN and other complications of DM, including management strategies (optional)<br>10. Provision of other NeuOst educational materials (optional)	6. ☐<br>7. ☐<br>8. ☐<br>9. ☐<br>10. ☐	Up to 10 mins
PROVIDERS TO COMPLETE AT END OF SESSION:<br><br>Please rate your agreement with the following statement with regards to today's intervention session for the management of the patient's painful diabetic neuropathy symptoms.<br><br>This treatment will be completely effective.		

Strongly disagree	Moderately disagree	Slightly disagree	Neither agree nor disagree	Slightly agree	Moderately agree	Strongly agree

Session 4		45-60 mins duration
Communication:<br>8. Active listening to patient narrative<br>9. Follow-up case history<br>10. Conversation about medications (optional)<br>11. Communication with primary care / specialist care provider (optional)<br>12. Signposting to DM/NeuP-related care options (optional)<br>13. Exploration of patient beliefs (regarding pain and disease) (optional)<br>14. Education about pain and neuropathic pain, including management strategies, sleep management, and devices (optional)<br><br>ACT-informed elements:<br>4. Revisiting last week's positive experiences plan (incl. any progress or barriers)<br>5. Conducting the "Neuropathic Pain Educational exercise" (via Session 4 ACT worksheet)	8. ☐<br>9. ☐<br>10. ☐<br>11. ☐<br>12. ☐<br>13. ☐<br>14. ☐<br><br>4. ☐<br>5. ☐	Up to 20 mins
Manual therapy:<br>1. **Assessment** (inspection, active & passive joint movement, palpation)<br>2. **Peripheral joint articulation equivalent** (feet & ankles at least 2mins per side; then knees & hips any duration)<br>3. Other peripheral joint articulation equivalents (optional)<br>4. Spinal joint articulation equivalents (optional)<br>5. Peripheral soft tissue manipulation equivalent (gastrocnemius at least 1min per side)<br>6. Other peripheral soft tissue manipulation equivalents (optional)<br>7. Paraspinal soft tissue manipulation equivalents (optional)<br>8. **Active muscle activation** (lower extremity: dorsiflexion, plantarflexion, knee flexion and knee extension, at least 10 times each per side, hold 5 seconds per rep.)<br>9. Neurodynamic technique lower extremity equivalent (active; 10 reps per side)	MT:<br>1. ☐<br>2. ☐<br>3. ☐<br>4. ☐<br>5. ☐<br>6. ☐<br>7. ☐<br>8. ☐<br>9. ☐<br>10. ☐	Up to 20 mins

10. 'Subtle' osteopathic technique equivalents (optional)<br><br>Integration of patient breathing, relaxation, interoceptive awareness ("notice" and "open"), and/or activity-exploration (ACT- equivalents based) into manual treatment (optional)		
Neurological:<br>2. Follow-up neurological examination (if indicated, optional)	2. ☐	
Physical activity:<br>1. Revisit experiences and/or exercises of performed / advised at last session<br>2. Explore if exercises and stretches can now be performed at greater difficulty level or through larger range of movement<br>3. Reinforce importance of exercise (optional)<br>4. Explore barriers to adherence and provide advice on overcoming those (optional)<br>5. Decide on suitable modifications for exercise activities (optional)	1. ☐<br>2. ☐<br>3. ☐<br>4. ☐<br>5. ☐	Up to 10 mins
Diabetes management and education:<br>5. Conversation about dietary DM management (optional)<br>6. Education about DM (optional)<br>7. Education about DPN and other complications of DM, including management strategies (optional)<br>8. Provision of other NeuOst educational materials (optional)	5. ☐<br>6. ☐<br>7. ☐<br>8. ☐	Up to 10 mins

PROVIDERS TO COMPLETE AT END OF SESSION:

Please rate your agreement with the following statement with regards to today's intervention session for the management of the patient's painful diabetic neuropathy symptoms.

This treatment will be completely effective.

Strongly disagree	Moderately disagree	Slightly disagree	Neither agree	Slightly agree	Moderately agree	Strongly agree

			nor disagree			

Session 5		45-60 mins duration
Communication:<br>8. Active listening to patient narrative<br>9. Follow-up case history<br>10. Conversation about medications (optional)<br>11. Communication with primary care / specialist care provider (optional)<br>12. Signposting to DM/NeuP-related care options (optional)<br>13. Exploration of patient beliefs (regarding pain and disease) (optional)<br>14. Education about pain and neuropathic pain, including management strategies, sleep management, and devices (optional)<br><br>ACT-informed elements:<br>5. Exploring thoughts on the Neuropathic Pain education since last session<br>**6. Reviewing any skills practice patient may be performing** (incl. impact on life, opportunities for further practice, barriers and how to manage those moving forward)<br>7. Review session 3 “things to do when living with diabetes” and “positive experiences” activities (incl. any progress towards that)<br>8. Discussing how to deal with difficulties	8. ☐<br>9. ☐<br>10. ☐<br>11. ☐<br>12. ☐<br>13. ☐<br>14. ☐<br><br>5. ☐<br>6. ☐<br>7. ☐<br>8. ☐	Up to 20 mins
Manual therapy:<br>**1. Assessment** (inspection, active & passive joint movement, palpation)<br>**2. Peripheral joint articulation equivalent** (feet & ankles at least 2mins per side; then knees & hips any duration)<br>3. Other peripheral joint articulation equivalents (optional)<br>4. Spinal joint articulation equivalents (optional)<br>5. Peripheral soft tissue manipulation equivalent (gastrocnemius at least 1min per side)<br>6. Other peripheral soft tissue manipulation equivalents (optional)<br>7. Paraspinal soft tissue manipulation equivalents (optional)<br>8. **Active muscle activation** (lower extremity: dorsiflexion, plantarflexion, knee flexion and	MT:<br>1. ☐<br>2. ☐<br>3. ☐<br>4. ☐<br>5. ☐<br>6. ☐<br>7. ☐<br>8. ☐<br>9. ☐	Up to 20 mins

knee extension, at least 10 times each per side, hold 5 seconds per rep.)<br>9. Neurodynamic technique lower extremity equivalent (active; 10 reps per side)<br>10. 'Subtle' osteopathic technique equivalents (optional)<br>11. Integration of patient breathing, relaxation, interoceptive awareness ("notice" and "open"), and/or activity-exploration (ACT- equivalents based) into manual treatment (optional)	10. ☐<br>11. ☐	
Neurological:<br>2. Follow-up neurological examination (this week not optional)<br>(for providers; not used as outcome during research study)	2. ☐	Up to 15 mins
Physical activity:<br>1. Revisit experiences and/or exercises of performed / advised at last session<br>2. Explore if exercises and stretches can now be performed at greater difficulty level or through larger range of movement<br>3. Reinforce importance of exercise (optional)<br>4. Explore barriers to adherence and provide advice on overcoming those (optional)<br>5. Decide on suitable modifications for exercise activities (optional)	1. ☐<br>2. ☐<br>3. ☐<br>4. ☐<br>5. ☐	Up to 10 mins
Diabetes management and education:<br>6. Explore patient's plans for future DM and painful DPN management<br>7. Conversation about dietary DM management (optional)<br>8. Education about DM (optional)<br>9. Education about DPN and other complications of DM, including management strategies (optional)<br>10. Provision of other NeuOst educational materials (optional)	6. ☐<br>7. ☐<br>8. ☐<br>9. ☐<br>10. ☐	Up to 10 mins
PROVIDERS TO COMPLETE AT END OF SESSION:		

Please rate your agreement with the following statement with regards to today's intervention session for the management of the patient's painful diabetic neuropathy symptoms.

This treatment will be completely effective.

Strongly disagree	Moderately disagree	Slightly disagree	Neither agree nor disagree	Slightly agree	Moderately agree	Strongly agree

End of programme

References SDC part 4

Bialosky, J.E., Beneciuk, J.M., Bishop, M.D., Coronado, R.A., Penza, C.W., Simon, C.B., George, S.Z., 2017. Unraveling the Mechanisms of Manual Therapy: Modeling an Approach. J Orthop Sports Phys Ther 48, 8–18. https://doi.org/10.2519/jospt.2018.7476

Bialosky, J.E., Bishop, M.D., Price, D.D., Robinson, M.E., George, S.Z., 2009. The mechanisms of manual therapy in the treatment of musculoskeletal pain: A comprehensive model. Manual Therapy 14, 531–538. https://doi.org/10.1016/j.math.2008.09.001

Ciolino, J.D., Scholtens, D.M., Bonner, L.B., 2025. Factorial Clinical Trial Designs. JAMA 333, 532–533. https://doi.org/10.1001/jama.2024.25374

Collins, L.M., Nahum-Shani, I., Guastaferro, K., Strayhorn, J.C., Vanness, D.J., Murphy, S.A., 2024. Intervention Optimization: A Paradigm Shift and Its Potential Implications for Clinical Psychology. Annual Review of Clinical Psychology 20, 21–47. https://doi.org/10.1146/annurev-clinpsy-080822-051119

Cook, C.E., Rhon, D.I., Bialosky, J., Donaldson, M., George, S.Z., Hall, T., Kawchuk, G., Lane, E., Lavazza, C., Lluch, E., Louw, A., Mazzieri, A.M., McDevitt, A., Reed, W.R., Schmid, A.B., Silva, A.G., Smart, K.M., Puentedura, E.J., 2023. Developing Manual Therapy Frameworks for Dedicated Pain Mechanism. JOSPT Open 1, 48–62. https://doi.org/10.2519/josptopen.2023.0002

Hohenschurz-Schmidt, D., Vase, L., Scott, W., Annoni, M., Ajayi, O.K., Barth, J., Bennell, K., Berna, C., Bialosky, J., Braithwaite, F., Finnerup, N.B., Williams, A.C. de C., Carlino, E., Cerritelli, F., Chaibi, A., Cherkin, D., Colloca, L., Côté, P., Darnall, B.D., Evans, R., Fabre, L., Faria, V., French, S., Gerger, H., Häuser, W., Hinman, R.S., Ho, D., Janssens, T., Jensen, K., Johnston, C., Lunde, S.J., Keefe, F., Kerns, R.D., Koechlin, H., Kongsted, A., Michener, L.A., Moerman, D.E., Musial, F., Newell, D., Nicholas, M., Palermo, T.M., Palermo, S., Peerdeman, K.J., Pogatzki-Zahn, E.M., Puhl, A.A., Roberts, L., Rossettini, G., Matthiesen, S.T., Underwood, M., Vaucher, P., Vollert, J., Wartolowska, K., Weimer, K., Werner, C.P., Rice, A.S.C., Draper-Rodi, J., 2023. Recommendations for the development, implementation, and reporting of control interventions in efficacy and mechanistic trials of physical, psychological, and self-management therapies: the CoPPS Statement. BMJ 381, e072108. https://doi.org/10.1136/bmj-2022-072108

McCracken, L.M., Morley, S., 2014. The Psychological Flexibility Model: A Basis for Integration and Progress in Psychological Approaches to Chronic Pain Management. The Journal of Pain 15, 221–234. https://doi.org/10.1016/j.jpain.2013.10.014

Nim, C., Aspinall, S.L., Cook, C.E., Corrêa, L.A., Donaldson, M., Downie, A.S., Harsted, S., Hansen, S., Jenkins, H.J., McNaughton, D., Nyirö, L., Perle, S.M., Roseen, E.J., Young, J.J., Young, A., Zhao, G.-H., Hartvigsen, J., Juhl, C.B., 2025. The Effectiveness of Spinal Manipulative Therapy in Treating Spinal Pain Does Not Depend on the Application Procedures: A Systematic Review and Network Meta-analysis. Journal of Orthopaedic & Sports Physical Therapy 55, 109–122. https://doi.org/10.2519/jospt.2025.12707
